# Assembly-graph-assisted haplotype-resolved scaffolding of polyploid genomes

**DOI:** 10.1038/s41477-026-02422-5

**Published:** 2026-10-16

**Authors:** Wenjie Du, Yanchun Li, Xiaofei Zeng, Jinbao Yang, Xingbin Wang, Xiaohui Zhang, Lianguang Shang, Xiaopeng Fu, Xiaoni Zhang, Xiu-qing Wei, Tingcai Cheng, Dongxi Li, Zhiqiang Wu, Weihua Pan

**Affiliations:** 1 Shenzhen Branch, Guangdong Laboratory of Lingnan Modern Agriculture, Key Laboratory of Synthetic Biology, Ministry of Agriculture and Rural Affairs, Genome Analysis Laboratory of the Ministry of Agriculture and Rural Affairs, State Key Laboratory of Genome and Multi-omics Technologies, State Key Laboratory of Tropical Crop Breeding, Agricultural Genomics Institute at Shenzhen, Chinese Academy of Agricultural Sciences, Shenzhen, China; 2 State Key Laboratory of Resource Insects, Southwest University, Chongqing, China; 3 College of Computer Science and Technology, Taiyuan University of Technology, Taiyuan, China; 4 National Key Laboratory for Germplasm Innovation & Utilization of Horticultural Crops, College of Horticulture and Forestry Sciences, Huazhong Agricultural University, Wuhan, China; 5 Fruit Research Institute, Fujian Academy of Agricultural Sciences, Fuzhou, China

**Keywords:** Bioinformatics, Plant sciences

## Abstract

Assembling polyploid genomes at the chromosome level remains a major challenge due to the difficulty of accurately distinguishing homologous chromosomes, leaving a vast number of polyploid species without high-quality genome sequences. Current chromatin conformation capture (3C)-based haplotype-resolved scaffolding methods often produce highly incomplete and mis-phased assemblies of complex polyploids, as short sequences lacking sufficient interaction support and collapsed regions with misleading signals are difficult to resolve. Here we present GPhase, a haplotype-resolved scaffolding algorithm that integrates 3C (for example, Hi-C, Pore-C and Omni-C) data with assembly graphs to enhance accuracy, completeness and contiguity. By leveraging graph connectivity derived from long-read overlaps, GPhase effectively overcomes key limitations of 3C. When applied to 15 complex polyploid genomes, GPhase reduced the sequence missing rate (BUSCO-based) by up to tens-fold, decreased the sequence error rate (*k*-mer-based) by up to an order of magnitude and improved the contiguity (N50) of gap-free sequences by several-fold compared with existing tools.

## Abstract (web summary)

This study presents GPhase, a haplotype-resolved scaffolding algorithm that leverages assembly graphs to overcome key limitations of 3C data and substantially improve the accuracy, completeness and contiguity of complex polyploid genome assembly.

## Main

Accurate and complete genome sequences are fundamental for investigating the inheritance, evolution and trait architecture of organisms. In recent years, advances in sequencing technologies have greatly reduced the difficulty of diploid genome assembly, and in most cases, haplotype-resolved, chromosome-scale assemblies can now be routinely generated using standardized pipelines^1^. In contrast, haplotype-resolved assembly of polyploid genomes remains highly challenging due to factors such as larger genome sizes, higher proportions of repetitive sequences and the high yet uneven sequence similarity among homologous chromosomes, all of which substantially complicate the discrimination of homologous copies^2^. Polyploidy is widespread in nature, occurring in both plants and animals. In plants, it is particularly common, with approximately 70% of angiosperms showing evidence of polyploid ancestry^3,4^. However, among currently available high-quality genome assemblies, polyploid genomes account for only about 18% (ref. ^3^). This discrepancy highlights the substantial challenges associated with polyploid genome assembly and underscores the need for novel strategies capable of accurately and completely resolving these complex genomes.

The haplotype-resolved assembly process for polyploid genomes typically involves two steps: (1) assembling long reads into unitigs (unique contigs) and (2) leveraging additional information to perform unitig phasing, ordering and orientation (haplotype-resolved scaffolding). While the first step is analogous to diploid assembly and can be accomplished using general-purpose assemblers such as hifiasm^5–7^, Verkko^8^, Flye^9^ and NextDenovo^10^, the main challenge lies in the second step, which requires high-quality linkage data to assign unitigs to haplotypes and arrange them into chromosome-level structures. In haplotype-resolved scaffolding, accurately clustering unitigs by haplotype is particularly critical. Several strategies have been developed to address this challenge. Gamete-based approaches^11,12^, for instance, sequence hundreds of individual gametes to derive unitig coverage profiles, grouping unitigs with similar profiles into the same haplotype^13^. Genome-wide maps—including genetic^14^, physical^15^, optical maps^16^ and chromatin conformation capture (3C) maps (for example, Hi-C^17,18^, Pore-C^19^ and Omni-C)—also provide linkage information useful for distinguishing intra- from inter-haplotype connections. Among these, 3C maps serve as the primary source of linkage information in most haplotype-resolved scaffolding studies, due to their favourable balance of cost, scalability and effectiveness^1,20–23^. Among 3C-based data types, Hi-C remains the most widely used, while emerging approaches such as Pore-C and Omni-C are increasingly being adopted.

Currently, two 3C-based scaffolding tools are available for polyploid genomes: ALLHiC^24^ and HapHiC^25^, both of which were originally designed for Hi-C data but can also be applied, to a limited extent, to other 3C-based data types. These tools have partially addressed the challenge of haplotype-resolved scaffolding in polyploid genomes, and most recently assembled polyploid genomes have relied on them^26–29^. Nevertheless, significant challenges remain due to inherent limitations of 3C data. First, as the most widely used 3C data type for scaffolding, Hi-C provides only coarse-grained linkage information. Short unitigs often lack restriction enzyme cut sites, leaving them unlinked by Hi-C data, while uneven sequencing coverage produces sparse signals across certain regions. Such unitigs are frequently discarded, leading to incomplete chromosome assemblies. Second, homologous regions with high sequence similarity are often collapsed into consensus sequences during unitig assembly. These collapsed unitigs typically exhibit strong Hi-C signals with multiple haplotypes, which can mislead clustering and result in uneven haplotype assemblies. Although emerging 3C-derived data types, such as Pore-C and Omni-C, offer theoretical advantages—owing to longer read lengths or independence from restriction enzyme cut sites that may partially alleviate issues such as short-unitig assignment—they still provide fundamentally coarse-grained linkage information. Consequently, they cannot intrinsically resolve the core challenges of haplotype-aware scaffolding in polyploid genomes.

**Extended Data Fig. 1:**
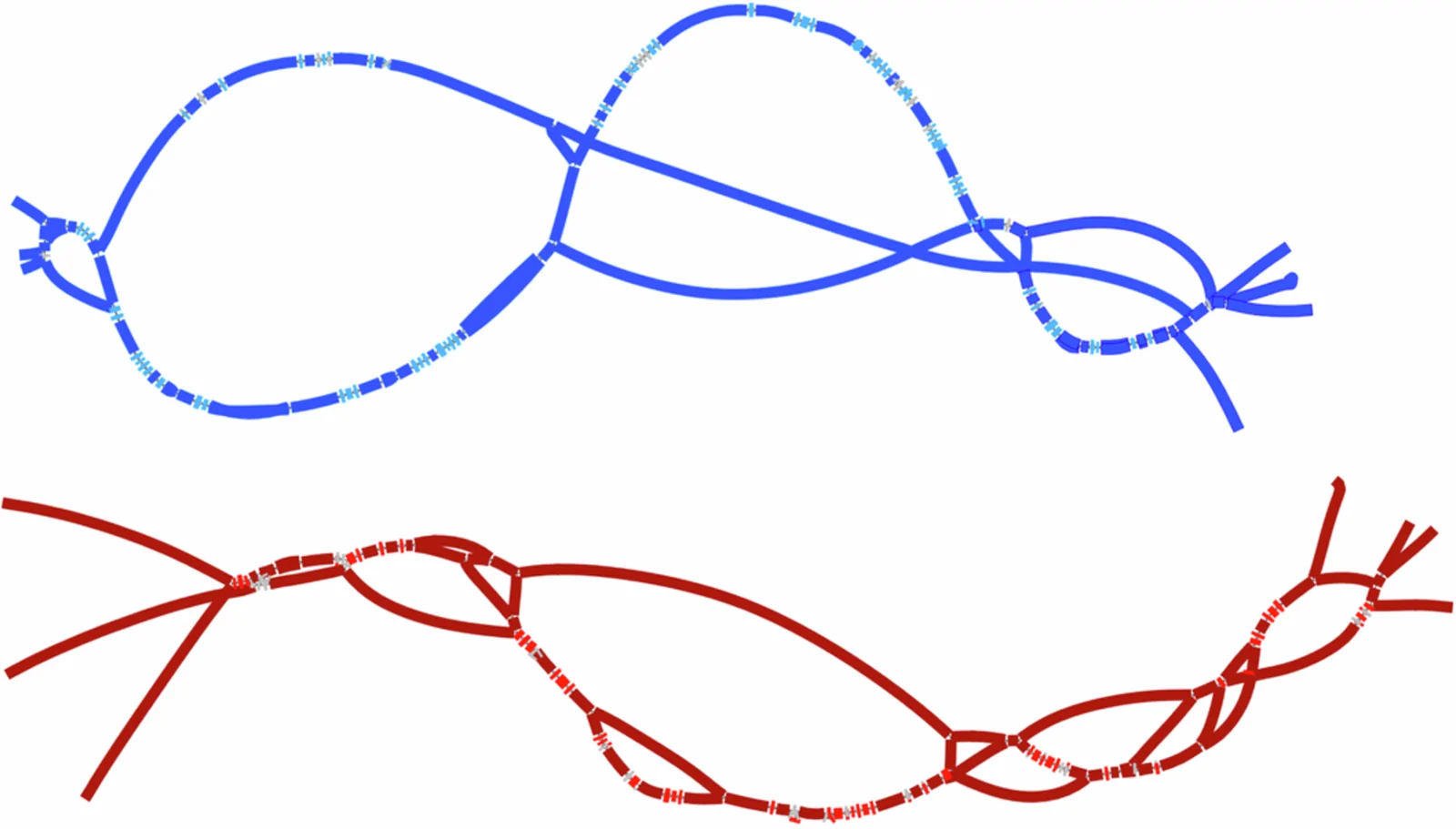
A real example from the wax apple genome showing how the graph structure facilitates accurate chromosomal assignment of short unitigs lacking Hi-C signals. In the diagram, blue segments denote unitigs assigned to chromosome 3, and red segments denote unitigs assigned to chromosome 5. Light-colored segments represent short unitigs without Hi-C support, which can be correctly assigned based on their topological connections to the confidently classified long unitigs.

**Extended Data Fig. 2:**
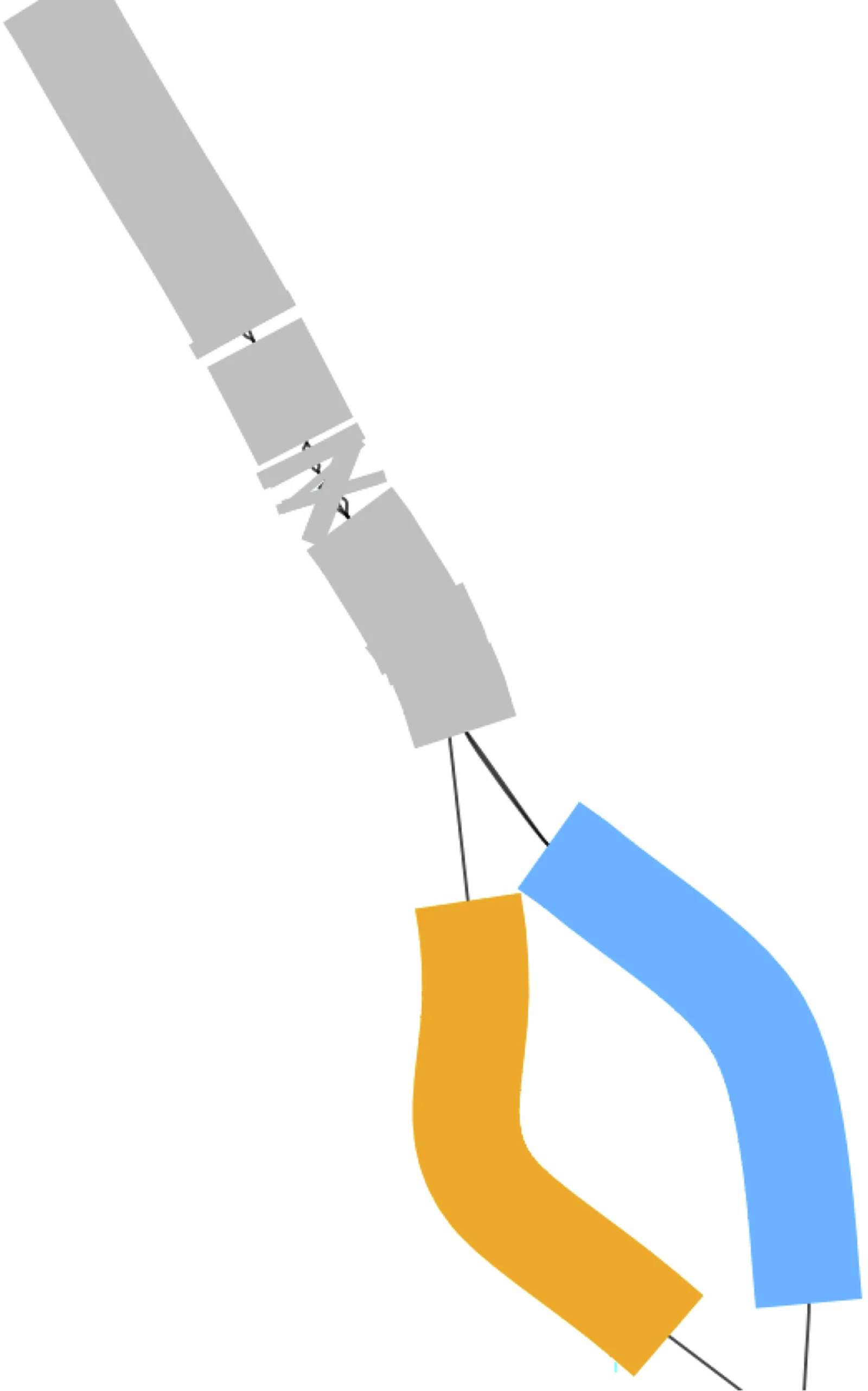
A real example from the simulated genome showing how the graph reveals interconnectivity between collapsed unitigs and multiple haplotypes. In the assembly graph, gray regions correspond to collapsed genomic regions, whereas distinct haplotypes are represented by blue (haplotype 1) and orange (haplotype 2) segments.

**Extended Data Fig. 3:**
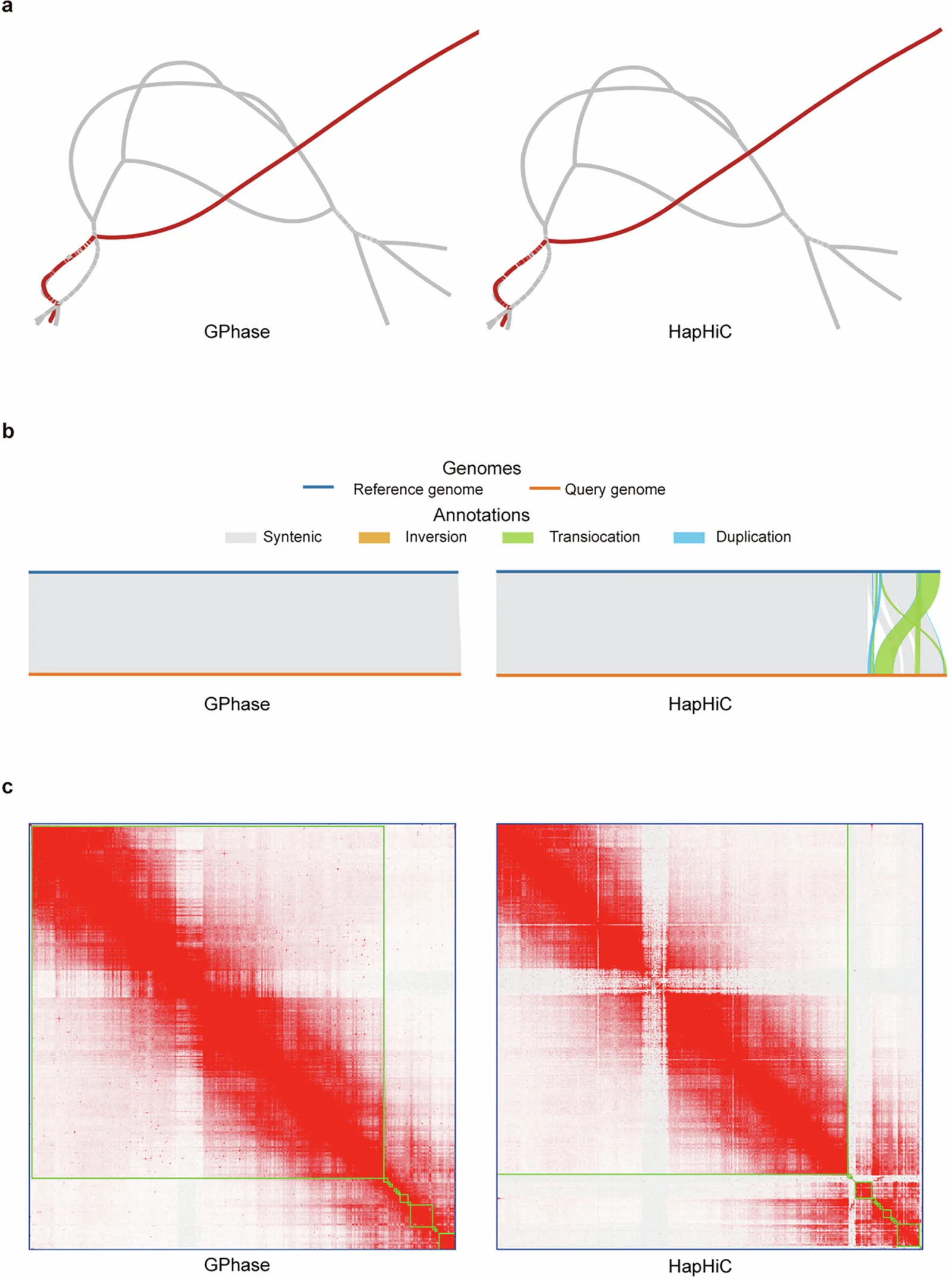
A real example from the rose genome illustrating how the graph structure facilitates genome scaffolding. **a**. Visualization of nearly identical unitig sets produced by GPhase and HapHiC using Bandage. **b**. Synteny plot comparing the GPhase and HapHiC assemblies with the ground-truth genome. **c**. Hi-C contact maps of the GPhase and HapHiC assemblies.

**Extended Data Fig. 4:**
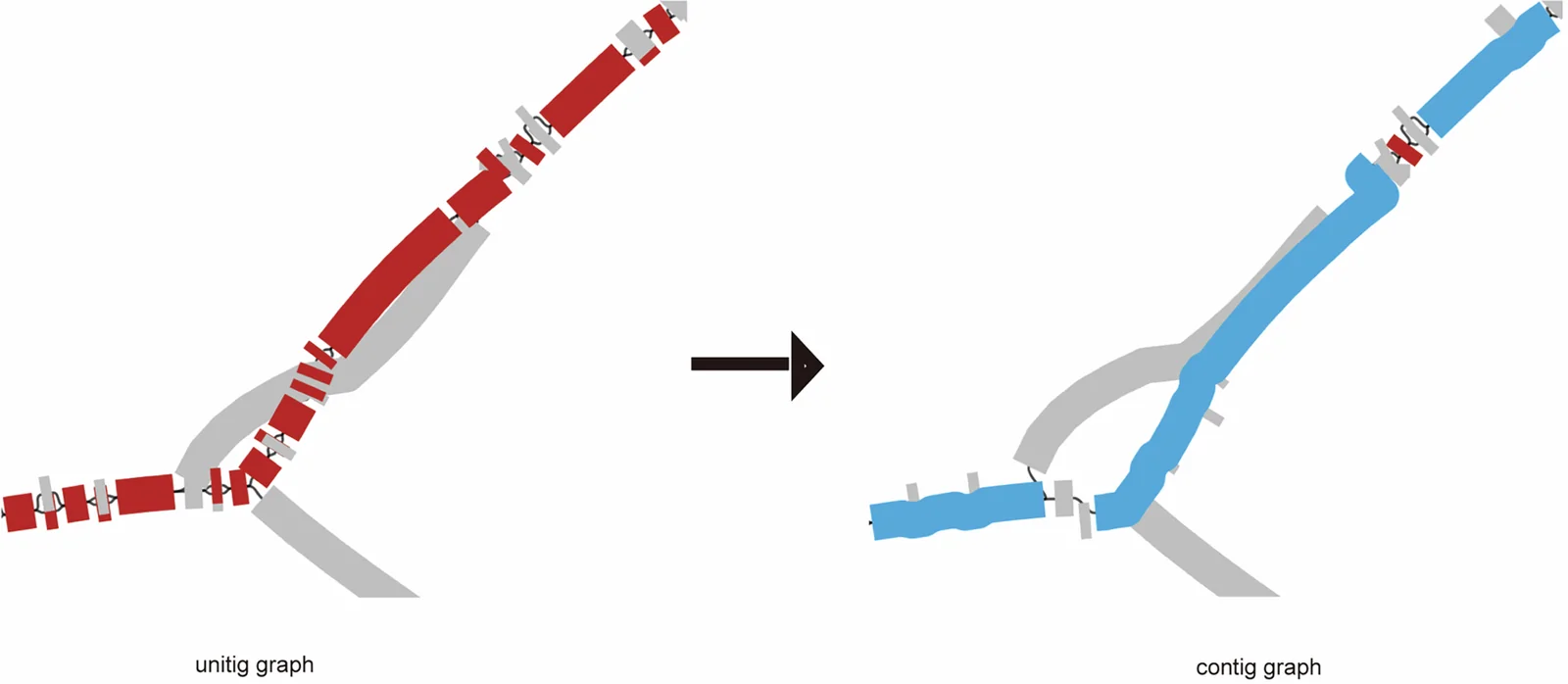
A real example from the simulated genome showing how the graph facilitates the direct assembly of unitigs into contigs, enhancing the contiguity of gap-free sequences. In the left panel, red segments denote the initial unitigs, which were extended into longer contigs (blue blocks) via path-guided linkage within the assembly graph.

To overcome these limitations, in this paper, we propose a new method that integrates a simplified assembly graph—produced during the unitig assembly step—to assist in the 3C-based phasing and scaffolding process. The assembly graph, which captures overlap relationships between long reads, provides complementary connectivity that mitigates 3C weaknesses. For example, short unitigs lacking 3C signals can be placed correctly using graph connections (Extended Data Fig. 1). Collapsed unitigs can be detected by their multiple haplotype links in the graph, helping prevent mis-phasing (Extended Data Fig. 2). Moreover, the graph partially indicates the relative ordering of unitigs along chromosomes, thereby improving scaffolding accuracy (Extended Data Fig. 3). Importantly, it also enables the direct assembly of unitigs into contigs (Extended Data Fig. 4), enhancing the contiguity of gap-free sequences—a capability absent from existing polyploid scaffolding tools. Mainstream unitig assemblers such as hifiasm, Flye and Shasta^30^ output assembly graphs by default, making this approach broadly applicable. It is worth noting that although some diploid assembly tools, such as hifiasm, also leverage assembly graphs to assist 3C-based phasing, they do not perform well when directly extended to polyploid genomes, highlighting the intrinsic challenges of this problem.

We implemented this strategy in a haplotype-resolved scaffolding algorithm, GPhase, specifically designed for polyploid genomes. GPhase integrates 3C signals (for example, Hi-C, Pore-C and Omni-C) with the assembly graph, jointly leveraging both data types to enable the construction of accurate and complete chromosome-level assemblies. Through a series of algorithmic innovations, GPhase effectively unifies these two complementary sources of information. Benchmarking on 15 complex polyploid genomes using Hi-C, Pore-C and Omni-C data demonstrated that GPhase substantially outperforms existing tools, reducing the sequence missing rate (BUSCO-based assessment) by up to several tens-fold and the sequence error rate (*k*-mer-based evaluation) by up to an order of magnitude, while also improving the contiguity (N50) of gap-free sequences by several-fold. These datasets included two semi-simulated polyploid genomes with high-quality ground-truth assemblies, generated in this study by merging four and three homologous haploid rice genomes, respectively, to enable rigorous performance evaluation; four purely in-silico-simulated autopolyploid genomes with varying degrees of sequence similarity; and five real polyploid genomes, including potato, wax apple and rose. GPhase was also independently evaluated on an additional four polyploid genomes to further assess its effectiveness.

## Results

### GPhase algorithm

**Fig. 1:**
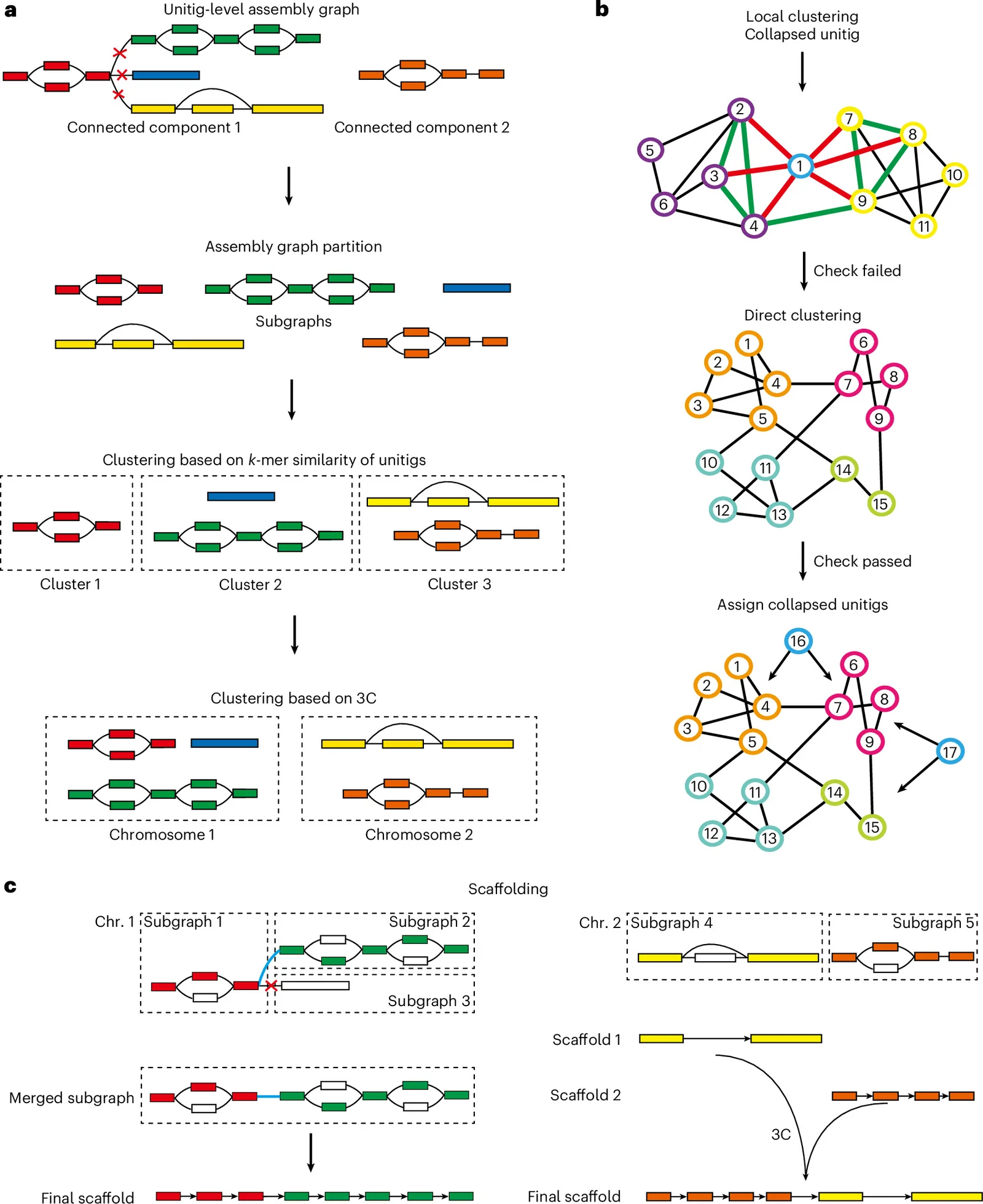
Comprehensive overview of the GPhase algorithm pipeline. The algorithm reconstructs phased chromosome-level assemblies through three steps. **a**, Chromosome partitioning (Step 1). Starting from a unitig-level assembly graph (top), unitigs (solid rectangles) connected by overlap edges (black lines) are partitioned by cutting specific edges (red Xs). Subgraphs are grouped into clusters on the basis of *k*-mer similarity (dashed boxes) and then assigned to chromosome bins (for example, Chr. 1 and Chr. 2) using global 3C interaction signals. **b**, Haplotype phasing (Step 2). Unitigs are separated into haplotypes. Local clustering uses a collapsed unitig (node 1, blue outline) to guide phasing of its neighbours. Red edges connect the collapsed unitig to first-order neighbours; green edges connect among these neighbours. Clustering is performed on the green-edge network, and the results are validated. If passed, the algorithm assigns collapsed unitigs; otherwise, a direct clustering strategy groups nodes on the basis of global 3C signals (solid black edges) among non-collapsed unitigs. Finally, collapsed unitigs are assigned to their haplotype clusters. **c**, Scaffolding (Step 3). Within each chromosome bin (for example, Chr. 1), subgraphs are linearized using local graph topology. Valid topological connections (solid blue lines) are selected; alternative paths (red X) are discarded. Blank rectangles represent unitigs not belonging to the current haplotype. For disjointed sequences (for example, scaffolds 1 and 2 in Chr. 2), global 3C linkage information (curved black arrows) determines their correct order and orientation, yielding final chromosome-level scaffolds.

**Fig. 2:**
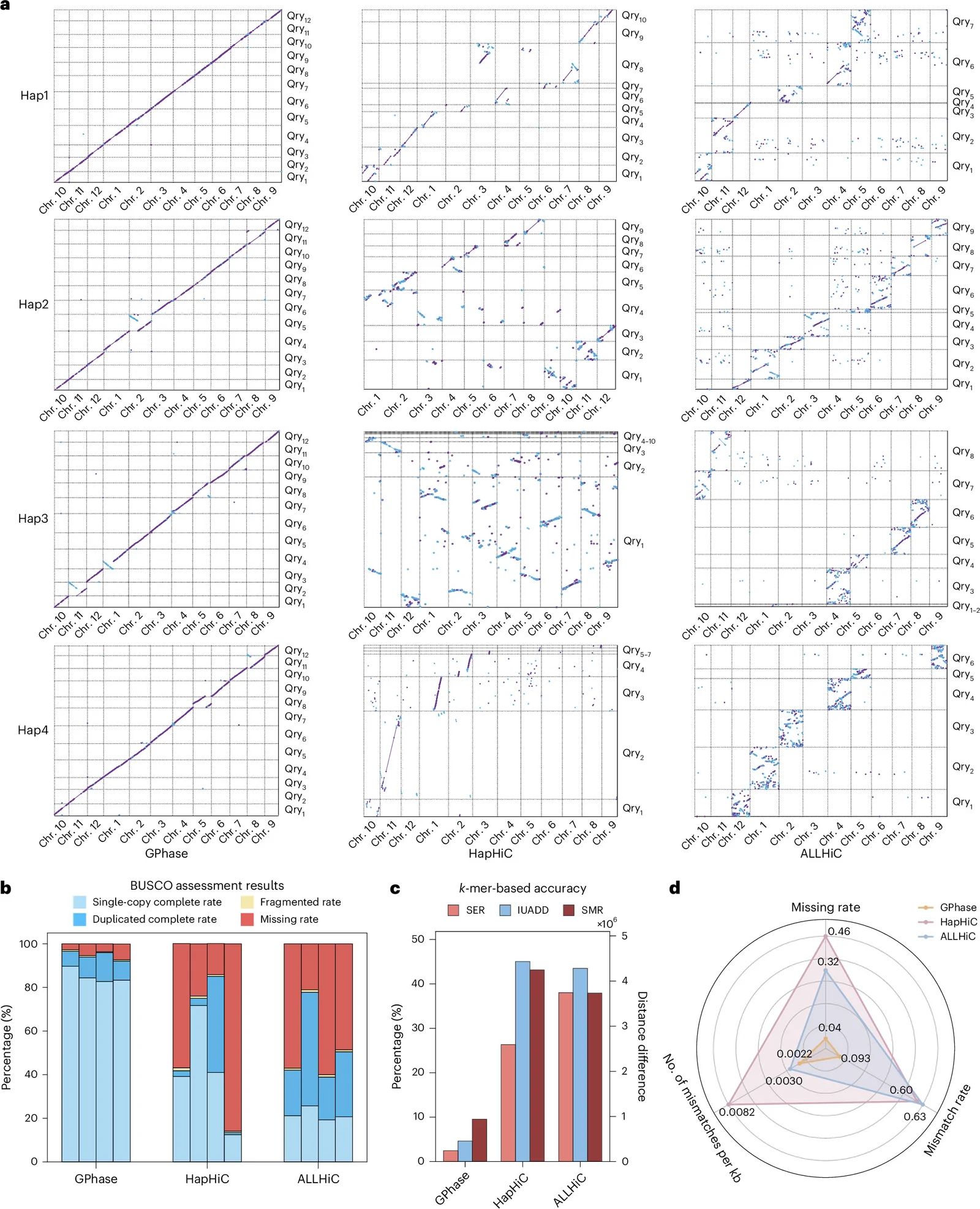
Performance comparison of GPhase, HapHiC and ALLHiC on the semi-simulated tetraploid genome. **a**, Sequence-alignment-based synteny plots of chromosome-level genome assemblies generated by the three tools. The horizontal axes show reference chromosomes; the vertical axes show query haplotypes from each assembly (Qry). Purple and blue dots indicate alignments in the forward and reverse orientations, respectively. **b**, BUSCO completeness scores of the genome assemblies; each bar represents one haplotype. **c**, *k*-mer-based assessment scores: sequence error rate (SER), sequence missing rate (SMR) and inter-unitig average distance difference (IUADD), with lower values indicating higher accuracy. **d**, Sequence-alignment-based assessment scores of the assemblies: missing rate, mismatch rate and number of mismatches per kilobase, with lower values indicating higher accuracy.

The input to the GPhase algorithm includes a unitig-level assembly graph generated by unitig assemblers, where nodes represent unitig sequences and edges represent the connections between unitigs, as well as the alignments of 3C reads to the unitigs. The outputs consist of chromosome-level haplotype sequences and contigs assembled from unitigs. GPhase operates in a preprocessing step, which performs 3C data normalization and unification (Methods), followed by three main steps: (1) partitioning the unitig graph into subgraphs, each ideally corresponding to a chromosome (Fig. 1a); (2) clustering unitigs in each chromosome-specific subgraph into distinct haplotypes (phasing) (Fig. 1b); and (3) assembling unitigs into contigs and performing chromosome-level scaffolding for each haplotype (Fig. 1c). The overall workflow of the algorithm is illustrated in Fig. 1.

In the second step (phasing), a key challenge is to prevent unitigs from different haplotypes from being incorrectly clustered together due to strong 3C signals they both share with the same collapsed unitigs—an issue discussed in the introduction. To address this problem, we propose an algorithm that first clusters the set of non-collapsed unitigs directly connected to each collapsed unitig via strong 3C signals. This step aims to minimize the likelihood of unitigs from different haplotypes being grouped together due to shared collapsed regions. On the basis of the results of the first-round clustering, we then perform a second-round clustering on all non-collapsed unitigs across the chromosome to form haplotype-scale groups. Similar to the initial chromosome partitioning step, an adaptive resolution tuning strategy is employed to ensure that the number of resulting clusters matches the expected ploidy level of the genome. This method usually works well for highly collapsed chromosomes, but in cases with fewer collapsed unitigs, the second round of clustering may fail due to insufficient information provided by the first round. To address this issue, GPhase employs a validation strategy that evaluates the correctness of each cluster by examining the degree of sequence homology within the cluster. The rationale is that a correct cluster should contain sequences from only one haplotype and therefore exhibit low homology. Sequence homology is computed by integrating both sequence similarity and graph structure, as bubble structures in the graph represent reliable homologous regions. If the validation indicates low clustering accuracy, GPhase discards the current clustering results and instead adopts a more general approach by directly clustering all non-collapsed unitigs using 3C signals. After successfully clustering the non-collapsed unitigs into their respective haplotypes, GPhase assigns the collapsed unitigs to haplotypes according to the strength of 3C signals and their sequence homology to the non-collapsed unitigs within each haplotype. The number of haplotypes to which each collapsed unitig should be assigned is estimated from its read coverage.

In the third step, GPhase first uses the graph structure to assemble the unitigs within each haplotype into longer contigs and then anchors these contigs to chromosome-scale sequences using 3C data. To recover unitigs that may have been lost during the earlier chromosome-level and haplotype-level clustering steps, GPhase employs a rescue strategy. The rescue strategy calculates the pairwise shortest paths between unitigs within each haplotype using the whole-genome assembly graph and includes the intermediate unitigs on these shortest paths into the corresponding haplotype. With the augmented set of unitigs, the algorithm identifies all subgraphs associated with the haplotype (obtained during the first step of GPhase through graph partitioning) and prunes nodes that do not belong to this haplotype. Subgraphs that are connected by unambiguous edges are merged into larger ones, and a topological sort is then applied to determine the order of unitigs within each subgraph, producing contigs. The resulting contigs are then scaffolded in two rounds using 3C data, first with YaHS^31^ and then with the scaffolding module of HapHiC, ultimately producing a chromosome-level assembly.

### Benchmarking on simulated polyploid genomes with Hi-C data

In this section, we evaluate the performance of GPhase by comparing it with two state-of-the-art haplotype-resolved scaffolding algorithms, ALLHiC and HapHiC, using Hi-C data. To assess the performance of GPhase on genomes with high-quality ground-truth assemblies, we constructed two semi-simulated tetraploid and triploid genome datasets by merging sequencing data from four and three real cultivated rice (*Oryza sativa*, AA genome) accessions, respectively, with each accession representing one haplotype of the polyploid genomes. Correspondingly, the sequencing data—including HiFi, ONT ultra-long and Hi-C reads—from each rice accession were combined to generate the sequencing datasets for the tetraploid and triploid genomes. The tetraploid genome was constructed from two japonica (Nipponbare and YF47) and two indica (9311 and FL478) accessions, resulting in a higher level of heterozygosity (1.87%), whereas the triploid genome was constructed from three japonica (Nipponbare, YF47 and BD8) accessions and therefore exhibits lower heterozygosity (0.70%). Among the five related rice genomes used in this study (the evolutionary relationships are shown in Supplementary Fig. 1), the raw sequencing data and ground-truth haplotype assemblies of FL478 and YF47 were generated as part of this work, while those of 9311^32^, Nipponbare^33^ and BD8^34^ were generated either in our previous studies or by other researchers.

For both semi-simulated genomes, HiFi and ONT ultra-long data were assembled into unitigs using hifiasm, which also produced unitig-level assembly graphs. On the basis of these assemblies, the unitig collapse rates for the tetraploid and triploid genomes were 22.7% and 48.2%, respectively, indicating that more than one fifth of the tetraploid genome and nearly half of the triploid genome remained in a collapsed state. GPhase, ALLHiC and HapHiC were then applied to perform haplotype-resolved scaffolding of the unitigs on both genomes using Hi-C data, either with or without the assistance of the assembly graph.

**Extended Data Fig. 5:**
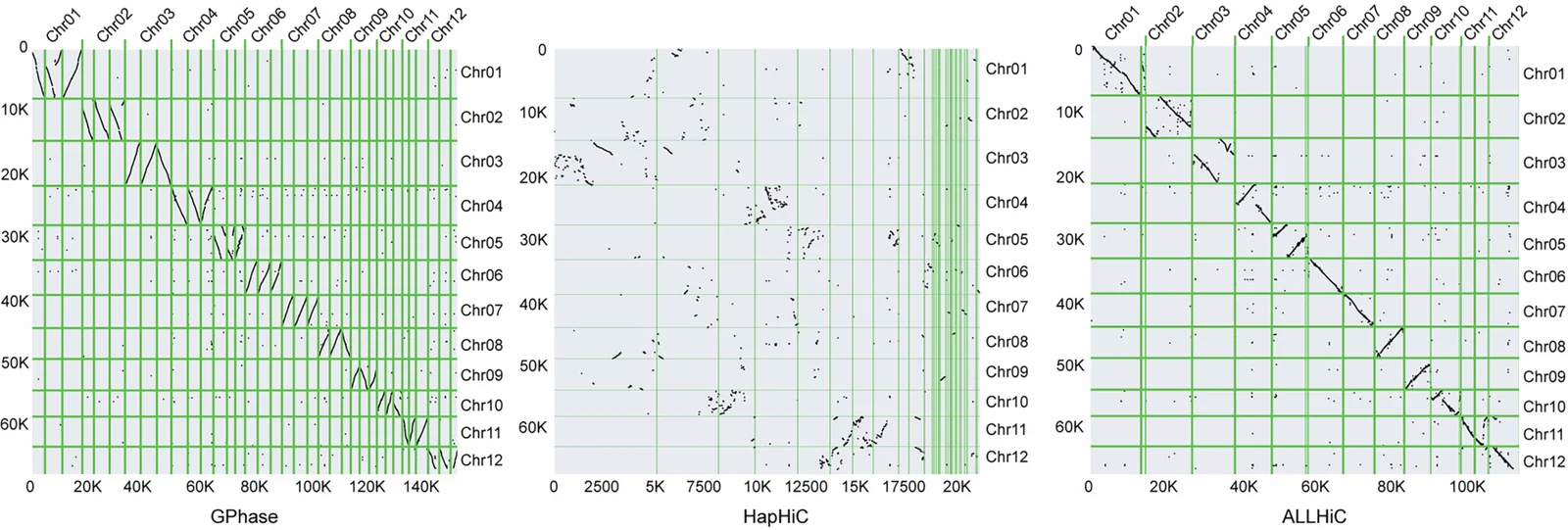
Gene-based synteny plots of the semi-simulated triploid genome assemblies generated by the three tools. The horizontal and vertical axes represent the assembly and the reference genome, respectively.

Both sequence-alignment-based and gene-based synteny plots show that the GPhase assembly clearly outperforms those generated by the other two methods in terms of overall accuracy (Fig. 2a, Supplementary Figs. 2 and 3 and Extended Data Fig. 5). Although minor phasing errors and limited sequence losses are observed, GPhase successfully resolves four and three haplotypes of comparable length for each chromosome in the tetraploid and triploid genomes, respectively, and these haplotype sequences show high concordance with the ground truth. These minor issues can be readily corrected through limited manual curation. In contrast, HapHiC fails to produce valid assemblies for any chromosome in either genome. The ALLHiC assembly performs better than HapHiC on the tetraploid genome; however, several chromosomes are partitioned into highly unbalanced haplotype groups, and the corresponding synteny plots exhibit highly fragmented alignments between the assembly and the ground truth, indicating suboptimal assembly quality. On the triploid genome, ALLHiC generates only a single haplotype sequence for most chromosomes, suggesting a failure to correctly resolve haplotypes. BUSCO^35^ evaluation showed that all haplotype sets in the GPhase assemblies exhibited consistently high completeness, whereas the assemblies generated by the other two tools suffered from substantial sequence loss (Fig. 2b and Supplementary Fig. 4). Similarly, the Hi-C heat maps highlight the clear advantage of GPhase over the other methods (Supplementary Figs. 5 and 6). Furthermore, evaluation against the ground truth using unique *k*-mer-based metrics demonstrated that the GPhase assembly achieved substantially higher phasing accuracy (Fig. 2c and Supplementary Fig. 7), as evidenced by the lowest sequence error rate and sequence missing rate, as well as markedly superior scaffolding accuracy, reflected by the lowest inter-unitig average distance difference, a metric that captures sequence correctness within each haplotype. Consistently, evaluation using sequence-alignment-based metrics further showed that GPhase assemblies exhibit significantly higher completeness and correctness, as indicated by the lowest missing rate, mismatch rate and number of mismatches per kilobase (Fig. 2d and Supplementary Fig. 8). Finally, the *k*-mer distribution plots revealed a high degree of consistency between the GPhase assemblies and the ground truths (Supplementary Fig. 9). GPhase assembles the unitigs into contigs, improving the contiguity of gap-free sequences (N50) from 0.66 Mb to 1.35 Mb for the tetraploid genome and from 0.23 Mb to 0.64 Mb for the triploid genome, a capability not achieved by HapHiC or ALLHiC.

**Fig. 3:**
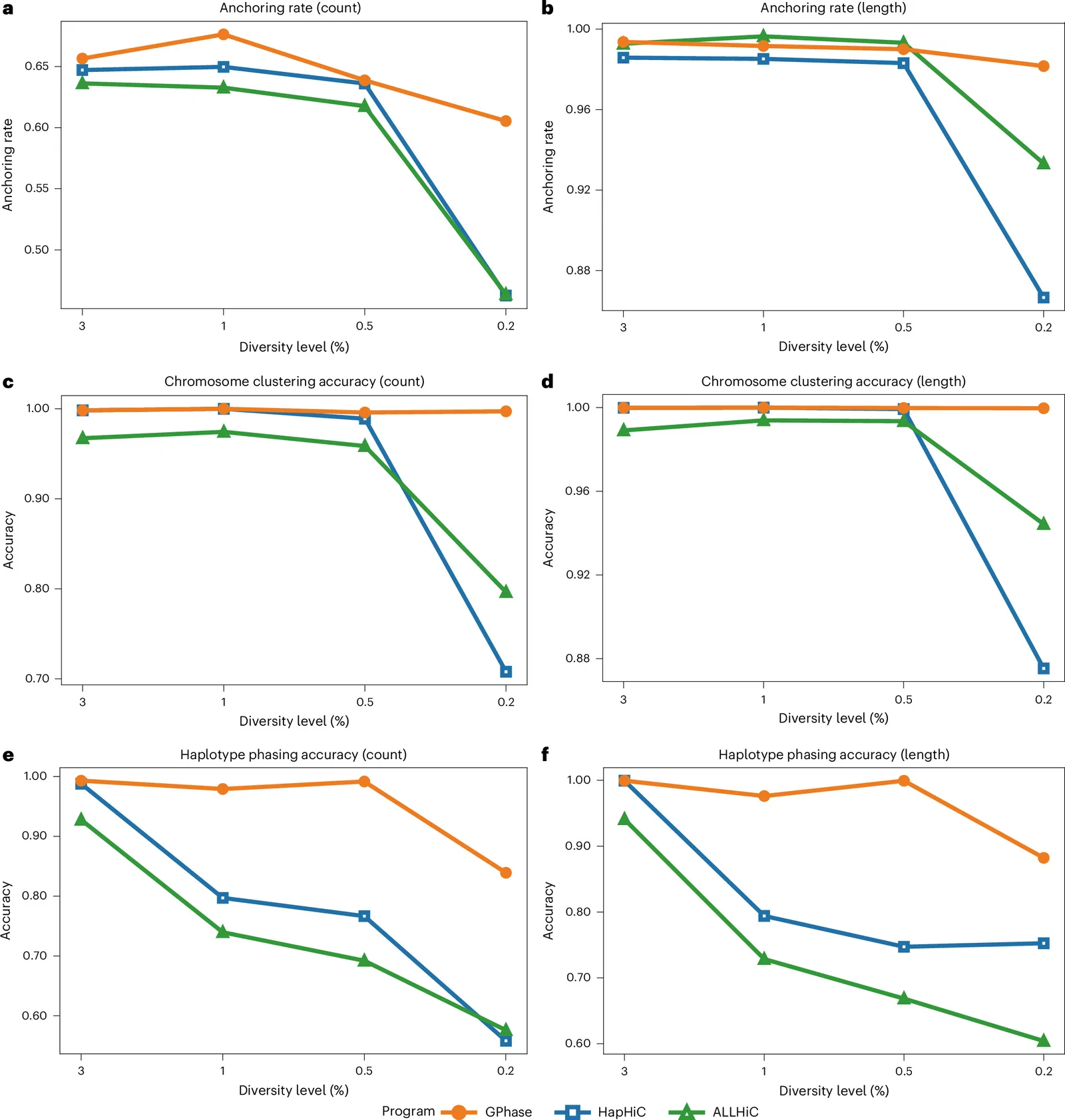
Performance comparison of GPhase with state-of-the-art tools on fully simulated polyploid genomes with varying levels of divergence using Hi-C data. **a**, Anchoring rate (count), defined as the proportion of unitigs incorporated into chromosome-level assemblies. **b**, Anchoring rate (length), defined as the proportion of unitig bases incorporated into chromosome-level assemblies. **c**, Chromosome clustering accuracy (count), measured as the proportion of unitigs assigned to the correct chromosome. **d**, Chromosome clustering accuracy (length), measured as the proportion of total unitig length assigned to the correct chromosome. **e**, Haplotype phasing accuracy (count), defined as the proportion of unitigs assigned to the correct haplotype. **f**, Haplotype phasing accuracy (length), defined as the proportion of total unitig length assigned to the correct haplotype.

As a further step, we compared the performance of the three algorithms on fully simulated polyploid genomes with varying levels of sequence divergence. Four tetraploid genomes were constructed by merging the Nipponbare T2T genome with three simulated haplotype rice genomes, which were generated by introducing sequence variations into the Nipponbare genome. The sequence divergence between the Nipponbare genome and the simulated haplotype genomes was set to 3%, 1%, 0.5% and 0.2% for the four tetraploid genomes, respectively. The raw sequencing reads, including HiFi and Hi-C data, were generated using simulation software. Unitigs and ground-truth genomes were produced following the same procedures as those used in the semi-simulated genome experiments. Assembly completeness and correctness were evaluated using unitig-level metrics, including anchoring rate (Fig. 3a,b), chromosome clustering accuracy (Fig. 3c,d) and haplotype phasing accuracy (Fig. 3e,f), following established practices in previous studies of polyploid genome assembly. Overall, the three tools showed comparable performance on genomes with higher sequence divergence. However, for low-divergence genomes, GPhase consistently outperformed HapHiC and ALLHiC across most metrics, demonstrating its superior ability to resolve more challenging polyploid genome assemblies.

Finally, we validated the performance of each individual step of GPhase using the aforementioned semi-simulated autotetraploid genome. To this end, we constructed ground-truth datasets corresponding to the intermediate outputs of the GPhase pipeline. Each of the three steps of GPhase was then executed and evaluated independently, using the original GPhase inputs and/or the corresponding ground-truth intermediate results as inputs. The detailed experimental procedures and results are provided in Supplementary Note 1, Supplementary Table 1 and Supplementary Figs. 10–13, demonstrating the high performance of each step of the GPhase algorithm.

### Benchmarking on real polyploid genomes with Hi-C, Pore-C and Omni-C data

We further compared the performance of GPhase against state-of-the-art methods using diverse 3C datasets on three real polyploid genomes, including a tetraploid rose genome (*Rosa hybrida* ‘Samantha’^36^) and two autotetraploid potato genomes (*Solanum tuberosum* ‘Otava’^12^ and ‘Katahdin’^37^), all of which have relatively high-quality ground-truth assemblies constructed by integrating multiple types of data followed by extensive manual curation.

Because both Hi-C and Pore-C data are available for the rose genome, we performed a comprehensive and systematic comparison across multiple methods. For Hi-C data, we evaluated GPhase, HapHiC and ALLHiC, as well as a combined pipeline consisting of hifiasm-based phasing followed by YaHS-based scaffolding. For Pore-C data, we compared GPhase with HapHiC and C-Phasing^38^, the only existing tool specifically designed for Pore-C data. The unitigs and the unitig graph were generated by hifiasm using HiFi reads and ONT ultra-long reads, and the collapse rate was 30.4%.

**Fig. 4:**
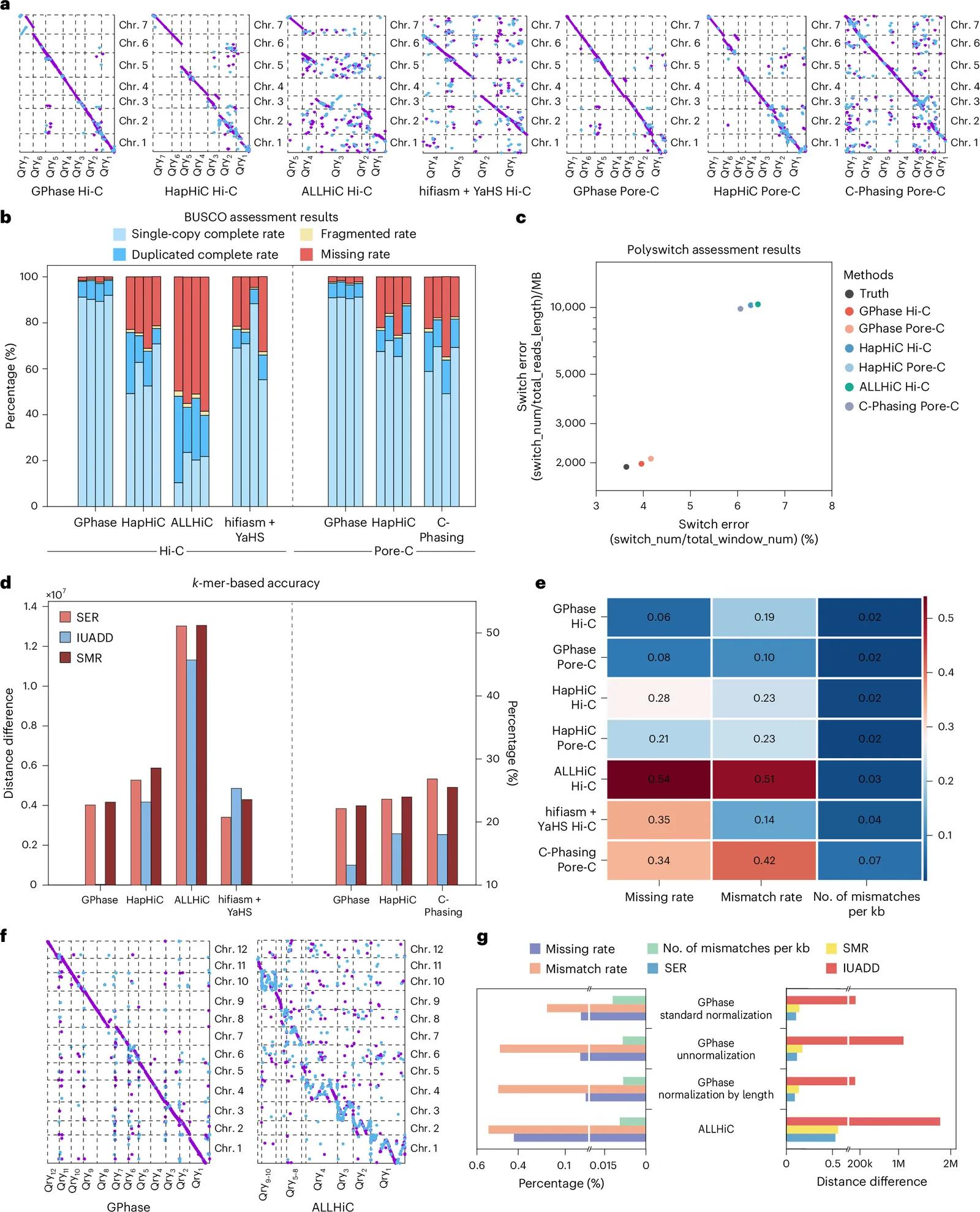
Performance comparison of GPhase with state-of-the-art tools on the rose (*R. hybrida* ‘Samantha’) and potato (*S. tuberosum* ‘Katahdin’) genomes. **a**, Synteny plots of chromosome-level assemblies for one rose haplotype; purple and blue dots indicate alignments in the forward and reverse orientations, respectively. Full plots are shown in Supplementary Fig. 15 and Extended Data Fig. 6. **b**, BUSCO completeness scores of the rose genome assemblies; each bar represents one haplotype. **c**, Switch-error numbers and rates of the rose genome assemblies. ‘Truth’ represents the ground-truth genome. **d**, *k*-mer-based assessment scores of the rose genome assemblies: SER, SMR and IUADD, with lower values indicating higher accuracy. **e**, Sequence-alignment-based assessment scores of the rose genome assemblies: missing rate, mismatch rate and number of mismatches per kilobase. **f**, Synteny plots for one potato haplotype using Omni‑C data; full plots are shown in Supplementary Fig. 24. **g**, *k*-mer-based and sequence-alignment-based assessment scores of the potato genome.

**Extended Data Fig. 6:**
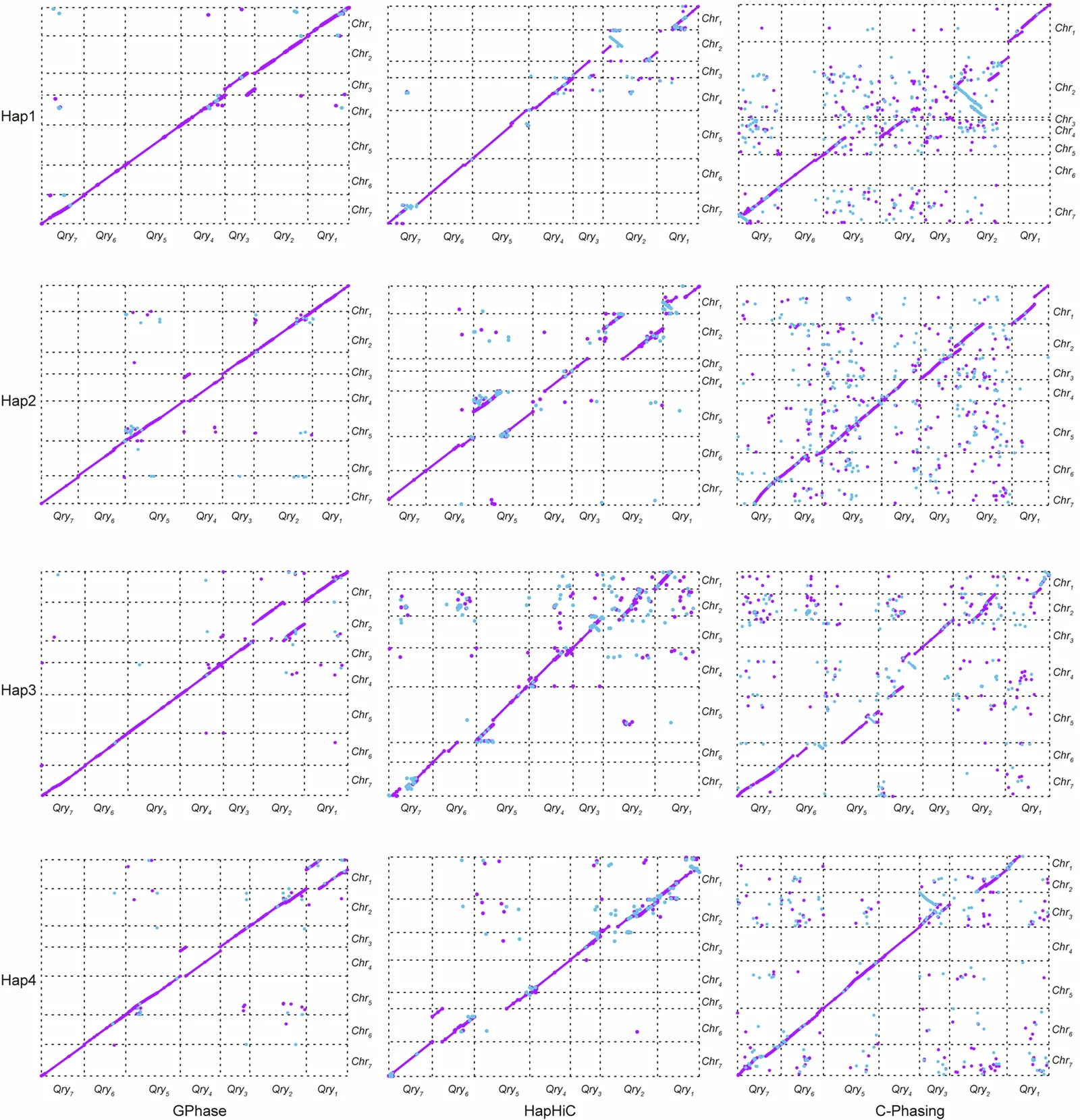
Sequence-alignment-based synteny plots of the rose (*R. hybrida* ‘Samantha’) genome assemblies generated by the three tools using Pore-C data. Each row corresponds to a specific haplotype (Hap1 to Hap4), and each column represents a different phasing method: GPhase, HapHiC, and C-Phasing.

Sequence-alignment-based and gene-based synteny analyses consistently showed that GPhase outperformed other tools in both the Hi-C and Pore-C experiments (Fig. 4a, Supplementary Figs. 14–16 and Extended Data Fig. 6). For Hi-C data, both GPhase and HapHiC substantially outperformed ALLHiC. The ALLHiC assembly exhibited highly unbalanced haplotype sizes and produced fewer haplotypes per chromosome than expected given the ploidy level. Compared with GPhase, the HapHiC assembly also showed inconsistencies in haplotype number, including individual groups that contained multiple haplotypes, as revealed by gene-based synteny analysis (Supplementary Fig. 14). These issues in ALLHiC and HapHiC were further reflected at the level of individual haplotype assemblies, where pronounced sequence losses were observed in the sequence-alignment-based synteny plots (Fig. 4a and Supplementary Fig. 15). In contrast, the hifiasm + YaHS pipeline produced substantially more severe errors, generating far more haplotypes than the expected ploidy for many chromosomes (Supplementary Fig. 14), which resulted in a total assembly length (3.82 Gb) greatly exceeding the ground-truth genome size (1.97 Gb). For Pore-C data, although all three tools were able to generate generally reasonable assemblies, both HapHiC and C-Phasing showed conspicuous large missing regions, whereas such losses were not observed in the GPhase assembly, indicating the higher completeness achieved by GPhase (Fig. 4a, Supplementary Fig. 16 and Extended Data Fig. 6). When comparing Pore-C-based assemblies with those generated using Hi-C data, we found that methods that do not leverage the assembly graph indeed benefit more from Pore-C data, producing assemblies that are more complete and accurate than those obtained with Hi-C. This advantage is probably attributable to the longer read lengths of Pore-C data, which facilitate the resolution of short unitigs. In contrast, GPhase showed little difference between assemblies generated from Pore-C and Hi-C data and consistently outperformed the other methods under both conditions. This result demonstrates that the effective integration of assembly graph information can largely compensate for the limitations of Hi-C data, enabling better handling of short and collapsed unitigs, thereby diminishing the apparent advantage of Pore-C data.

**Extended Data Fig. 7:**
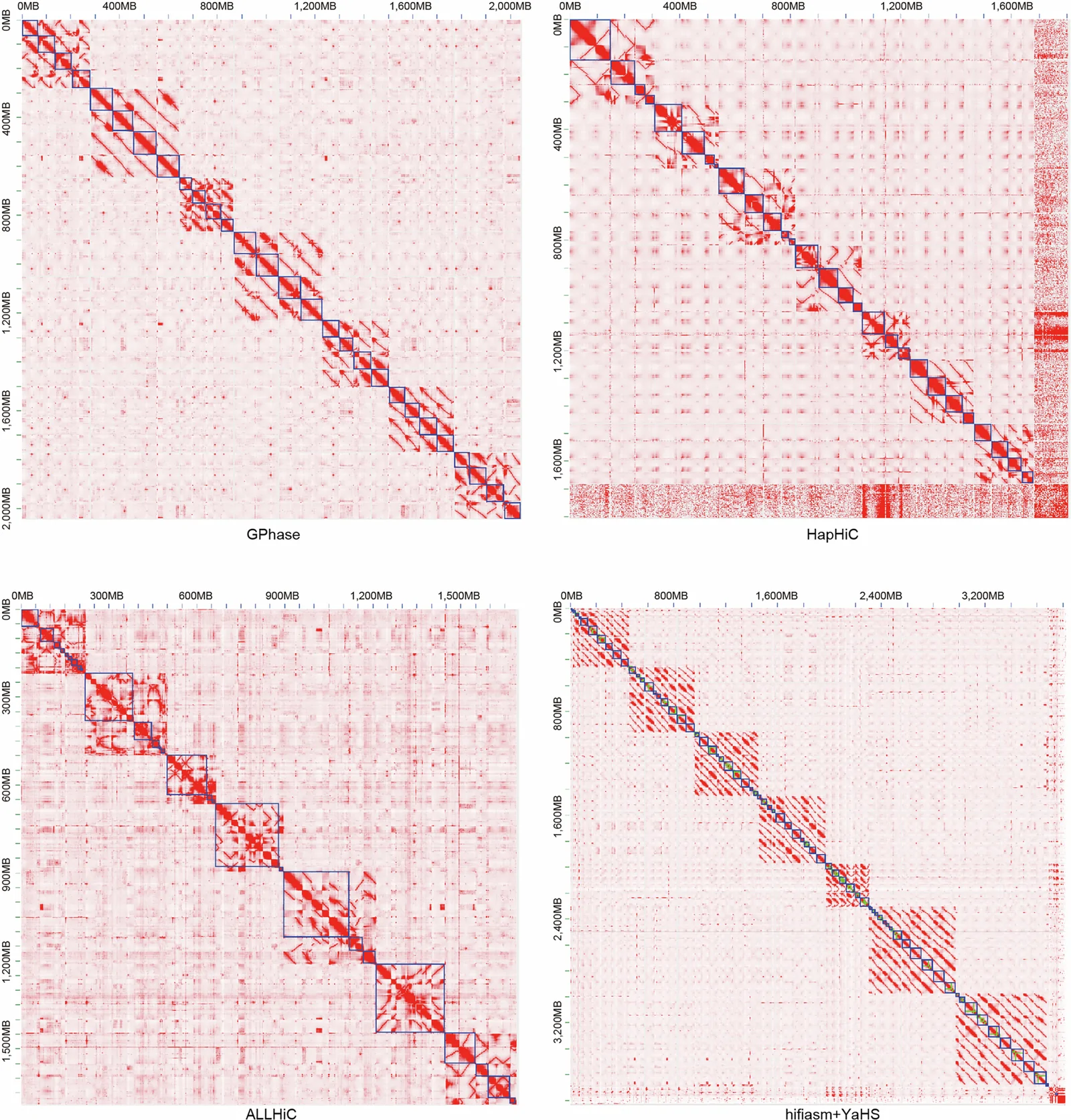
Hi-C contact maps of the rose (*R. hybrida* ‘Samantha’) genome assemblies generated by the four methods using Hi-C data. Blue boxes represent scaffolds.

The Hi-C and Pore-C contact maps further corroborate these findings, consistent with the synteny plots (Extended Data Fig. 7 and Supplementary Fig. 17). The BUSCO assessment showed that GPhase clearly outperformed the other two tools in terms of sequence completeness (Fig. 4b). The relatively high duplicated complete rates observed in some haplotype sets of HapHiC, ALLHiC and C-Phasing assemblies are attributable to the erroneous assignment of homologous sequences from multiple haplotypes into single haplotypes. By aligning long reads back to the assemblies, we further quantified phasing accuracy using the number of switch errors (Fig. 4c). Notably, the GPhase assemblies generated using either Hi-C or Pore-C data exhibited switch-error rates that were very close to those of the ground-truth assembly, whereas all other assemblies showed substantially higher switch-error rates and deviated markedly from the ground-truth benchmark. Similarly, both the unique *k*-mer-based assessment and sequence-alignment-based evaluation indicated that the GPhase assembly achieved superior sequence completeness, phasing accuracy and scaffolding precision (Fig. 4d,e). In the Hi-C experiment, for one haplotype of chromosome 4, where GPhase and HapHiC obtained nearly identical sets of unitigs during phasing, syntenic analysis and Hi-C contact maps consistently demonstrated that GPhase produced a completely correct assembly (Extended Data Fig. 3). In contrast, the HapHiC assembly exhibits misordered shorter unitigs due to erroneous scaffolding. This example highlights that incorporating graph structure in scaffolding not only prevents the loss of short unitigs but also ensures the accuracy of their associated sequences.

In the Hi-C experiment on the potato genome (*S. tuberosum* ‘Otava’), GPhase was compared with HapHiC and ALLHiC. The unitigs and unitig graph were generated by hifiasm using only HiFi reads, and Hi-C data were then used for scaffolding by the three tools included in the comparison. The collapse rate and heterozygosity were 41.8% and 4.3%, respectively. Due to the challenging nature of this dataset, all three algorithms produced assemblies of lower quality than other genomes. The gene-based synteny plots reveal that GPhase made a notable error by misassigning a small portion of chromosome 1 to chromosome 2 (Supplementary Fig. 18). Nevertheless, the overall assembly quality of GPhase remains substantially superior to that of HapHiC and ALLHiC (Supplementary Fig. 18). The HapHiC assembly contains a large erroneous group comprising sequences from nearly all chromosomes. In addition, many chromosomes exhibit incorrect haplotype phasing, with haplotype numbers deviating from the expected ploidy level and the majority of sequences assigned to a single dominant haplotype, as observed for chromosomes 5 and 8. ALLHiC fails to phase almost all chromosomes, assigning the vast majority of sequences from each chromosome to a single haplotype. These issues in HapHiC and ALLHiC are reflected in the sequence-alignment-based synteny plots as a high proportion of missing sequences across every haplotype (Supplementary Fig. 19). Hi-C contact maps further corroborate these observations, consistent with the synteny analyses (Supplementary Fig. 20). BUSCO assessment showed that although the GPhase assembly exhibited a higher sequence missing rate than observed for other datasets, its overall completeness was still markedly superior to that of the other two tools (Supplementary Fig. 21). In the unique *k*-mer-based assessment, GPhase achieved substantially lower values—by several-fold—in all sequence accuracy metrics, including sequence error rate, sequence missing rate and inter-unitig average distance difference, than HapHiC and ALLHiC (Supplementary Fig. 22). The alignment-based quantitative metrics yielded similar results (Supplementary Fig. 23).

In the Omni-C experiment on the potato genome (*S. tuberosum* ‘Katahdin’), GPhase was compared only with ALLHiC, as HapHiC was not applicable to this dataset. Similarly, unitigs and the corresponding unitig graph were generated using hifiasm solely on the basis of HiFi reads. Omni-C data were then used for scaffolding by both tools. Sequence-alignment-based synteny analysis showed that GPhase produced a high-quality haplotype-resolved assembly (Fig. 4f and Supplementary Fig. 24). GPhase successfully generated haplotypes of comparable sizes for all chromosomes, and the overall assembly quality was largely comparable to that achieved in the corresponding Hi-C experiments on another potato genome (*S. tuberosum* ‘Otava’). These results demonstrate the effectiveness of GPhase when applied to Omni-C data. In contrast, ALLHiC largely failed to phase most chromosomes, assigning nearly all sequences of each chromosome to a single group rather than resolving distinct haplotypes (Supplementary Figs. 25 and 26). BUSCO completeness assessments, unique *k*-mer–based evaluations and sequence-alignment-based analyses consistently supported these findings, collectively confirming the superior quality of the GPhase assemblies (Fig. 4g and Supplementary Fig. 27).

In addition, GPhase was independently evaluated on a broad range of polyploid datasets using either Hi-C or Pore-C data. These data comprise three genomes sequenced using Hi-C—the hexaploid persimmon (*Diospyros kaki* ‘Xiaoguotianshi’^39^), the tetraploid alfalfa (*Medicago sativa* ‘Xinjiangdaye’^40^) and the tetraploid Indian jujube (*Ziziphus mauritiana* ‘Xishuangyesheng’^41^)—and one genome sequenced using Pore-C, the tetraploid alfalfa (*M. sativa* ‘Zhongmu-4’^42^). Although assembly quality varied across genomes due to differences in genome complexity, GPhase consistently generated largely complete sequences for each haplotype of every chromosome in all tested genomes (Supplementary Figs. 28–31). After a moderate degree of manual curation, these assemblies were suitable for downstream fine-scale analyses. Collectively, these results demonstrate the broad effectiveness and robustness of GPhase across diverse polyploid genomes and 3C data types.

### Assembling wax apple and rose genomes with GPhase

**Fig. 5:**
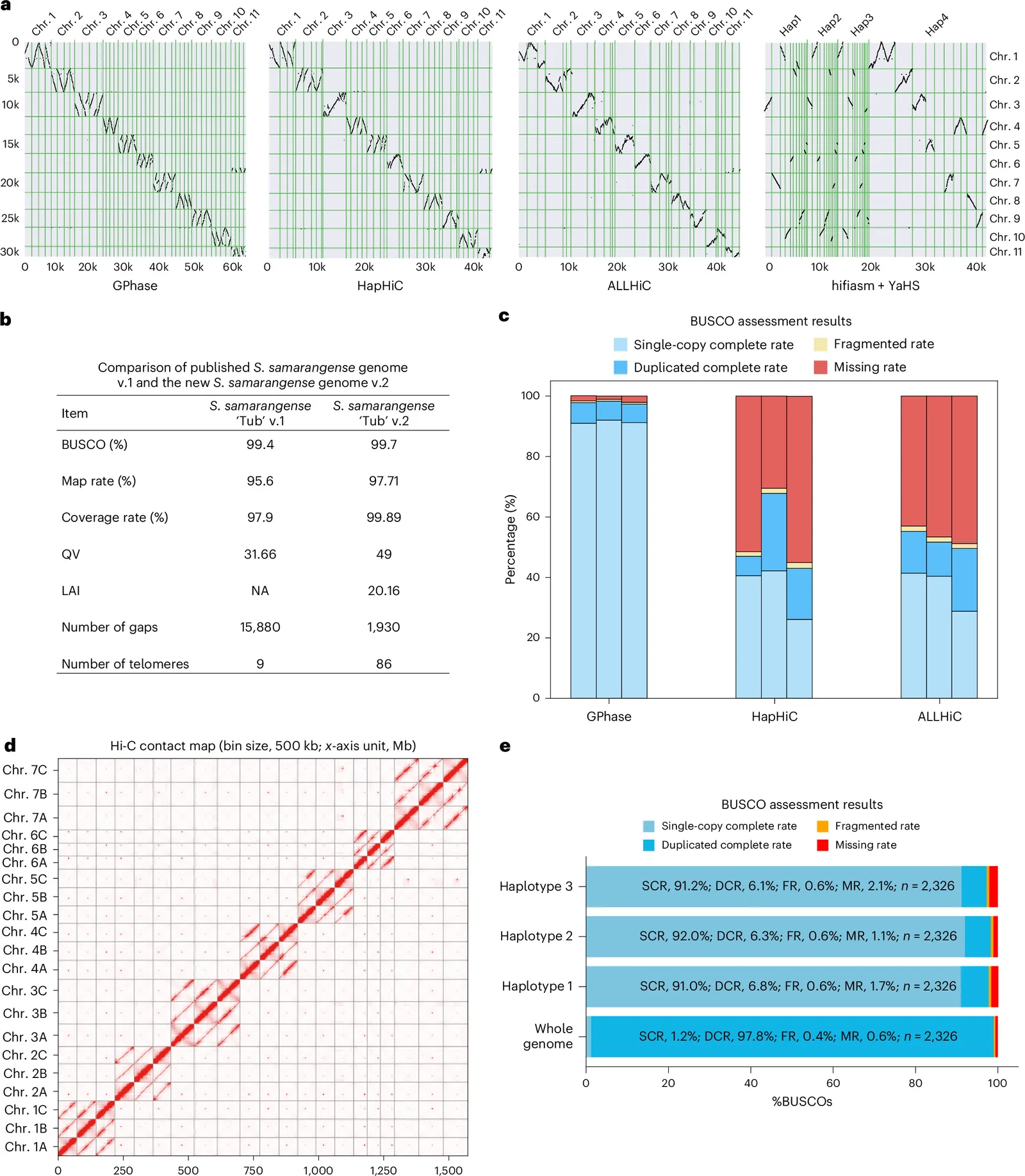
Experimental results on the autotetraploid wax apple genome (*S. samarangense* ‘Tub’) and rose (*R. chinensis* ‘Jin Ou Fan Lv’) genome. **a**, Gene-based synteny plots of chromosome-level wax apple genome assemblies generated by the four methods. The horizontal and vertical axes represent the assembly and the reference genome, respectively. **b**, Comparison between the final wax apple assembly produced in this study (*S. samarangense* v.2), obtained by manual curation of the GPhase assembly, and the previously published assembly (*S. samarangense* v.1). LAI, LTR assembly index; NA, the LAI evaluation software failed to run. **c**, BUSCO completeness scores of the rose genome assemblies generated by the three tools; each bar represents one haplotype. **d**, Hi-C contact maps of the final rose genome assembly obtained by manual curation of the GPhase assembly. **e**, BUSCO completeness scores of the final rose genome assembly obtained by manual curation of the GPhase assembly. SCR, single-copy complete rate; DCR, duplicated complete rate; FR, fragmented rate; MR, missing rate.

In this section, we apply a complete polyploid genome assembly workflow incorporating GPhase to assemble two representative case studies: a tetraploid wax apple genome (*Syzygium samarangense* ‘Tub’^43^) that was previously assembled and a triploid rose genome (*Rosa chinensis* ‘Jin Ou Fan Lv’) that has never been assembled before. To reassemble the wax apple genome, we first assembled HiFi reads^44^ and ONT reads into unitigs using hifiasm. The collapse rate and heterozygosity rate were 36.4% and 3.94%, respectively. We then performed haplotype-resolved scaffolding with Hi-C data using GPhase, HapHiC and ALLHiC. In addition, we applied the combined hifiasm + YaHS pipeline to the wax apple genome. Hifiasm generated phased contig groups, and YaHS was used to scaffold each group. Smudgeplot^45^ v.0.5.3 estimated the ploidy to be tetraploid (Supplementary Fig. 32), consistent with previous studies^43^, and this ploidy level was used as the parameter for hifiasm. The synteny plots show that, similar to the genomes discussed above, HapHiC, ALLHiC and hifiasm + YaHS suffer from unbalanced phasing and a number of haplotypes inconsistent with the expected ploidy—for example, chromosomes 2, 3, 6, 7, 9 and 11 in the case of HapHiC, and nearly all chromosomes for ALLHiC and hifiasm + YaHS (Fig. 5a). In contrast, GPhase produced more complete and size-balanced haplotypes for each chromosome. The BUSCO assessment further supports the idea that GPhase outperforms the other tools in terms of assembly completeness (Supplementary Fig. 33).

Furthermore, we performed manual curation on the assembly automatically generated by GPhase (Fig. 5b), including adjusting the order and orientation of sequences on the basis of Hi-C contact maps and synteny plots, and produced the final genome assembly of 1.62 Gb. Compared with the previous version, the new wax apple genome shows substantial improvements, with the assembly consensus quality value (QV) increasing from 31.66 to 49, the number of gaps reduced from 15,880 to 1,930 and the number of telomeres increasing from 9 to 86. Other quality metrics such as the BUSCO score, read mapping rate and read coverage rate also showed corresponding improvements.

**Extended Data Fig. 8:**
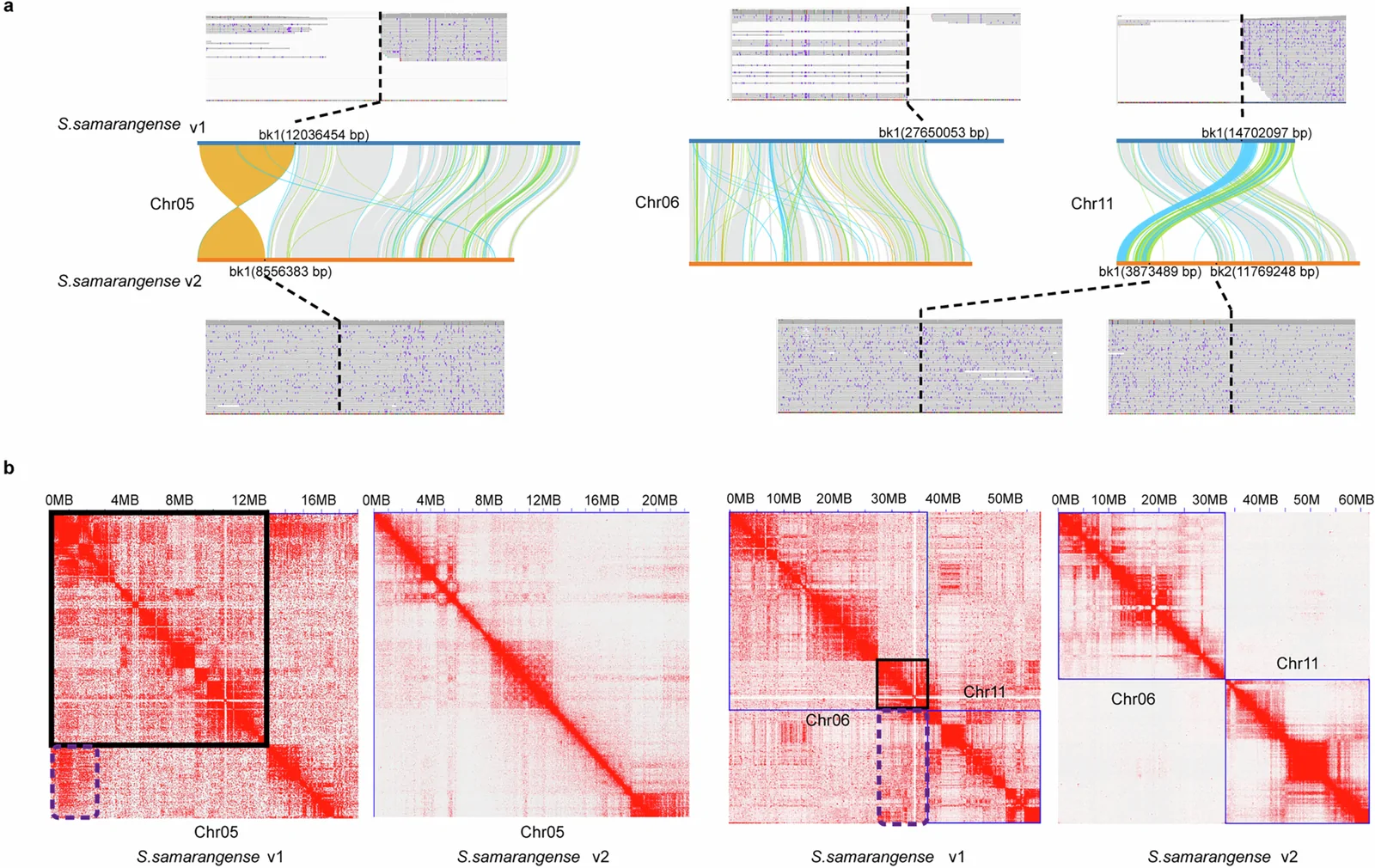
Validation of divergent regions between two versions of the wax apple (*S. samarangense* ‘Tub’) genome assembly. **a**. Synteny and long-read coverage validation for wax apple chromosomes 5, 6, and 11. The central panel shows the synteny plots of *S. samarangense* v2 (orange lines) to *S. samarangense* v1 (blue lines). The panels positioned above and below, connected by dashed lines, display coverage plots of long-read mappings visualized using IGV. **b**. Hi-C heatmaps comparing chromosomes 5, 6, and 11 between *S. samarangense* v1 and *S. samarangense* v2. Corrected regions are marked with black boxes; evidence for correction indicated by purple dotted boxes.

**Extended Data Fig. 9:**
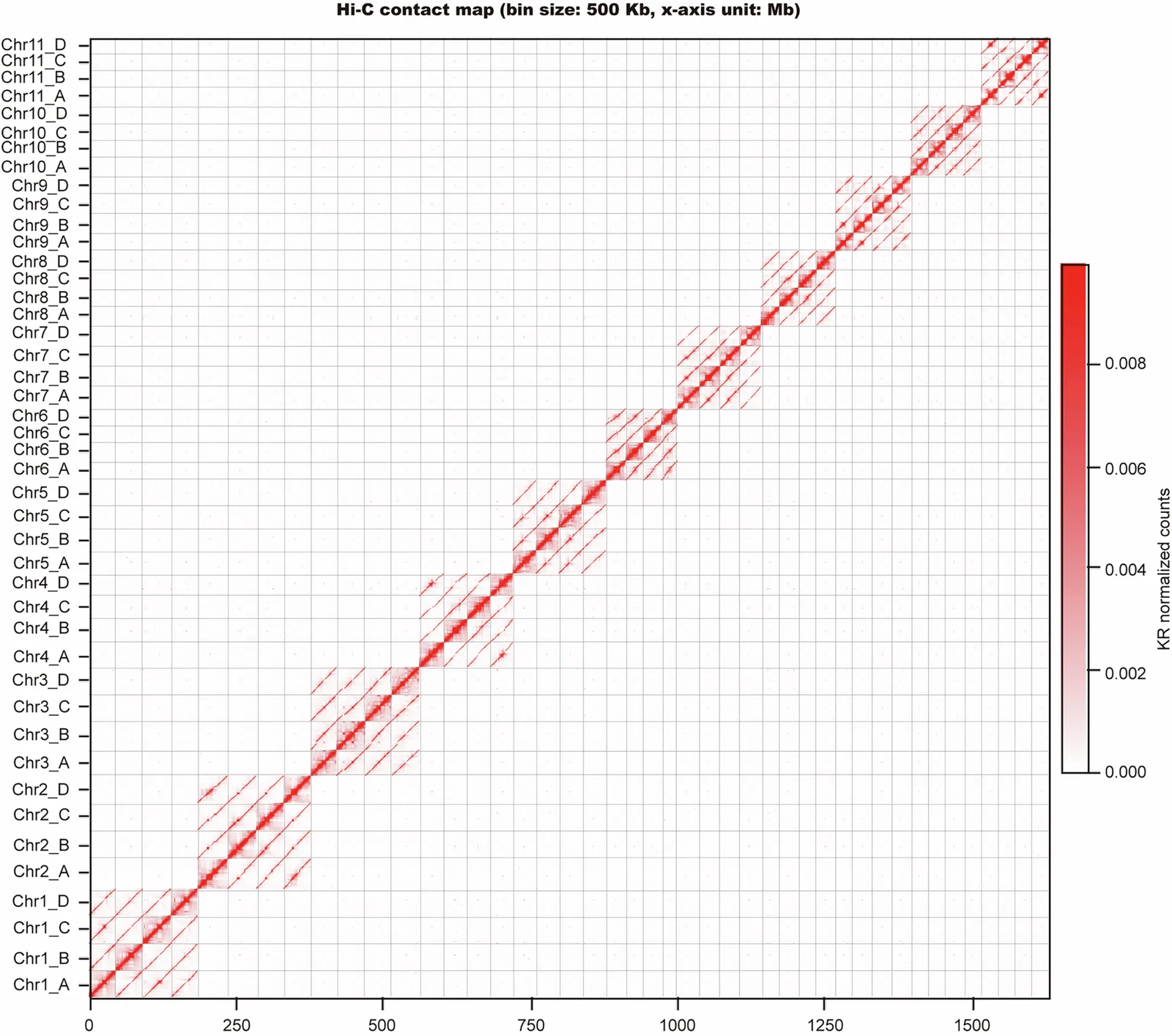
Hi-C contact maps of the final wax apple (*S. samarangense* ‘Tub’) genome assembly obtained by manual curation of the GPhase assembly. The prominent red blocks along the main diagonal represent high interaction frequencies within chromosomal bins, whereas off-diagonal signals suggest inter-chromosomal interactions.

There are two major differences between the new assembly and the previous version. One is a large inversion on chromosome 5, and the other involves a region originally assigned to chromosome 6 in the previous assembly, which is now placed on chromosome 11 in the new assembly. For the latter, synteny plots indicate that the phasing results of both HapHiC and ALLHiC are largely consistent with those of GPhase, although their assemblies in this region are less complete than those of GPhase (Fig. 5a). We performed further validation using IGV^46^ v.2.19.7 to determine which version is correct in these two cases (Extended Data Fig. 8a). We observed that, on chromosome 5, the boundaries of the inversion region in the new assembly exhibit uniform long-read coverage and are supported by numerous high-quality reads that span the breakpoint. In contrast, no such spanning reads are present at the corresponding region in the previous assembly, suggesting that the inversion is probably misrepresented in the previous version. Similarly, for the structural difference involving chromosomes 6 and 11, the boundary regions in the new assembly also show consistent and uniform read coverage, whereas the corresponding regions in the previous assembly exhibit abnormal read alignments and coverage patterns, further supporting the correctness of the new assembly. Moreover, Hi-C contact maps were used for further validation (Extended Data Fig. 8b). We observed that the inversion error on chromosome 5 in the previous assembly resulted in strong off-diagonal signals in the contact map, whereas no such signals were present in the corresponding region of the new assembly. Similarly, in the previous assembly, the misassignment of the genomic region from chromosome 11 to chromosome 6 led to abnormally strong inter-chromosomal Hi-C signals between this region and chromosome 11, which were even stronger than the intra-chromosomal signals between this region and the rest of chromosome 6. In contrast, the Hi-C map of the new assembly showed no such anomalies, supporting its structural accuracy (Extended Data Fig. 9).

To obtain the sequences of the rose genome, we generated HiFi reads and Hi-C reads as part of this study. The HiFi reads were assembled into unitigs using hifiasm. The collapse rate and heterozygosity rate of the assembly were 42.4% and 3.73%, respectively. Subsequently, haplotype-resolved scaffolding was performed on the unitigs using GPhase, HapHiC and ALLHiC, which utilized Hi-C data with or without the assistance of the assembly graph. The synteny plots show that the synteny between the GPhase assembly and the reference is more continuous than that of HapHiC and ALLHiC, indicating higher assembly accuracy (Supplementary Fig. 34). Moreover, consistent with observations from other datasets, the HapHiC and ALLHiC assemblies exhibit haplotype numbers inconsistent with the expected ploidy, such as chromosomes 2 through 7 in HapHiC and chromosomes 1 through 6 in ALLHiC, suggesting sequence loss and erroneous phasing. Similarly, BUSCO assessment shows that each haplotype set in the GPhase assembly exhibits high completeness, while the HapHiC and ALLHiC assemblies lack a substantial proportion of sequences (Fig. 5c). The HapHiC and ALLHiC assemblies also show higher rates of duplicated complete BUSCOs at the haplotype level than GPhase, indicating more frequent misassignment of sequences from multiple haplotypes into a single haplotype. We further performed manual curation based on the GPhase assembly, resulting in a final assembly of 1.59 Gb. The allele-specific Hi-C contact map, BUSCO assessment and synteny plots between haplotypes all indicate that this is a high-quality assembly of the rose genome (Fig. 5d,e and Supplementary Fig. 35).

**Extended Data Fig. 10:**
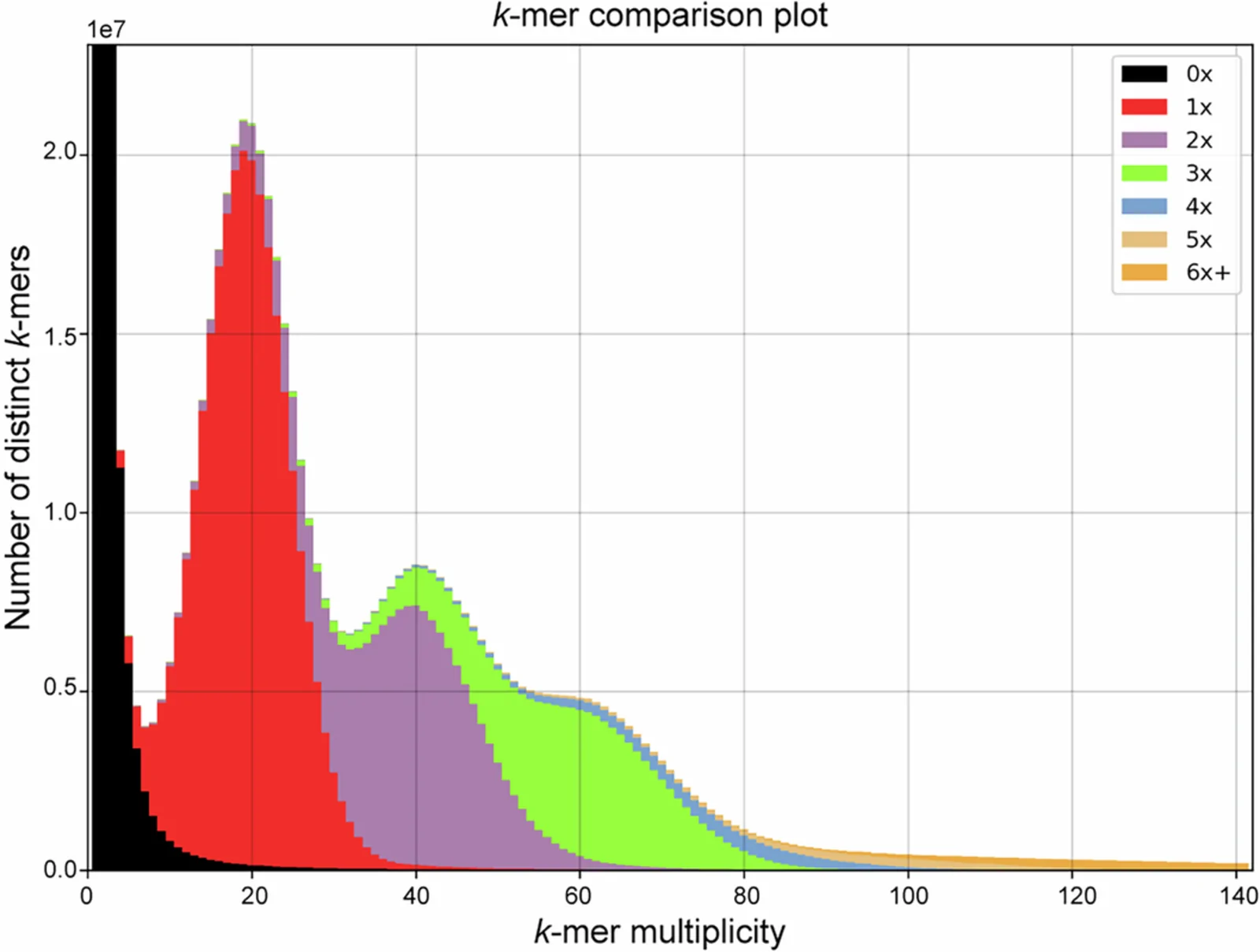
The KAT *k*-mer comparison plot for evaluating the completeness of final rose (*R. chinensis* ‘Jin Ou Fan Lv’) genome assembly. No black peak corresponding to missing *k*-mers was observed, suggesting a high completeness of the assemblies.

To further validate the quality of the newly assembled wax apple and rose genomes, we performed reference-free evaluations based on sequencing reads. First, both HiFi and short reads were aligned to the two assemblies, and assembly quality was assessed using CRAQ^47^ v.1.10 on the basis of the alignment results. The resulting quality scores were 93.535 for wax apple and 90.714 for rose. According to the CRAQ documentation, assemblies with quality scores above 90 are considered high quality. Second, assembly completeness was evaluated using KAT^48^ v.2.4.2. For both genomes, the results indicated that the assemblies captured the vast majority of *k*-mers present in the HiFi reads, with no black peaks corresponding to missing *k*-mers, suggesting high assembly completeness (Supplementary Fig. 36 and Extended Data Fig. 10). In addition, for the wax apple genome, we assessed switch errors by aligning ONT reads to the assembly, and the estimated switch-error rate was 0.37%.

## Discussion

Due to their high complexity and diversity, polyploid genome assembly has long been a major challenge in the field of genome assembly. To date, automated algorithms still struggle to produce high-quality assemblies for a substantial proportion of polyploid genomes, relative to those achieved for diploid genomes. Existing mainstream haplotype-resolved assembly methods for polyploid genomes mainly rely on Hi-C-based scaffolding tools such as ALLHiC and HapHiC, and these tools can also be applied to emerging 3C data types such as Pore-C and Omni-C. However, due to the inherent limitations of 3C data, these tools often fail to properly handle short and collapsed unitigs, resulting in sequence loss and phasing errors. In this study, we propose a new method that integrates assembly graph information to assist in the 3C-based phasing and assembly of polyploid genomes. On the basis of this approach, we developed a haplotype-resolved scaffolding algorithm named GPhase. Benchmarking on 15 complex polyploid genomes using different types of 3C data demonstrates that while state-of-the-art tools suffer from substantial phasing errors and sequence loss, GPhase consistently produces highly complete and accurate assemblies. GPhase demonstrates three major advantages over state-of-the-art tools. First, it generates haplotypes with well-balanced sizes, which is mainly attributed to two factors: the use of the assembly graph for the initial clustering of unitigs ensures accurate chromosome-level classification, providing a solid foundation for correct haplotype phasing; additionally, we designed effective algorithmic strategies to prevent collapsed unitigs from causing the misassignment of unitigs from different haplotypes into the same group. Second, GPhase produces more complete haplotype sequences. This is because the assembly graph is consistently leveraged throughout multiple steps of the pipeline: it helps preserve short unitigs during chromosome-level clustering; it is used again during the scaffolding stage to prevent the exclusion of short sequences; and it enables the identification of homologous regions, thereby ensuring that collapsed unitigs are correctly and fully assigned to their respective haplotypes, minimizing sequence loss. Third, the number of haplotypes produced by GPhase is highly consistent with the actual ploidy level of the genome. This advantage stems from a series of carefully designed adaptive tuning strategies applied during both chromosome-level unitig clustering and haplotype phasing, which ensure that the Louvain graph clustering algorithm can reliably generate the expected number of clusters, even under complex polyploid scenarios.

Although the current version of GPhase already fully leverages the information from the assembly graph, there is potential for further improvements to enhance the completeness and accuracy of the assembly. For instance, similar to chromosome-level unitig clustering, haplotype phasing could in theory benefit from an initial clustering step based on the assembly graph, which may improve the phasing accuracy of small unitigs. However, during the development of GPhase, we tested this approach and found that it did not lead to satisfactory results, and thus it was not integrated into the final algorithm. The main reason is that, unlike chromosome-level clustering—where unitigs from different chromosomes are usually well separated—the structure of the graph relevant to haplotype phasing is more complex. Homologous regions from different haplotypes often exhibit strong coupling in the graph, making it difficult to delineate subgraphs that clearly belong to a single haplotype. Future work should therefore focus on a deeper investigation and analysis of the graph structure, with the goal of developing more effective graph partitioning algorithms. One potential approach is to remove certain edges and paths connecting homologous unitigs, which may help reduce inter-haplotype coupling and enable clearer separation of haplotype-specific subgraphs.

The assembly graph structure may also be useful for identifying collapsed unitigs and estimating their copy numbers. For example, a unitig with multiple predecessors and multiple successors is often indicative of a collapsed region, and the number of such connections may provide an estimate of its copy number. However, in practice, the situation is often more complex. Collapsed regions may not consist of a single unitig but instead form a path composed of multiple unitigs. Due to sequence similarity, such paths may also be entangled with other regions of the genome, resulting in complex graph connectivity. Although previous studies have demonstrated successful cases of using graph structure to identify collapsed unitigs in specific genomes^13^, implementing this strategy in a general-purpose polyploid assembly algorithm remains challenging and would require more sophisticated algorithmic designs.

A potentially more powerful solution is to integrate 3C signals and homology information directly into the assembly graph, forming a unified graph model that incorporates multiple layers of information. In this model, different types of connections—such as sequence adjacency, 3C interactions or homologous relationships—can be represented as distinct types of edges, while the strength of each signal can be encoded as edge weights. This unified framework would allow chromosome partitioning, haplotype phasing and scaffolding to be reformulated and jointly solved as a graph optimization problem. One major challenge of this approach lies in determining the appropriate weighting of different types of information in a unified optimization model. Since the reliability and relevance of each signal vary across different genomic regions and tasks, and their numerical scales are often incompatible, assigning equal weights would be suboptimal. Moreover, the relative importance of each signal may change depending on the specific subproblem (for example, phasing versus scaffolding), meaning that the weights should not remain fixed across all steps. These optimal parameters will need to be empirically estimated and iteratively refined through future large-scale analyses across diverse polyploid genomes.

As future work, GPhase could be extended to support a broader range of data types. Although it currently leverages Hi-C, Pore-C and Omni-C data, the diversity of 3C-based technologies is rapidly expanding, and emerging data types such as HiFi-C remain to be explored and evaluated. The ability to jointly use multiple complementary data sources is expected to further enhance the accuracy and robustness of polyploid genome assembly with GPhase.

To conclude, the GPhase algorithm substantially improves the assembly quality of complex polyploid genomes and represents a successful attempt to tackle the challenges of polyploid phasing and assembly by leveraging assembly graph information. We believe that this integrative approach—combining assembly graphs, 3C data and other auxiliary signals—represents a promising future direction for polyploid genome assembly. Such a framework has the potential to dissolve the traditional boundary between contig/unitig construction and scaffolding, merging them into a unified assembly process. With further algorithmic advancements, this strategy is expected to significantly enhance the accuracy and completeness of polyploid assemblies, paving the way for achieving T2T assemblies in complex polyploid genomes.

## Methods

### 3C data normalization and unification

GPhase implements a unified and standardized preprocessing pipeline for 3C data, including Hi-C, Omni-C and Pore-C, with the goal of accurately quantifying and correcting the interaction strengths between unitigs. For Hi-C and Omni-C data, GPhase directly accepts BAM or Pairs format files generated by commonly used aligners such as BWA^49^ or Chromap^50^. For Pore-C data, which are characterized by multi-way contacts, GPhase employs the previously published PPL-Toolbox^51^ v.0.1.1 to extract high-confidence pairwise interactions and converts them into the standard Pairs format.

To mitigate the effects of non-uniform restriction enzyme cutting site distribution across the genome and biases introduced by sequence length differences on the estimation of spatial interaction strength, GPhase provides three optional normalization modes to quantify the linkage strength between unitigs.

Mode I (default), restriction-enzyme-density-based normalization, corrects raw interaction counts by the abundance of restriction enzyme recognition sites per unit length. The linkage weight between unitigs *u* and *v* is defined as:

$${\mathrm{Links}}_{u,v}=\frac{{N}_{{\rm{reads}}}}{{R}_{u}/{L}_{u}\times {R}_{v}/{L}_{v}}$$

where Links_*u*,*v*_ denotes the linkage weight between unitigs *u* and *v*, *N*_reads_ is the number of valid interaction read pairs, *R*_*u*_ and *R*_*v*_ are the numbers of restriction enzyme recognition sites on the corresponding unitigs, and *L*_*u*_ and *L*_*v*_ represent the unitig lengths.

Mode II, length-based normalization, focuses on correcting the statistical bias arising from the higher physical collision probability of longer sequences. The linkage weight is defined as:

$${\mathrm{Links}}_{u,v}=\frac{{N}_{\mathrm{reads}}}{{L}_{u}\times {L}_{v}}$$

In Mode III, no normalization, the number of valid interaction reads is directly used as the linkage weight between unitigs.

Through this preprocessing and normalization framework, GPhase converts heterogeneous 3C sequencing data into a high-quality unitig interaction matrix, providing robust and reliable topological constraints for subsequent haplotype clustering and chromosome-level anchoring.

### Chromosome-level graph partitioning

In Step 1, GPhase partitions the unitig-level assembly graph into subgraphs, each representing a chromosome. First, the unitig graph is divided into small subgraphs with simple branching structures that are confidently assigned to a single chromosome on the basis of the graph topology. To achieve this, branching nodes with multiple successors or predecessors are iteratively examined. For a node *v* with multiple successors, the *N*-order neighbourhoods of each successor *u*_*i*_ are identified using breadth-first search. If any pair of these neighbourhoods are disjoint, all outgoing edges from *v* are removed. This process is repeated for a predefined number of iterations and applied in the same way to nodes with multiple predecessors after reversing edge directions.

Second, GPhase filters out small subgraphs. For each subgraph, the total length of all constituent unitigs is calculated as its size, and the N90 value is determined across all subgraph sizes. Subgraphs with sizes below the N90 threshold are discarded.

Third, GPhase clusters subgraphs according to sequence homology. Unitig–unitig *k*-mer similarity is first computed using Partig, and homologous unitigs are identified. Two unitigs, *C*_*i*_ and *C*_*j*_, are considered homologous if their lengths exceed 50 kb and their *k*-mer similarity *S*_*ij*_ surpasses a predefined threshold *T*. Homologous unitigs are also detected on the basis of graph topology by identifying unitig pairs that share identical predecessor and successor nodes, with *S*_*ij*_ set to 1 in such cases. A similarity network of subgraphs is then constructed, in which each node represents a subgraph and the weight of an edge connecting subgraphs *G*_*i*_ and *G*_*j*_, containing unitig sets *C*_*i*_ and *C*_*j*_, respectively, equals the sum of homology signals for all homologous unitig pairs between *C*_*i*_ and *C*_*j*_: *W*(*G*_*i*_, *G*_*j*_) = Σ*S*_*ij*_, where the summation is over all *c*_*i*_ ∈ *C*_*i*_ and *c*_*j*_ ∈ *C*_*j*_ identified as homologous. The Louvain community detection algorithm is subsequently applied to this network to generate an initial clustering, merging subgraphs that belong to the same cluster.

Fourth, since the number of clusters at this stage still exceeds the actual chromosome count, GPhase further merges subgraphs using 3C contact signals. For each subgraph ${G}_{i}^{\prime}$ obtained from the previous step, its 3C contact strength with another subgraph ${G}_{j}^{{\prime} }$ is defined as ${W}_{{ij}}^{3{\mathrm{C}}}={\sum }_{u\in {G}_{i}^{{\prime} },v\in {G}_{j}^{{\prime} }}{H}_{{uv}}$, where *H*_*uv*_ denotes the 3C linkage intensity between unitigs *u* and *v*. A new network is then constructed with subgraphs as nodes and 3C contact strengths as edge weights. Louvain community detection is applied to this network to merge subgraphs within the same cluster, yielding chromosome-scale clusters. To obtain the expected number of clusters, a binary search is used to adjust the Louvain resolution parameter *γ*, iteratively minimizing the difference between the current number of clusters and the expected chromosome count until convergence.

Fifth, GPhase retrieves the previously filtered small subgraphs and assigns them to chromosomes. For each subgraph *G*_*r*_ to be rescued, the total 3C contact strength with each chromosome cluster *C*_*k*_ is calculated as ${S}_{{rk}}^{3{\mathrm{C}}}={\sum }_{u\in V\left({G}_{r}\right),v\in V\left({C}_{k}\right)}{H}_{{uv}}$, where *V*(*G*_*r*_) and *V*(*C*_*k*_) denote the sets of unitigs in *G*_*r*_ and *C*_*k*_, respectively, and *H*_*uv*_ represents the 3C linkage intensity between unitigs *u* and *v*. Each subgraph *G*_*r*_ is then assigned to the cluster *C*_*k*_ with the highest total 3C contact strength.

### Haplotype phasing

In Step 2, GPhase performs haplotype phasing for each chromosome. First, GPhase identifies collapsed unitigs. It supports user-provided unitig copy numbers or calculates them using popCNV. Unitigs with copy numbers of 0 or 1 are regarded as non-collapsed unitigs, denoted as *C*_non_, while those with copy numbers greater than 1 are considered collapsed unitigs, denoted as *C*_col_.

Second, GPhase applies a local clustering approach to group the non-collapsed unitigs. We constructed a global 3C interaction network in which nodes represent unitigs and edge weights correspond to 3C contact strengths. From this, we built an undirected graph *G* = (*V*, *E*, *W*), where *V* = *C*_non_ comprises all non-collapsed unitigs, *E* represents 3C links between unitigs and initial edge weights were set to *W*_*ij*_ = 0. For each collapsed unitig *c* ∈ *C*_col_, we extracted its first-order neighbours within *C*_non_ (*V*_1−nei_) to construct a local 3C network *N*_*c*_ = (*V*_*c*_, *E*_*c*_). Here, *V*_*c*_ = {*i* ∈ *V*_1−nei_|*H*_*ic*_ > 0} denotes non-collapsed unitigs connected to *c*, and *E*_*c*_ = {(*i*, *j*)|*i*, *j* ∈ *V*_*c*_, *H*_*ij*_ > 0} denotes 3C links among these neighbours. The Louvain community detection algorithm (with the default resolution parameter) was applied to each local 3C network to cluster the non-collapsed unitigs.

The resulting clusters were then refined using a homology-based correction strategy: if a pair of homologous unitigs *i* and *j* were assigned to the same cluster *C*_*k*_, the cluster was considered conflicted. In such cases, GPhase calculated the total 3C contact strength of *i* and *j* with the other unitigs in *C*_*k*_, denoted as ${H}_{i}={\sum }_{v\in {C}_{k},v\ne i,\,j}{H}_{{iv}}$ and ${H}_{j}={\sum }_{v\in {C}_{k},v\ne i,j}{H}_{{jv}}$, respectively. If *H*_*i*_ < *H*_*j*_, *j* was retained in *C*_*k*_, while *i* was removed and reassigned to the cluster C* with which it had the strongest 3C connection. After correction, the edge weight between any two nodes *i*, *j* ∈ *N*_*c*_ within the same cluster was updated as *W*_*ij*_ = *W*_*ij*_ + 1. This procedure was repeated for all collapsed unitigs, resulting in a final clustering network *G* = (*V*, *E*, *W*) that integrates multiple layers of local 3C information. The Louvain community detection algorithm was applied to network *G*, and the resolution parameter *γ* was optimized by binary search to yield a clustering *P* with the expected number of haplotypes *m*. The clustering was then evaluated for correctness using homology information. For each cluster *P*_*k*_(*k* = 1, 2, …, *m*), if any unitig pair (*u*, *v*) ∈ *P*_*k*_ × *P*_*k*_ corresponded to a homologous unitig pair, the shorter of *u* and *v* was considered misclustered. The lengths of all such misclustered unitigs were summed, and if the total exceeded 1 Mb, the clustering result was deemed unreliable.

Third, if the local clustering approach produced unreliable results, GPhase clustered the non-collapsed unitigs directly on the basis of 3C contacts. All non-collapsed unitigs were included in the node set *V* = *C*_non_, and an undirected graph *G* = (*V*, *E*, *W*) was constructed using 3C contact strengths *H*_*ij*_ as edge weights (*W*_*ij*_ = *H*_*ij*_) for each pair of unitigs *i*, *j* ∈ *V*. The Louvain community detection algorithm was then applied, with the resolution parameter *γ* optimized by binary search to match the expected number of haplotypes. As before, clustering correctness was evaluated using homology information, and results with a total misclustered length exceeding 1 Mb were deemed unreliable.

Fourth, when neither the local nor direct clustering approaches yielded satisfactory results, GPhase further optimized clustering by filtering 3C contacts. All edges in the global 3C interaction network were first ranked by contact strength, and the kneed^52^ algorithm was used to identify the inflection point in the strength distribution as the maximum filtering threshold *θ*_max_. Initially, no filtering was applied. If both clustering approaches failed to produce the expected number of haplotype clusters, the threshold was incrementally increased by steps of 1, and the clustering procedures were repeated with filtered 3C contacts. If no satisfactory result was obtained after reaching *θ*_max_, the clustering with the smallest total misclustered length among all historical results was selected as the final output.

Fifth, after clustering the non-collapsed unitigs, GPhase assigns the collapsed unitigs using a joint strategy that integrates 3C contact signals and homology information to improve assignment accuracy. For a collapsed unitig *u*, let the set of non-collapsed unitig clusters be *C* = *C*_1_, *C*_2_, …, *C*_*K*_. For each cluster *C*_*k*_ ∈ *C*, the total 3C contact strength between *u* and *C*_*k*_ is computed as $${H}_{k}\left(u\right)={\sum }_{v\in {C}_{k}}{H}_{{uv}}$$, and the total length of unitigs in *C*_*k*_ homologous to *u* is calculated as $${L}_{k}\left(u\right)={\sum }_{v\in {C}_{k},{S}_{{uv}} > \theta }{\mathrm{len}}\left(v\right)$$, where *S*_*uv*_ denotes the homology similarity between *u* and *v*, *θ* is the similarity threshold and len(*v*) is the sequence length of *v*. The 3C contacts are first examined for significant enrichment: $${H}_{\max }\left(u\right)$$$$=\max \left({H}_{k}\left(u\right)\right)$$ and $${H}_{{\mathrm{avg}}}\left(u\right)$$$$=\frac{1}{K-1}{\sum }_{k\ne {k}^{* }}{H}_{k}\left(u\right)$$, where $${k}^{* }={\mathrm{argmax}}\left({H}_{k}\left(u\right)\right)$$. If *H*_max_(*u*) > *N* × *H*_avg_(*u*), with *N* being a significance multiplier, *u* is assigned to cluster $${C}_{{k}^{* }}$$. Otherwise, *u* is assigned to the cluster with the smallest total homologous length *L*_*k*_(*u*).

### Contig assembly and scaffolding

In Step 3, GPhase assembles unitigs into contigs and performs chromosome-level scaffolding for each haplotype. First, it merges the haplotype-specific subgraphs generated during the assembly graph partitioning in Step 1 into larger and more reliable subgraphs by connecting them through trustworthy edges and paths. From the assembly graph, GPhase extracts the connectivity relationships among the subgraphs containing the target unitigs (that is, unitigs belonging to the haplotype of interest) and constructs a genome-wide connectivity network *G* = (*V*, *E*), where *V* denotes the set of nodes (each representing a subgraph) and *E* represents the set of edges, with an edge *e*_*ij*_ ∈ *E* added if a connection exists between two subgraphs in assembly graph. Let *S* ⊆ *V* be the subset of nodes whose corresponding subgraphs in the assembly graph contain at least one target unitig. A connectivity network of the target subgraphs, *G*′ = (*S*, *E*′), is then constructed. For each pair (*s*_*i*_, *s*_*j*_) ∈ *S* × *S*, if a unique path exists between *s*_*i*_ and *s*_*j*_, Dijkstra’s algorithm is used to compute the path *P*(*s*_*i*_, *s*_*j*_). The distance *d*(*s*_*k*_, *s*_*j*_) between the penultimate target subgraph *s*_*k*_ on *P*(*s*_*i*_, *s*_*j*_) and *s*_*j*_ is then evaluated; if *d*(*s*_*k*_, *s*_*j*_) < *T*, where *T* is a predefined threshold, an edge ${e}_{{s}_{k},{s}_{j}}^{{\prime} }\in {E}^{{\prime} }$ is added, yielding the connectivity network *G*′ of the target subgraphs.

For each connected component of *G*′, a topological sort is performed to obtain an ordered sequence ${\rm{\sigma }}=\{{v}_{1}^{{\prime} },{v}_{2}^{{\prime} },\ldots ,{v}_{n}^{{\prime} }\}$. The sequence is traversed, and if two adjacent nodes $\left({v}_{i}^{{\prime} },{v}_{i+1}^{{\prime} }\right)$ are not connected, or if ${v}_{i}^{{\prime} }$ has more than one successor or ${v}_{i+1}^{{\prime} }$ has more than one predecessor, the sequence is split into two subsequences $\{{v}_{1}^{{\prime} },{v}_{2}^{{\prime} },\ldots ,{v}_{i}^{{\prime} }\}$ and $\{{v}_{i+1}^{{\prime} },{\ldots ,v}_{n}^{{\prime} }\}$. This process results in a set of subsequences {*σ*_1_, *σ*_2_, …, *σ*_*m*_}, where the subgraphs within each subsequence *σ*_*l*_ are merged into a larger connected subgraph.

Second, scaffolds are generated for each merged connected subgraph. For each connected subgraph, the set of target unitigs *U*_*l*_ is retrieved, and a unitig connectivity network *G*_*u*_ = (*V*_*u*_, *E*_*u*_) is constructed, where *V*_*u*_ is the set of nodes, each representing a target unitig. For every pair of target unitigs (*u*_*i*_, *u*_*j*_) ∈ *U*_*l*_ × *U*_*l*_, the shortest path *P*(*u*_*i*_, *u*_*j*_) is computed. If the distance *d*(*u*, *u*_*j*_) between the penultimate target unitig *u* on *P*(*u*_*i*_, *u*_*j*_) and *u*_*j*_ is less than the threshold *T*, an edge ${e}_{u,{u}_{j}}\in {E}_{u}$ is added, resulting in the unitig connectivity network *G*_*u*_. Cycles in *G*_*u*_ are removed by deleting the edge with the lowest 3C signal strength, thereby converting the graph into a directed acyclic graph. A topological sort is then performed on the directed acyclic graph to obtain an ordered sequence *τ* = {*u*_1_, *u*_2_, …, *u*_*m*_}. The sequence is traversed, and if two adjacent unitigs (*u*_*i*_, *u*_*i*+1_) are not connected, it is split into subsequences {*u*_1_, …, *u*_*i*_} and {*u*_*i*+1_, …, *u*_*m*_}, ultimately yielding a series of subsequences {*τ*_1_, *τ*_2_, …, *τ*_*p*_}. Finally, for each subsequence *τ*_*q*_ = {*u*_1_, *u*_2_, …, *u*_*t*_}, Dijkstra’s algorithm is applied to determine the shortest path *P*(*u*_*i*_, *u*_*i*+1_) between each pair of adjacent unitigs (*u*_*i*_, *u*_*i*+1_), and the relative orientation of unitigs is inferred on the basis of the directions of the edges along the path. Each subsequence ultimately forms a scaffold. In this process, non-target unitigs located on the shortest path are rescued back into the haplotype, and consecutive unitigs within each scaffold that are directly connected by edges in the assembly graph are merged into contigs.

Third, GPhase leverages 3C signals to further perform chromosome-level scaffolding of the assembled scaffolds. Initially, the YaHS algorithm is applied with the *a* parameter for scaffolding, followed by the sort module of HapHiC to finalize the placement of scaffolds onto chromosome-level sequences.

### Comparison and evaluation

All details of the comparison and evaluation of GPhase, including the construction of real and simulated datasets, genome assembly procedures, validation methods and evaluation metrics, are provided in Supplementary Notes 2–4 and Supplementary Tables 2–6.

### Reporting summary

Further information on research design is available in the Nature Portfolio Reporting Summary linked to this article.

## Supplementary information

Supplementary Information Supplementary Notes 1–4 and Figs. 1–36.

Reporting Summary

Peer Review File

Supplementary Table 1 Sequence-alignment-based assessment scores of the GPhase phasing results in the stepwise performance evaluation.

Supplementary Table 2 Data sources of the reference genomes and ground-truth genomes.

Supplementary Table 3 Overview of the raw sequencing data.

Supplementary Table 4 Unitig-level assembly metrics across the 15 datasets.

Supplementary Table 5 Metrics for haplotype-resolved contig-level assembly of *Rosa hybrida* ‘Samantha’ generated by the hifiasm polyploid module.

Supplementary Table 6 Metrics for haplotype-resolved contig-level assembly of *Syzygium samarangense* ‘Tub’ generated by the hifiasm polyploid module.

Supplementary Table 7 Scaffold-level assembly metrics across the 15 datasets.

## Data Availability

All newly generated sequencing data used in this study, together with the final chromosome-level assemblies of *R. chinensis* ‘Jin Ou Fan Lv’ and *S. samarangense* ‘Tub’, have been deposited in the National Genomics Data Center (NGDC) (https://ngdc.cncb.ac.cn) under BioProject ID PRJCA045971. For *R. chinensis* ‘Jin Ou Fan Lv’, both PacBio HiFi reads (CRR2125246) and Hi-C reads (CRR2125247) were generated in this study. Manual curation yielded the final chromosome-level assembly (GWHGRYV00000000), and the reference sequence is available at https://iris.angers.inra.fr/obh (ref. ^53^). For *S. samarangense* ‘Tub’, ONT ultra-long reads (CRR2125340) were generated in this study, whereas the PacBio HiFi reads (SRR23279229 and SRR23279230) and Hi-C reads (CRR568487) together with Illumina reads (CRR568488, CRR568489 and CRR568490) were obtained from published datasets. Manual curation yielded the final chromosome-level assembly, version 2 (GWHGQMQ00000000); the previously published reference genome, version 1, is recorded in the NGDC Genome Warehouse under accession GWHDVFZ00000000 (https://ngdc.cncb.ac.cn/gwh). The newly generated PacBio HiFi, ONT ultra-long and Hi-C data for the haploid rice benchmark datasets based on *O. sativa* YF47 (CRR2127807, CRR2127808 and CRR2127809) and FL478 (CRR2127804, CRR2127805 and CRR2127806) were used to construct simulated benchmark datasets. The corresponding ground-truth assemblies are available at https://huggingface.co/datasets/YANchunX/Assemblies_generated_for_the_GPhase_benchmark_datasets/blob/main/rice_ground_truth/YF47.ref.fasta.gz and https://huggingface.co/datasets/YANchunX/Assemblies_generated_for_the_GPhase_benchmark_datasets/blob/main/rice_ground_truth/FL478.ref.fasta.gz, respectively. The remaining benchmark datasets were based on published sequencing data. These include the polyploid potato datasets *S. tuberosum* ‘Otava’ and ‘Katahdin’, the polyploid persimmon dataset *D. kaki* Thunb. ‘Xiaoguotianshi’, the polyploid alfalfa datasets *M. sativa* ‘Xinjiangdaye’ and ‘Zhongmu-4’, and the additional published datasets *R. hybrida* ‘Samantha’ and *Z. mauritiana* ‘Xishuangyesheng’. For *R. hybrida* ‘Samantha’, PacBio HiFi reads (SRR28905077), ONT ultra-long reads (SRR28905048), Pore-C reads (SRR28905076), Hi-C reads (SRR28905065) and MGI paired-end reads (SRR28905054) were obtained from NCBI; the reference assembly and annotation are available at https://iris.angers.inra.fr/obh, and the ground-truth assembly is available via Figshare at https://doi.org/10.6084/m9.figshare.22774097 (ref. ^54^). For *S. tuberosum* ‘Otava’, PacBio HiFi reads (SRR15206231) and Hi-C reads (SRR15198317, SRR15248701 and SRR15248702) were obtained from NCBI; the reference sequence is available at http://spuddb.uga.edu/c88_potato_download.shtml, and the ground-truth assembly is represented by JAIVGA000000000, JAIVGB000000000, JAIVGC000000000 and JAIVGD000000000, which can be retrieved from NCBI. For *S. tuberosum* ‘Katahdin’, PacBio HiFi reads (SRR32121363) and Omni-C reads (SRR32121374) were obtained from NCBI; the reference sequence is available at http://spuddb.uga.edu/c88_potato_download.shtml, and the ground-truth assembly is available via Zenodo at https://doi.org/10.5281/zenodo.14053896 (ref. ^55^). The three additional rice genomes used to construct simulated datasets—*O. sativa* BD8, Nipponbare and 9311—were also based on published data. For *O. sativa* BD8, PacBio HiFi reads (CRR1720551), ONT reads (CRR1720552) and Hi-C reads (CRR1720553) were obtained from NGDC, and the ground-truth genome is GWHFWBB00000000. For *O. sativa* Nipponbare, PacBio HiFi reads (SRR25241090), ONT ultra-long reads (SRR25241091) and Hi-C reads (SRR25241089) were obtained from NCBI, and the ground-truth/reference genome is available at http://www.ricesuperpir.com/web/nip (ref. ^33^). For *O. sativa* 9311, PacBio HiFi reads (CRR1644118), ONT ultra-long reads (CRR1644119) and Hi-C reads (CRR1644120) were obtained from NGDC, and the ground-truth genome is GWHFLAP00000000. For *D. kaki* Thunb. ‘Xiaoguotianshi’, PacBio HiFi reads (SRR18500470, SRR18500471, SRR18500472, SRR18500473, SRR18500474, SRR18500475, SRR18500476 and SRR18500477) and Hi-C reads (SRR18500481, SRR18500482, SRR18500483, SRR18500484, SRR18500485, SRR18500486, SRR18500487 and SRR18500488) were obtained from NCBI, and the ground-truth assembly is available via Figshare at https://doi.org/10.6084/m9.figshare.19447256 (ref. ^56^). For *Z. mauritiana* ‘Xishuangyesheng’, PacBio HiFi reads (SRR25180207 and SRR24951734) and Hi-C reads (SRR24951729) were obtained from NCBI, and the ground-truth assembly is available via Figshare at https://doi.org/10.6084/m9.figshare.23530068 (ref. ^57^). For *M. sativa* ‘Xinjiangdaye’, PacBio HiFi reads (SRR11285798) and Hi-C reads (SRR9026577) were obtained from NCBI, and the ground-truth assembly source is available via Figshare at https://doi.org/10.6084/m9.figshare.12327602 (ref. ^58^). For *M. sativa* ‘Zhongmu-4’, PacBio HiFi reads (CRR2374621), ONT ultra-long reads (CRR2374622) and Pore-C reads (CRR2374624) were obtained from NGDC; the reference assembly source is available via Figshare at https://doi.org/10.6084/m9.figshare.12327602, and the ground-truth assembly and annotation are available via Figshare at https://doi.org/10.6084/m9.figshare.30768716 (ref. ^59^). SRR accessions can be searched in NCBI (https://www.ncbi.nlm.nih.gov/), whereas CRR accessions can be searched in NGDC (https://ngdc.cncb.ac.cn). Comparative assemblies generated by the evaluated tools are archived at https://huggingface.co/datasets/YANchunX/Assemblies_generated_for_the_GPhase_benchmark_datasets/tree/main (ref. ^60^), and their corresponding assembly metrics and direct download links are provided in Supplementary Table 7. These comparative assemblies were generated exclusively to evaluate automated scaffolding results and were not manually corrected or curated; therefore, they are not intended for direct use as reference genomes.

## Code availability

GPhase and the associated example datasets are available via GitHub at https://github.com/panlab-bioinfo/GPhase. The source code is available via Zenodo at https://doi.org/10.5281/zenodo.21819422 (ref. ^61^).
