# Supplementary material for: Assembly-graph-assisted haplotype-resolved scaffolding of polyploid genomes
Source: Nat Plants. 2026 Oct 16;12(10):1960–75. doi: 10.1038/s41477-026-02422-5 (PMC13680272; doi:10.1038/s41477-026-02422-5)
Supplement: Supplementary file 1 [file 41477_2026_2422_MOESM1_ESM.pdf]

nature plants

Supplementary information

https://doi.org/10.1038/s41477-026-02422-5

Assembly-graph-assisted haplotype-resolved scaffolding of polyploid genomes

In the format provided by the authors and unedited

MANUSCRIPT TITLE

Assembly Graph-Assisted Haplotype-Resolved Scaffolding of Polyploid Genome

AUTHORS

Wenjie Du[1,3,†], Yanchun Li[1,2,†], Xiaofei Zeng[1,†], Jinbao Yang[1,†], Xingbin Wang[1], Xiaohui Zhang[1], Lianguang Shang[1], Xiaopeng Fu[4], Xiaoni Zhang[1], Xiu-qing Wei[5], Tingcai Cheng[3,*], Dongxi Li[2,*], Zhiqiang Wu[1,*], Weihua Pan[1,*]

AFFILIATIONS

[1] Shenzhen Branch, Guangdong Laboratory of Lingnan Modern Agriculture, Key Laboratory of Synthetic Biology, Ministry of Agriculture and Rural Affairs, Genome Analysis Laboratory of the Ministry of Agriculture and Rural Affairs, State Key Laboratory of Genome and Multi-omics Technologies, State Key Laboratory of Tropical Crop Breeding, Agricultural Genomics Institute at Shenzhen, Chinese Academy of Agricultural Sciences, Shenzhen 518124, China.

[2] College of Computer Science and Technology, Taiyuan University of Technology, Taiyuan 030024, China.

[3] State Key Laboratory of Resource Insects, Southwest University, Chongqing 400716, China.

[4] National Key Laboratory for Germplasm Innovation & Utilization of Horticultural Crops, College of Horticulture and Forestry Sciences, Huazhong Agricultural University, Wuhan 430070, China.

[5] Fruit Research Institute, Fujian Academy of Agricultural Sciences, Fuzhou, Fujian, 350002, China.

[†] These authors contributed equally.

*Corresponding author

Weihua Pan:

Email: panweihua@caas.cn

Zhiqiang Wu:

Email: wuzhiqiang@caas.cn

Dongxi Li:

Email: dxli0426@126.com

Tingcai Cheng:

Email: chengtc@swu.edu.cn

Table of Contents

Supplementary Note 1: Stepwise performance evaluation of GPhase 4
Results 4
Methods 5
Supplementary Note 2: Methods for Evaluating GPhase 6
Sequencing and dataset construction 6
Heterozygosity rate and collapse rate 8
Genome Assembly 8
Validation 9
Supplementary Note 3: Initial assembly and scaffolding details 14
Assembly of the two semi-simulated genomes 14
Assembly of the four fully simulated genomes 15
Assembly of the rose genome (*R. hybrida* 'Samantha') 15
Assembly of the potato genome (*S. tuberosum* 'Otava') 16
Assembly of the potato genome (*S. tuberosum* 'Katahdin') 17
Assembly of the wax apple genome (*S. samarangense* 'Tub') 17
Assembly of the rose genome (*R. chinensis* 'Jin Ou Fan Lv') 18
Assembly of the persimmon genome (*D. kaki Thunb* '*Xiaoguotianshi'*) 19
Assembly of the alfalfa genome (*M. sativa* 'X*injiangdaye'*) 19
Assembly of the jujube genome (*Z. mauritiana* '*Xishuangyesheng'*) 19
Assembly of the alfalfa genome (*M. sativa* 'Zhongmu-4') 19
Supplementary Note 4: Details of sequencing data simulation 21
References 22
Supplementary Figures 23

Supplementary Note 1: Stepwise performance evaluation of GPhase

Results

We validated the performance of each step of GPhase using the semi-simulated tetraploid genome. To this end, we constructed ground truth datasets corresponding to the intermediate outputs of the GPhase algorithm, such as chromosome-level partition of unitigs. Each of the three steps of GPhase was then executed and evaluated independently, using the original input of the GPhase algorithm and / or the corresponding ground-truth intermediate results as input. The first step takes the original set of unitigs, the unitig graph, and the Hi-C data as input, and produces a chromosome-level partition of the unitig graph. The ground-truth partition of unitigs was obtained by aligning them to the ground-truth chromosome sequences. Notably, the graph partitioning result produced by GPhase closely matches the ground truth, as observed through Bandage[1] v0.8.1 visualization (**Supplementary Fig. 10**), achieving sequence completeness ranging from 97.2% to 99.7% across all 12 chromosomes (**Supplementary Fig. 11**).

The second step takes the ground-truth chromosome-level partition of unitigs as input, performs haplotype phasing for each chromosome, and outputs a set of unitigs assigned to each haplotype. Due to the high sequence similarity between haplotypes, it is challenging to obtain an accurate haplotype-level ground truth for unitig phasing across all chromosomes by aligning unitigs to the ground-truth haplotype sequences. To evaluate the accuracy of GPhase's phasing results, RagTag[2] v2.1.0 was employed to scaffold the unitigs of each haplotype using the corresponding ground-truth haplotype sequence as a reference. The resulting haplotype assemblies showed high concordance with the ground truth, demonstrating the high accuracy of GPhase's haplotype phasing (**Supplementary Fig. 12 and Supplementary Table 1**). While a few chromosomes exhibited minor sequence omissions or translocations, these discrepancies are more likely attributable to scaffolding errors introduced by RagTag than to misclassification by GPhase.

Despite the challenge of obtaining an accurate haplotype-level ground truth for unitig phasing across all chromosomes, this is feasible for certain chromosomes and haplotypes that exhibit relatively high sequence divergence from the others. Accordingly, for one haplotype each of chromosomes 1, 10, and 11, the third step takes the ground-truth haplotype-specific unitig sets as input and generates chromosome-level assemblies together with the corresponding contig sequences

through scaffolding and assembly. We observed that the chromosome sequences generated by GPhase were highly consistent with the ground truth, demonstrating the high performance of the scaffolding step (**Supplementary Fig. 13**). Moreover, the unitigs of these three haplotypes were fully assembled into contigs, thereby improving the contiguity (N50) of gap-free sequences from 1.67 Mb, 2.49 Mb, and 2.31Mb to 6.76 Mb, 16.91 Mb, and 25.15 Mb, respectively.

Methods

In the validation of the chromosome partition step (Step 1), the assembly graph together with all unitigs were provided as input to the corresponding GPhase module, which outputs the unitigs assigned to each chromosome. The ground-truth unitig set for each chromosome was constructed as follows: partig was run with the parameter "-m 0.5" to calculate the sequence similarity of each unitig against the ground-truth chromosome sequences, and each unitig was assigned to the chromosome with the highest similarity. To ensure the reliability of the ground truth, we filtered out unitigs that exhibited high similarity to two or more haplotype-resolved reference sequences, and therefore did not include them in the ground-truth set. The correctness of the GPhase chromosome partition step was then evaluated by comparing the unitig set assigned to each chromosome by GPhase with the corresponding ground-truth unitig set.

In the validation of the haplotype phasing step (Step 2), the ground-truth unitig sets for each chromosome (constructed as described above) were provided to GPhase for haplotype phasing, yielding 48 haplotype-specific unitig sets in total. Since ground-truth unitig sets for all haplotypes are not available, we validated the phasing accuracy by performing reference-based scaffolding of each haplotype's unitigs against the ground-truth chromosome sequences using RagTag v2.1.0 with default parameters. The resulting scaffolds were then aligned to the ground-truth chromosome sequences, and synteny plots were generated using SyRI v1.7.1 with parameters "-i 90 -l 100k" to assess the correctness of haplotype phasing. Sequence-alignment-based quantitative metrics, including mismatch rate, mismatches per kb, and missing rate, were calculated in the same manner as described in the Methods section of the main text.

In the validation of the scaffolding step (Step 3), we selected one haplotype each from chromosomes 1, 10, and 11, and provided their ground-truth unitig sets as input to GPhase for scaffolding. The resulting chromosome sequences were then compared with the corresponding

ground-truth chromosome sequences. Synteny plots were generated using SyRI v1.7.1 with default parameters to assess the correctness of scaffolding. To obtain the ground-truth unitig set for each of the three haplotypes, unitigs were first assigned to the most similar ground-truth haplotype sequence based on sequence similarity. The unitig sets thus obtained were further refined using assembly graph topology, ensuring more accurate ground-truth unitig sets for validation.

Supplementary Note 2: Methods for Evaluating GPhase

Sequencing and dataset construction

We evaluated the performance of GPhase on two semi-simulated genomes constructed from real rice genomes, four fully simulated genomes constructed from software, as well as on nine real genomes, including two rose genomes (*Rosa hybrida* 'Samantha' and *Rosa chinensis* 'Jin Ou Fan Lv'), two potato genomes (*Solanum tuberosum* 'Otava' and 'Katahdin'), one wax apple genome (*Syzygium samarangense* 'Tub'), one Indian jujube (*Ziziphus mauritiana* 'Xishuangyesheng') genome, one persimmon (*Diospyros kaki* 'Xiaoguotianshi') genome, and two alfalfa genomes (*Medicago sativa* 'Zhongmu-4' and 'Xinjiangdaye').

To construct the semi-simulated dataset, the raw sequencing data (HiFi, ONT UL and Hi-C) and T2T genome assemblies of rice cultivars 'Nipponbare', '9311', and 'BD8' were obtained from previous study, whereas those of 'YF47' and 'FL478' were newly generated in this study. For 'YF47' and 'FL478', young leaves were collected from rice seedlings grown in the experimental field of the Agricultural Genomics Institute at Shenzhen, Chinese Academy of Agricultural Sciences (Guangdong Province, China). Genomic DNA was extracted using the DNeasy Plant Mini Kit (Qiagen) following the manufacturer's protocol. For PacBio HiFi sequencing, two SMRT cells were run on the PacBio Sequel II platform, and circular consensus reads were generated using CCS (https://github.com/PacificBiosciences/ccs, version 6.4.0) with default parameters. For Oxford Nanopore sequencing, DNA libraries were sequenced on the GridION X5 or PromethION platforms. Hi-C sequencing libraries were generated and sequenced using the Illumina NovaSeq platform. To assemble the raw sequencing data of each individual rice genome into ground truth sequences, contig-level assemblies were generated using hifiasm v0.19.5-r592 with the parameters "--primary -l0", incorporating both HiFi and ONT reads. Hi-C reads were aligned to the contigs using the

Juicer[3] v1.5 pipeline, and scaffolding was performed using the 3D-DNA[4] v201008 pipeline with default settings. Chimeric scaffolds were subsequently manually curated based on Hi-C contact maps using Juicebox[5] v1.11.08.

To construct the four fully simulated datasets, the *Oryza sativa* 'Nipponbare' (NIP) T2T genome was used as the reference, and four levels of sequence divergence (3%, 1%, 0.5%, and 0.2%) were defined. For each divergence level, three haplotypes exhibiting the corresponding divergence from NIP were simulated using a HapHiC script (https://github.com/zengxiaofei/HapHiC/blob/main/simulation/sim_haplotypes.py) and then combined with the original NIP T2T sequence to generate a simulated autotetraploid rice genome. Based on these simulated genomes, HiFi sequencing reads were generated at 90× coverage using Badread[6] v0.4.1, and Hi-C data were simulated at 200× coverage using sim3C[7] v0.7.1. Detailed protocols describing the generation of the HiFi and Hi-C data are presented in **Supplementary Note 4**.

To construct the nine real genome datasets, we generated HiFi and Hi-C data for *Rosa chinensis* 'Jin Ou Fan Lv', and ONT ultra-long (UL) reads for *Syzygium samarangense* 'Tub' as part of this study. The ONT UL reads of *Syzygium samarangense* 'Tub' were generated using the same Nanopore sequencing protocol as applied to the rice samples described above. Young leaves were collected from an individual tree grown in the experimental field of the Fujian Academy of Agricultural Sciences (Fujian Province, China). HiFi and Hi-C data for *Rosa chinensis* 'Jin Ou Fan Lv' were generated using sequencing protocols similar to those used for the rice samples. Young leaves were collected from plants grown in the Flower Garden of Huazhong Agricultural University (Hubei Province, China). The remaining real datasets were obtained from public databases. Among the nine real genomes, *Rosa hybrida* 'Samantha' and the two potato genomes have high-quality ground-truth assemblies of the same genomes available for sequence-alignment-based evaluations. The remaining six genomes have only reference sequences from the same or closely related species, which were therefore used for gene-based syntenic analyses. The sources of all ground-truth and reference sequences are listed in **Supplementary Table 2**.

The volume, depth, and sources of all raw sequencing data used in this study are summarized in **Supplementary Table 3**. Detailed assembly metrics for unitig-level and haplotype-resolved contig-level results are provided in **Supplementary Tables 4–6**.

Heterozygosity rate and collapse rate

Heterozygosity estimation in simulated autotetraploid rice was performed using k-mer analysis. The abundance of k-mers within the HiFi reads was calculated using Jellyfish[8] 2.3.1 with the parameters "-C -m 21 -s 1600000000". GenomeScope[9] 2.0 v1.0.0 was then employed to estimate the heterozygosity rate based on the k-mer abundance distribution.

To calculate collapse rate, unitig graph files (*.p_utg.gfa) were extracted from hifiasm assembly results and converted to FASTA format (*.p_utg.gfa.fa). Then popCNV (https://github.com/sc-zhang/popCNV, GitHub commit 1286362) was used to estimate the copy number for each unitig. Finally, the collapse rate was calculated as:

$$\text{Collapse Rate} = \frac{\sum_{k=2}^{P}(L_k \times k)}{\sum_{k=1}^{P}(L_k \times k)}$$

where P is the ploidy level, and L_k is the total length of all unitigs with copy number k.

Genome Assembly

In the comparative experiments of GPhase, HapHiC, and ALLHiC, and C-Phasing a similar assembly procedure was applied to all tested genomes. Unitigs were generated using hifiasm v0.24.0-r703 with default parameters. For the two semi-simulated genomes, the rose (*Rosa hybrida* 'Samantha') genome, *Medicago sativa* 'Zhongmu-4' genome, and the wax apple (*Syzygium samarangense* 'Tub') genome, both HiFi and ONT UL reads were used for unitig assembly, whereas the other genomes were assembled using HiFi reads only. These scaffolding tools were then respectively used to assemble the unitigs into chromosome-level assemblies using varying types of 3C data. For the rose (*Rosa hybrida* 'Samantha') genome, Hi-C and Pore-C data were used separately for scaffolding, with each assembly relying on only one data type at a time. The *Medicago sativa* 'Zhongmu-4' genome was scaffolded exclusively using Pore-C data, while the *Solanum tuberosum* 'Katahdin' genome was scaffolded using Omni-C data only. All other genomes were scaffolded using Hi-C data. In addition, the combined hifiasm + YaHS workflow was also applied to assemble the rose (*Rosa hybrida* 'Samantha') and wax apple (*Syzygium samarangense* 'Tub') genomes using Hi-C data. For each genome, hifiasm was first used to generate haplotype-resolved contig sets by integrating HiFi, ONT ultra-long, and Hi-C data, after which YaHS v1.2.2 was

employed to scaffold each haplotype independently. Further details are provided in the **Supplementary Note 3**.

During the subsequent assembly of the wax apple genome, chimeric scaffolds were manually corrected based on Hi-C contact maps visualized with Juicebox. Contig-level assemblies generated by Flye v2.9.3-b1797, Peregrine[10] v1.58.0, Verkko v2.2.1, and Hifiasm (HiFi-only mode) were first utilized to fill the gaps. Then, TGS-GapCloser[11] v1.2.1 was applied to further close the remaining gaps and improve the assembly contiguity. Also, during the subsequent assembly of the rose genome, chimeric scaffolds were manually corrected based on Hi-C contact signals visualized in Juicebox.

Validation

In the comparison between GPhase and other tools, BUSCO v5.8.3 was used to evaluate the completeness of the assemblies. For the semi-simulated genome and the wax apple (*Syzygium samarangense* 'Tub') genome, the Embryophyta lineage dataset (*embryophyta_odb10*) was used, while for the other genomes, the Eudicots lineage dataset (*eudicots_odb10*) was applied. To enable haplotype-level evaluation, all chromosome-scale sequences in each assembly were assigned to their corresponding haplotypes. Specifically, we first used *partig* in combination with *fixchr* to determine the chromosome correspondence and orientation between each assembled haplotype and the ground-truth haplotypes. For each chromosome, this step established the best-matching ground-truth haplotype and corrected orientation when necessary. The clustering of haplotypes across different chromosomes (i.e., determining which chromosome-level haplotypes belong to the same global haplotype group, such as Hap1, Hap2, etc.) was defined according to the haplotype grouping of the ground truth. These ground-truth haplotype groupings were generated by the original study authors using various approaches, including parental information or predefined haplotype assignments.

Gene-based syntenic dot plots were generated using JCVI[12] v1.4.16 with default parameters. For the semi-simulated genomes, the previously constructed T2T genome of rice Nipponbare was used as the reference. For the two rose genomes, the reference was the downloaded *Rosa chinensis* 'Old Blush genome OBDH 1.0[13]'. The two *Medicago sativa* genomes used the genome of the 'Xinjiangdaye' variety as the reference. For the two potato genomes, alignment was performed against the downloaded *C88*[14] reference genome. For the remaining genomes, the ground truth

sequences assembled in the previous study were used as the reference. If the reference genome is haplotype-resolved, one of the haplotype sequences was selected as the reference.

Sequence-alignment-based syntenic dot plots were generated by aligning each assembly to the corresponding ground-truth genome using MUMmer[15] v4.0.1. As in the BUSCO-based evaluation, chromosome-scale sequences in each assembly were first assigned to their corresponding haplotypes using the same haplotype-matching procedure described above. Subsequently, for each haplotype, the MUMmer tool's nucmer was used to align the assembly sequence with the ground truth. High-confidence alignments were filtered using the delta-filter parameters "-i 90 -l 10000". Finally, mummerplot was employed to visualize the syntenic dot plots for further analysis.

Hi-C interaction maps were constructed through two complementary pipelines. For ALLHiC and HapHiC assemblies, BWA-MEM was used to align Hi-C reads to unitigs, and the resulting unfiltered alignments were subsequently processed with Juicer v1.2 to generate Hi-C contact maps using the parameter "-q 0". For GPhase assemblies, Chromap was used to align Hi-C reads to unitigs, applying the parameters "--remove-pcr-duplicates -q 0", followed by Juicer-based contact map generation. The construction of Omni-C contact maps follows the same method as Hi-C contact maps, with the only difference being the use of Omni-C data instead of Hi-C data. For the Pore-C dataset, both GPhase and HapHiC use the default PPL-Toolbox v0.1.1 to process Pore-C data and generate the pairs-format alignment files required for heatmap generation, while C-Phasing v0.2.5.r291 uses its own script to process the Pore-C data. The *k*-mer distribution plots were generated using the haplotype_kmers.py (https://github.com/zengxiaofei/HapHiC/blob/main/simulation/haplotype_kmers.py, GitHub commit 431b7b6) script provided by HapHiC to assign *k*-mers in each haplotype to genomic bins of length 500000. The resulting data were then visualized using a custom script.

Sequence-alignment-based metrics were calculated using MUMmer (nucmer) alignments between the assembly and the ground truth obtained from the Sequence-alignment-based syntenic dot plots. Using the delta-filter function, alignments with over 90% identity and block lengths exceeding 1 kb were retained to remove noise and random matches from the genome. Subsequently, SyRI was used to count and measure the lengths of matched regions, translocation errors, inversion errors, and duplications. Let the total block lengths of matched regions, translocation errors,

inversion errors, and duplications in the ground truth be represented as Truth_len_M, Truth_len_T, Truth_len_I, and Truth_len_D, respectively. Similarly, let the total block lengths and numbers of these errors in the assembly be represented as Asm_len_T, Asm_len_I, Asm_len_D, and Asm_num_T, Asm_num_I, Asm_num_D. The total lengths of the reference and query sequences were denoted as Total_Truth_len and Total_Asm_len, respectively. Based on these values, the Mismatch Rate, #Mismatches per kb, and Missing Rate were calculated as shown in the following formulas:

$$\text{\#Mismatches per kb} = \frac{\text{Asm\_num\_T} + \text{Asm\_num\_I} + \text{Asm\_num\_D}}{\text{Total\_Asm\_len}/1000}$$

$$\text{Mismatch Rate} = \frac{\text{Asm\_len\_T} + \text{Asm\_len\_I} + \text{Asm\_len\_D}}{\text{Total\_Asm\_len}}$$

$$\text{Missing Rate} = 1 - \frac{\text{Truth\_len\_M} + \text{Truth\_len\_T} + \text{Truth\_len\_I} + \text{Truth\_len\_D}}{\text{Total\_Truth\_len}}$$

In addition, three *k*-mer-based metrics were used to compare the accuracy of the three scaffolding tools, including Sequence Error Rate (SER), Sequence Missing Rate (SMR), and Inter-unitig Average Distance Difference (IUADD). The computational procedures for these metrics are defined as follows. SER quantifies the proportion of misclassified *k*-mers in the assembly and is defined as SER = 1 - *Precision*. The *Precision* is calculated as

$$Precision = \frac{\sum_{c=1}^{N_c} \max_{\omega \in \Omega} |K_{c,\omega}|}{\sum_{c=1}^{N_c} |K_c|}$$

where N_c is the number of chromosomes in the assembly, Ω is the set of ground truth chromosomes, K_c is the set of *k*-mers appearing in chromosome c of the assembly, $K_{c,\omega}$ is the set of *k*-mers appearing in both chromosome c of the assembly and chromosome ω of the ground truth. SMR measures the proportion of true *k*-mers in the ground truth that are lost in the assembly and is defined as SMR = 1 – *Sensitivity*. The *Sensitivity* is calculated as

$$Sensitivity = \frac{\sum_{\omega \in \Omega} max_{c=1}^{N_c} |K_{c,\omega}|}{\sum_{\omega \in \Omega} |K_\omega|}$$

where N_c is the number of chromosomes in the assembly, Ω is the set of ground truth chromosomes, K_ω is the set of *k*-mers appearing in chromosome ω of the ground truth, $K_{c,\omega}$ is the set of *k*-mers

appearing in both chromosome c of the assembly and chromosome ω of the ground truth. IUADD evaluates positional deviations of k-mers in the assembly relative to the ground truth genome. To compute this metric, for each chromosome $\omega \in \Omega$, we select M_ω pairs of inter-unitig k-mers—i.e., k-mer pairs that are located on different unitigs in the assembly and are present in both the assembly and the ground truth. For each pair (denoted as the i-th pair), we compute $\delta_i^{(asb)}$ representing the genomic distance between the two k-mers in the assembly, and $\delta_i^{(truth)}$ representing the genomic distance between the same two k-mers in the ground truth. Then, IUADD is calculated as

$$IUADD = \frac{1}{|\Omega|} \sum_{\omega \in \Omega} \left(\frac{1}{M_\omega} \sum_{i=1}^{M_\omega} |\delta_i^{(asb)} - \delta_i^{(truth)}| \right)$$

where Ω is the set of ground truth chromosomes, M_ω is the number of inter-unitig k-mer pairs selected for chromosome ω. This metric reflects the average discrepancy in spatial organization between the assembly and the ground truth at the inter-unitig level.

In the rose (*R. hybrida* 'Samantha') dataset, switch errors in haplotype-resolved genome assemblies produced by different tools were evaluated using PolySwitch, an ONT read–based assessment method (https://github.com/eden00Chen/polyswitch, GitHub commit 6cedfeb). PolySwitch was run with the parameter setting "-r 4000" to quantify haplotype switching events.

The final genome assembly of wax apple (*Syzygium samarangense* 'Tub'), after manu al curation, was comprehensively evaluated using multiple quality metrics and visualization approaches. The assembly quality was assessed by calculating the quality value (QV) wit h Merqury[16] v1.3, determining the LTR Assembly Index (LAI) using LTR_finder[17] v1.0.7 a nd LTR_retriever[18] v3.0.2, and generating Hi-C contact heatmaps using HapHiC_plot (https: //github.com/zengxiaofei/HapHiC/blob/main/scripts/HapHiC_plot.py, GitHub commit 0f05366) from the HapHiC visualization toolkit. Genome completeness was evaluated using BUSC O v5.8.3 with the Embryophyta lineage dataset (*embryophyta_odb10*). In addition, gene-bas ed syntenic dot plots were generated using JCVI[12] v1.4.16 with default parameters based o n the annotation files of the previously published wax apple genome. Furthermore, both H iFi and Illumina short reads were mapped to the final assembly and evaluated using CRA Q with default parameters to assess assembly correctness. Finally, HiFi reads were analyse

d with KAT under default settings to evaluate the completeness of the final assembly. Swi tch errors in the final assembly were also evaluated using the same ONT read-based strate gy as applied to the rose (*R. hybrida* ‘Samantha’) dataset.

The final genome assembly of *Rosa chinensis* ‘Jin Ou Fan Lv’, after manual curation, was evaluated using a combination of quality metrics and structural analyses. Assembly quality was visualized using Hi-C contact heatmaps generated by HapHiC_plot from the HapHiC visualization toolkit. Genome completeness was assessed using BUSCO v5.8.3 with the Eudicots lineage dataset (*eudicots_odb10*). Gene-based syntenic dot plots were generated using JCVI v1.4.16 with default parameters based on *Rosa* ‘Old Blush’ genome annotation files. In addition, synteny plots between the three haplotypes in the final assembly were drawn using SyRI[19] v1.7.1 with default settings. Both KAT-based completeness and CRAQ-based correctness assessments were performed using only HiFi reads as input. The experimental procedures and parameter settings followed those previously established for the final assembly of wax apple.

For validation of each assembly generated from the four fully simulated datasets, the sequence similarity between each unitig and the four corresponding simulated haplotypes was calculated to determine the haplotype assignment of each unitig. Unitigs shorter than 100 kb were filtered out to reduce interference from ambiguous matches among homologous sequences, and the remaining unitigs were assigned to the chromosome with the highest sequence similarity. We then quantified the number and length proportion of unitigs correctly clustered to their true chromosomes and haplotypes, and calculated Chromosome clustering accuracy (count), Chromosome clustering accuracy (length), Haplotype phasing accuracy (count), and Haplotype phasing accuracy (length). The number and total length of unitigs incorporated into the final assembly were used to compute the anchoring rate (count), and anchoring rate (length). The precise definitions of these metrics are provided in the legend of **Fig. 3**.

Supplementary Note 3: Initial assembly and scaffolding details

Here, we detail the assembly specifications for initial unitigs or haplotype-resolved contigs across the fifteen test datasets, alongside the specific scaffolding workflows and parameters implemented for each tool.

Assembly of the two semi-simulated genomes

The unitig-level assembly was generated using hifiasm v0.24.0-r703 with default parameters, integrating both HiFi and ONT reads.

GPhase scaffolding. Hi-C reads from the simulated datasets were aligned to unitigs using Chromap v0.2.7-r494 with the parameters --remove-pcr-duplicates -q 0 --SAM. Subsequently, the tetraploid semi-simulated dataset was processed through the GPhase pipeline using the parameters --rescue --reassign_number 1 --min_len 200 --cluster_q 1, while the triploid semi-simulated dataset was processed with the parameters --rescue --reassign_number 1 --min_len 200 --split_gfa_n 2.

HapHiC scaffolding. Hi-C reads were aligned to unitigs using Chromap with the parameter --remove-pcr-duplicates. For the tetraploid semi-simulated dataset, Hi-C reads were aligned to unitigs using Chromap with the parameter --remove-pcr-duplicates. Scaffolding was subsequently performed with HapHiC v1.0.6 using the parameters --remove_allelic_links 4 --max_inflation 5.0 --RE "GATC" --correct_nrounds 2 --gfa <species>.p_utg.gfa. For the triploid semi-simulated dataset, alignment was conducted with BWA-MEM v0.7.18-r1243-dirty using the parameter -5SP. The resulting BAM files were then filtered with HapHiC's filter_bam.pyscript. Finally, scaffolding was performed with HapHiC v1.0.6 using the parameters --remove_allelic_links 3 --max_inflation 5.0 --RE "GATC" --correct_nrounds 2.

ALLHiC scaffolding. For the tetraploid semi-simulated dataset and triploid semi-simulated dataset. The initial step involved constructing an allele table using GMAP[20] (2023-07-20) with the annotated Nipponbare genome as reference. Subsequent steps included: alignment with BWA-MEM v0.7.18-r1243-dirty using the parameter -5SP, BAM filtering with HapHiC’s filter_bam.py script (https://github.com/zengxiaofei/HapHiC/blob/main/utils/filter_bam.py), unitig partitioning with partition_gmap.py, and final chromosomal scaffolding with the ALLHiC v0.9.13 pipeline (https://github.com/tangerzhang/ALLHiC).

Assembly of the four fully simulated genomes

The unitig-level assembly was generated using hifiasm v0.24.0-r703 with default parameters, using HiFi reads.

GPhase scaffolding. Hi-C reads were aligned to unitigs using Chromap v0.2.7-r494 with the parameters --remove-pcr-duplicates -q 0 --SAM, and scaffolding was performed with the GPhase pipeline using the parameters --rescue --reassign_number 1 --min_len 200 --cluster_q 1.

HapHiC scaffolding.For the four fully simulated dataset, alignment was conducted with BWA-MEM v0.7.18-r1243-dirty using the parameters -5SP. The resulting BAM files were then filtered with HapHiC's filter_bam.py script. Finally, scaffolding was performed with HapHiC v1.0.6 using the parameters --remove_allelic_links 3 --max_inflation 5.0 --RE "GATC" --correct_nrounds 2.

ALLHiC scaffolding. For the four fully simulated dataset. The initial step involved co nstructing an allele table using GMAP (2023-07-20) with the annotated Nipponbare genom e as reference. Subsequent steps included: alignment with BWA-MEM v0.7.18-r1243-dirty using the parameter -5SP, BAM filtering with HapHiC's filter_bam.py script (https://github. com/zengxiaofei/HapHiC/blob/main/utils/filter_bam.py), unitig partitioning with partition_gma p.py, and final chromosomal scaffolding with the ALLHiC v0.9.13 pipeline (https://github.c om/tangerzhang/ALLHiC).

Assembly of the rose genome (*R. hybrida* 'Samantha')

Two types of initial assemblies were generated using hifiasm v0.24.0-r703. First, a unitig-level assembly was produced using default parameters, integrating both HiFi and ONT reads. Second, haplotype-resolved contigs were generated using the parameter "--n-hap 4", integrating HiFi, ONT, and Hi-C reads.

Hi-C scaffolding was performed on both the unitig-level assembly and the haplotype-resolved contigs.

Unitig-Based Scaffolding: GPhase, HapHiC, and ALLHiC scaffolding pipelines were applied to the unitig-level assembly. For GPhase scaffolding, Hi-C reads were aligned to unitigs using Chromap v0.2.7-r494 with the parameter --remove-pcr-duplicates, and scaffolding was performed through the GPhase pipeline with the parameters --rescue --reassign_number 1. For HapHiC scaffolding, Hi-C reads aligned to unitigs with Chromap (--remove-pcr-duplicates) were scaffolded

using HapHiC v1.0.6 with the parameters --remove_allelic_links 4 --max_inflation 3.0 --RE "GATC" --correct_nrounds 2 --gfa <species>.p_utg.gfa. For ALLHiC scaffolding, the initial step involved constructing an allele table using GMAP (2023-07-20) with the annotated OBDH genome as reference. Subsequent steps included alignment with BWA-MEM v0.7.18-r1243-dirty using the parameter -5SP, BAM filtering with HapHiC's filter_bam.py script, partitioning UTGs with partition_gmap.py, and final chromosomal scaffolding with the ALLHiC v0.9.13 pipeline.

Haplotype-Resolved Contig Scaffolding: For the haplotype-resolved contigs, each haplotype was scaffolded independently. Hi-C reads were aligned to the contigs of each haplotype using BWA-MEM v0.7.18-r1243-dirty with the parameters -5SP. Scaffolding was then performed using YaHS with default parameters.

Pore-C scaffolding was exclusively performed on the unitig-level assembly.

GPhase Scaffolding. Pore-C data were aligned to unitigs and converted to pairs format using PPL-Toolbox with default parameters. Scaffolding was then performed using GPhase with the parameters --rescue --reassign_number 1 --min_len 200 --cluster_q 1.

HapHiC Scaffolding. Pore-C data processing using PPL-Toolbox was identical to the GPhase workflow. Scaffolding was then performed using HapHiC with the parameters --remove_allelic_links 4 --max_inflation 5.0 --RE "GATC" --correct_nrounds 2 --gfa <species>.p_utg.gfa.

C-Phasing Scaffolding. Pore-C data were aligned to unitigs using the built-in alignment function of C-Phasing, followed by scaffolding with default parameters.

Assembly of the potato genome (*S. tuberosum* 'Otava')

The unitig-level assembly was generated using hifiasm v0.24.0-r703 with default parameters, using HiFi reads.

GPhase scaffolding. Hi-C reads were aligned to unitigs using Chromap v0.2.7-r494 with the parameters --remove-pcr-duplicates -q 0 --SAM, and scaffolding was performed with the GPhase pipeline using the parameters --cluster_q 1 --scaffold_q 0 --rescue --min_len 200 --processes 32 --split_gfa_n 5 --chr_pm 0.95 --hap_pm 0.6.

HapHiC scaffolding. Hi-C reads aligned to unitigs with Chromap (--remove-pcr-duplicates) were scaffolded using the HapHiC v1.0.6 pipeline with the parameters --remove_allelic_links 4 --

max_inflation 3.0 --RE "GATC" --correct_nrounds 2 --gfa <species>.p_utg.gfa.

ALLHiC scaffolding. An allele table was first constructed using GMAP (2023-07-20) with the annotated C88 genome as reference. Subsequent steps included alignment with BWA-MEM v0.7.18-r1243-dirty using the parameter -5SP, BAM filtering with HapHiC's filter_bam.py script (https://github.com/zengxiaofei/HapHiC/blob/main/utils/filter_bam.py), unitig partitioning with partition_gmap.py, and final chromosomal scaffolding with the ALLHiC v0.9.13 pipeline (https://github.com/tangerzhang/ALLHiC).

Assembly of the potato genome (*S. tuberosum* 'Katahdin')

The unitig-level assembly was generated using hifiasm v0.24.0-r703 with default parameters, using HiFi reads.

GPhase scaffolding. The 3C data is normalized using three different options of the --nor_hic parameter in the GPhase pipeline: ratio, length, and no. Then, the data is assembled using the parameters --rescue --reassign_number 1 --min_len 200 --cluster_q 1 of GPhase.

ALLHiC scaffolding. An allele table was first constructed using GMAP (2023-07-20) with the annotated C88 genome as reference. Subsequent steps included alignment with BWA-MEM v0.7.18-r1243-dirty using the parameter -5SP, BAM filtering with HapHiC's filter_bam.py script (https://github.com/zengxiaofei/HapHiC/blob/main/utils/filter_bam.py), unitig partitioning with partition_gmap.py, and final chromosomal scaffolding with the ALLHiC v0.9.13 pipeline (https://github.com/tangerzhang/ALLHiC).

Assembly of the wax apple genome (*S. samarangense* 'Tub')

Two types of initial assemblies were generated using hifiasm v0.24.0-r703. First, a unitig-level assembly was produced using default parameters, integrating both HiFi and ONT reads. Second, the ploidy of the wax apple genome was assessed using Smudgeplot v0.5.3. Subsequently, haplotype-resolved contigs were generated with the parameter "--n-hap 4" by integrating HiFi, ONT, and HiC reads.

Unitig-Based Scaffolding: GPhase, HapHiC, and ALLHiC scaffolding pipelines were applied to the unitig-level assembly. For GPhase scaffolding, Hi-C reads were aligned to unitigs using Chromap v0.2.7-r494 with the parameter --remove-pcr-duplicates, and scaffolding was performed

with the GPhase pipeline using the parameters --rescue --reassign_number 1.For HapHiC scaffolding, Hi-C reads were first aligned with BWA-MEM v0.7.18-r1243-dirty using the parameters -5SP, followed by BAM filtering with HapHiC's filter_bam.py. Scaffolding was then performed with the HapHiC v1.0.6 pipeline using the parameters --remove_allelic_links 4 --max_inflation 3.0 --RE "GATC" --correct_nrounds 2 --gfa <species>.p_utg.gfa --reassign_nrounds 3 --min_group_len 1. For ALLHiC scaffolding, An allele table was first constructed using GMAP (2023-07-20) with the annotated *Syzygium grande* genome as reference. Hi-C reads were then aligned with BWA-MEM v0.7.18-r1243-dirty using the parameters -5SP, followed by BAM filtering with HapHiC's filter_bam.py. Unitigs were partitioned with partition_gmap.py and scaffolded into chromosomal groups using the ALLHiC v0.9.13 pipeline (https://github.com/tangerzhang/ALLHiC).

Haplotype-Resolved Contig Scaffolding: For the haplotype-resolved contigs, each haplotype was scaffolded independently. Hi-C reads were aligned to the contigs of each haplotype using BWA-MEM v0.7.18-r1243-dirty with the parameters -5SP. Scaffolding was then performed using YaHS with default parameters.

Assembly of the rose genome (*R. chinensis* 'Jin Ou Fan Lv')

The unitig-level assembly was generated using hifiasm v0.24.0-r703 with default parameters, based on HiFi reads.

GPhase scaffolding. Hi-C reads were aligned to UTGs using Chromap v0.2.7-r494 with the parameter --remove-pcr-duplicates, and scaffolding was performed with the GPhase pipeline using the parameters --rescue --reassign_number 3.

HapHiC scaffolding. Hi-C reads were first aligned with BWA-MEM v0.7.18-r1243-dirty using the parameters -5SP, followed by BAM filtering with HapHiC's filter_bam.py. Scaffolding was then performed with the HapHiC v1.0.6 pipeline using the parameters --remove_allelic_links 3 --max_inflation 3.0 --RE "GATC" --correct_nrounds 2 --gfa <species>.p_utg.gfa.

ALLHiC scaffolding. An allele table was first constructed using GMAP (2023-07-20) with the annotated *OBDH* genome as reference. Hi-C reads were then aligned with BWA-MEM v0.7.18-r1243-dirty using the parameters -5SP, followed by BAM filtering with HapHiC's filter_bam.py. UTGs were partitioned with partition_gmap.py and scaffolded into chromosomal groups using the

ALLHiC v0.9.13 pipeline (https://github.com/tangerzhang/ALLHiC).

Assembly of the persimmon genome (*D. kaki Thunb* '*Xiaoguotianshi'*)

The unitig-level assembly was generated using hifiasm v0.24.0-r703 with default parameters, based on HiFi reads

GPhase scaffolding. Hi-C reads were aligned to UTGs using Chromap v0.2.7-r494 with the parameters --remove-pcr-duplicates -q 0, and scaffolding was performed with the GPhase pipeline using the parameters --rescue --reassign_number 1 --min_len 10.

Assembly of the alfalfa genome (*M. sativa* 'X*injiangdaye'*)

The unitig-level assembly was generated using hifiasm v0.24.0-r703 with default parameters, based on HiFi reads

GPhase scaffolding. Hi-C reads were aligned to UTGs using Chromap v0.2.7-r494 with the parameters --remove-pcr-duplicates -q 0, and scaffolding was performed with the GPhase pipeline using the parameters --rescue --reassign_number 1 --min_len 200.

Assembly of the jujube genome (*Z. mauritiana* '*Xishuangyesheng'*)

The unitig-level assembly was generated using hifiasm v0.24.0-r703 with default parameters, based on HiFi reads

GPhase scaffolding. Hi-C reads were aligned to UTGs using Chromap v0.2.7-r494 with the parameters --remove-pcr-duplicates -q 0, and scaffolding was performed with the GPhase pipeline using the parameters --rescue --reassign_number 1 --min_len 200.

Assembly of the alfalfa genome (*M. sativa* 'Zhongmu-4')

The unitig-level assembly was generated using hifiasm v0.24.0-r703 with default parameters, integrating both HiFi and ONT reads.

GPhase Scaffolding. Pore-C data were aligned to unitigs and converted to pairs format using PPL-Toolbox with default parameters. Scaffolding was then performed using GPhase with the parameters --rescue --reassign_number 1 --min_len 10.

Supplementary Note 4: Details of sequencing data simulation

Here we detail the HiFi and Hi-C data simulation process for the four fully simulated tetraploid genomes with varying degrees of sequence diversity.

HiFi reads were simulated using Badread v0.4.1. The reference genome was segmented into chromosomes using SeqKit[21] v2.10.1. For each chromosome, a 90× coverage depth was simulated using the parameters --quantity 90x --error_model pacbio2021 --qscore_model pacbio2021 --identity 30,3 --length 25000,3000 --junk_reads 0 --random_reads 0 --glitches 0,0,0 --start_adapter_seq "" --end_adapter_seq "".

Hi-C data were simulated using sim3C v0.7.1. Using the parameters --dist uniform -n 257140452 -l 150 -e DpnII -m hic, a total of 257,140,452 paired reads of 150 bp each were generated.

References

1. Wick, R.R., Schultz, M.B., Zobel, J. & Holt, K.E. Bandage: interactive visualization of de novo genome assemblies. *Bioinformatics* **31**, 3350-2 (2015).
2. Alonge, M. *et al.* Automated assembly scaffolding using RagTag elevates a new tomato system for high-throughput genome editing. *Genome Biol* **23**, 258 (2022).
3. Durand, N.C. *et al.* Juicer provides a one-click system for analyzing loop-resolution Hi-C experiments. **3**, 95-98 (2016).
4. Dudchenko, O. *et al.* De novo assembly of the Aedes aegypti genome using Hi-C yields chromosome-length scaffolds. **356**, 92-95 (2017).
5. Durand, N.C. *et al.* Juicebox Provides a Visualization System for Hi-C Contact Maps with Unlimited Zoom. *Cell Syst* **3**, 99-101 (2016).
6. Wick, R.R. Badread: simulation of error-prone long reads. *Journal of Open Source Software* **4**, 1316 (2019).
7. DeMaere, M.Z. & Darling, A.E. Sim3C: simulation of Hi-C and Meta3C proximity ligation sequencing technologies. *GigaScience* **7**, gix103 (2018).
8. Marçais, G. & Kingsford, C.J.B. A fast, lock-free approach for efficient parallel counting of occurrences of k-mers. **27**, 764-770 (2011).
9. Ranallo-Benavidez, T.R., Jaron, K.S. & Schatz, M.C. GenomeScope 2.0 and Smudgeplot for reference-free profiling of polyploid genomes. Nat. Commun. 11: 1432. (2020).
10. Chin, C.-S. & Khalak, A.J.B. Human genome assembly in 100 minutes. 705616 (2019).
11. Xu, M. *et al.* TGS-GapCloser: A fast and accurate gap closer for large genomes with low coverage of error-prone long reads. *Gigascience* **9**(2020).
12. Tang, H. *et al.* JCVI: A versatile toolkit for comparative genomics analysis. *Imeta* **3**, e211 (2024).
13. Hibrand Saint-Oyant, L. *et al.* A high-quality genome sequence of Rosa chinensis to elucidate ornamental traits. *Nature Plants* **4**, 473-484 (2018).
14. Bao, Z. *et al.* Genome architecture and tetrasomic inheritance of autotetraploid potato. *Molecular Plant* **15**, 1211-1226 (2022).
15. Marçais, G. *et al.* MUMmer4: A fast and versatile genome alignment system. *PLoS computational biology* **14**, e1005944 (2018).
16. Rhie, A., Walenz, B.P., Koren, S. & Phillippy, A.M. Merqury: reference-free quality, completeness, and phasing assessment for genome assemblies. *Genome Biol* **21**, 245 (2020).
17. Xu, Z. & Wang, H. LTR_FINDER: an efficient tool for the prediction of full-length LTR retrotransposons. *Nucleic Acids Res* **35**, W265-8 (2007).
18. Ou, S. & Jiang, N. LTR_retriever: A Highly Accurate and Sensitive Program for Identification of Long Terminal Repeat Retrotransposons. *Plant Physiol* **176**, 1410-1422 (2018).
19. Goel, M., Sun, H., Jiao, W.B. & Schneeberger, K. SyRI: finding genomic rearrangements and local sequence differences from whole-genome assemblies. *Genome Biol* **20**, 277 (2019).
20. Wu, T.D. & Watanabe, C.K. GMAP: a genomic mapping and alignment program for mRNA and EST sequences. *Bioinformatics* **21**, 1859-1875 (2005).
21. Shen, W., Sipos, B. & Zhao, L. SeqKit2: A Swiss army knife for sequence and alignment processing. *Imeta* **3**, e191 (2024).

Supplementary Figures

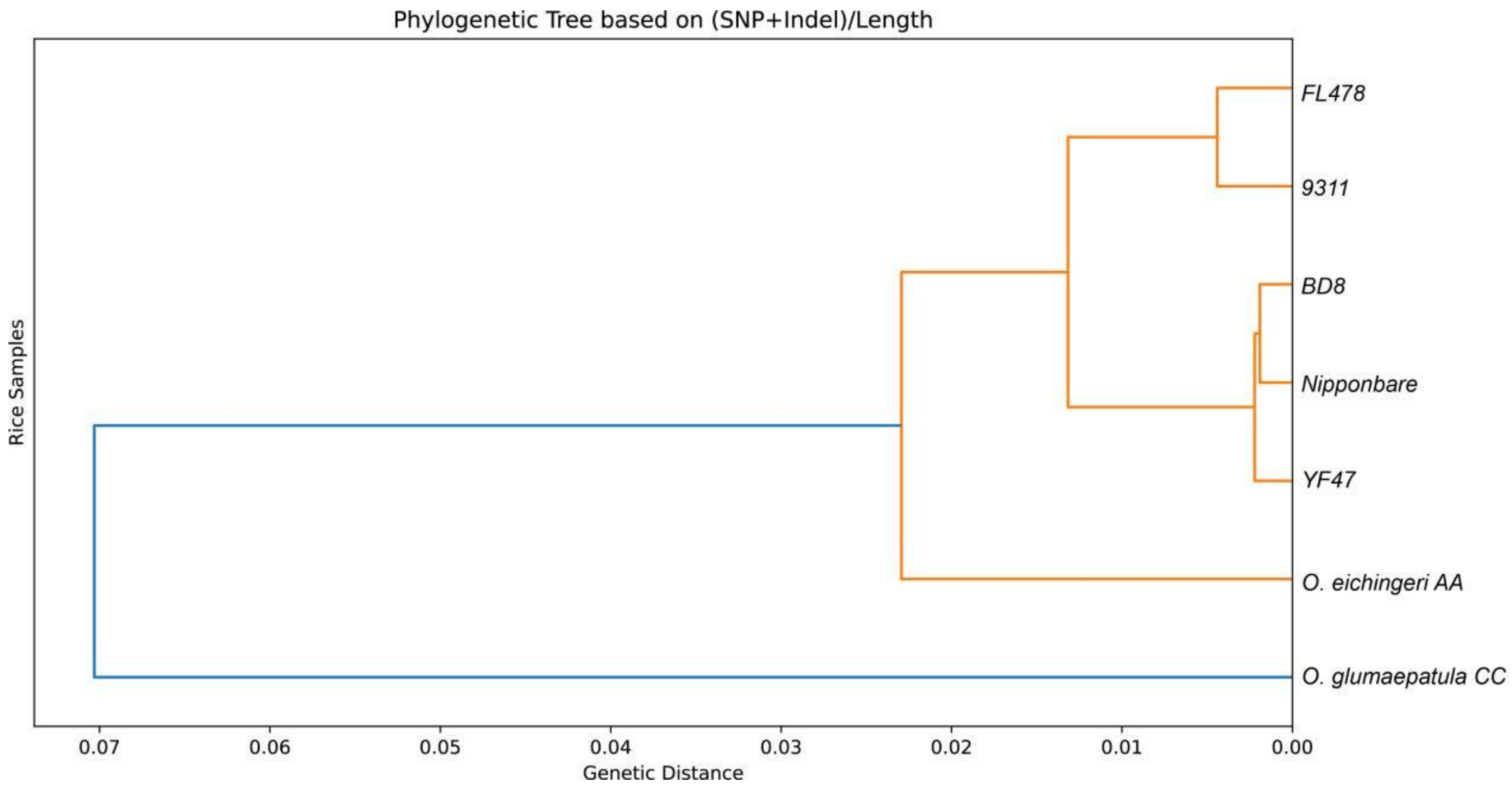

Supplementary Fig. 1: Phylogenetic tree of the five rice genomes used to construct the semi-simulated tetraploid and triploid genomes.

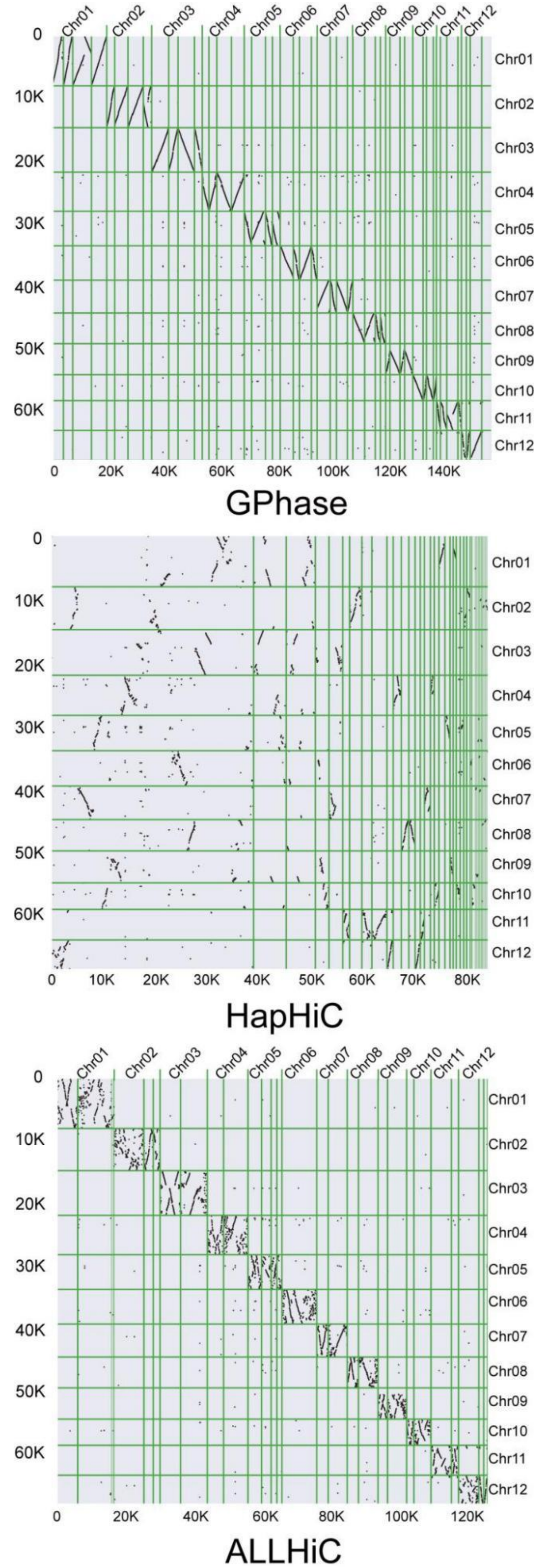

Supplementary Fig. 2: Gene-based synteny plots of the semi-simulated tetraploid genome assemblies generated by the three tools.

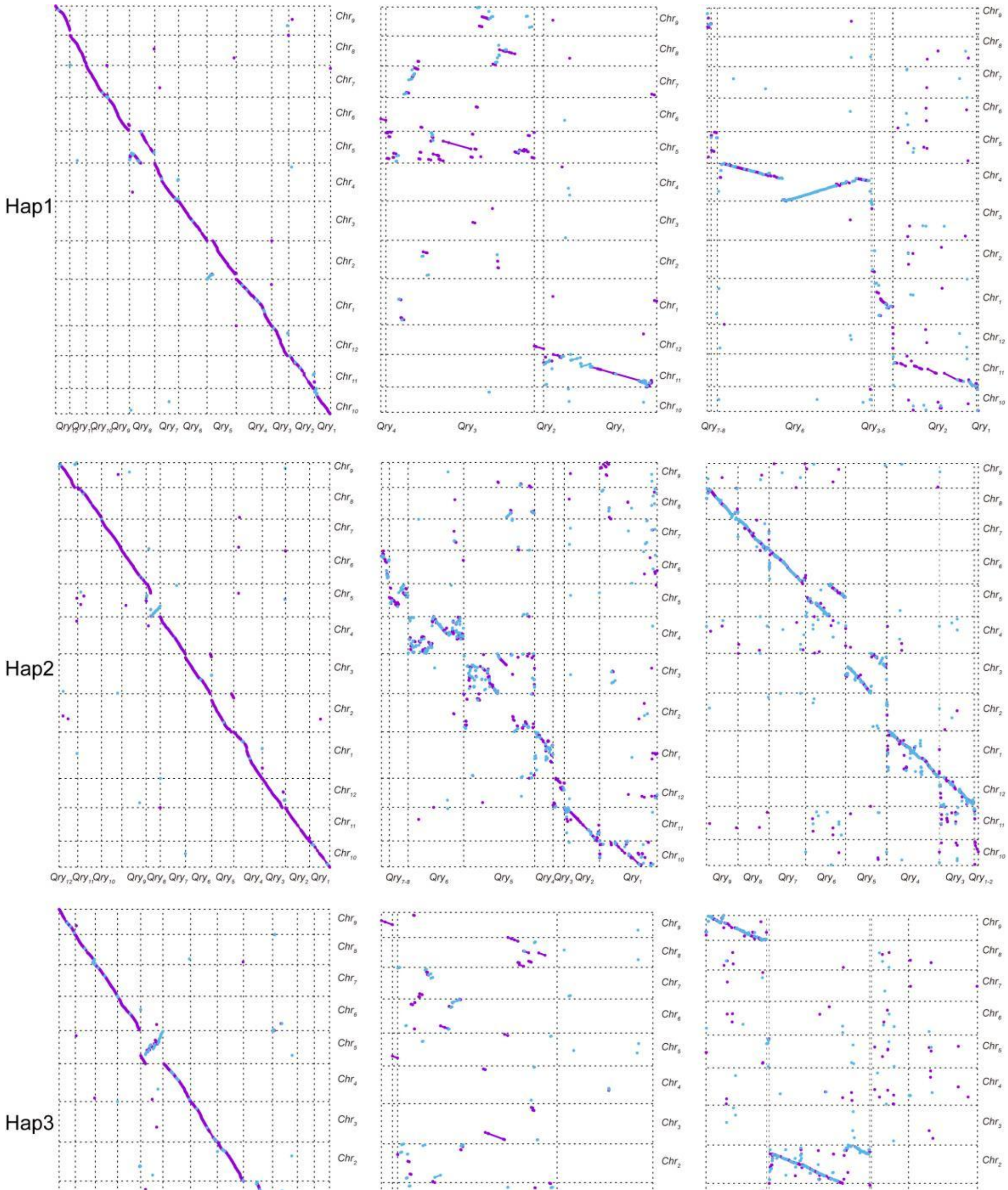

Supplementary Fig. 3: Sequence-alignment-based synteny plots of the semi-simulated triploid genome assemblies generated by the three tools.

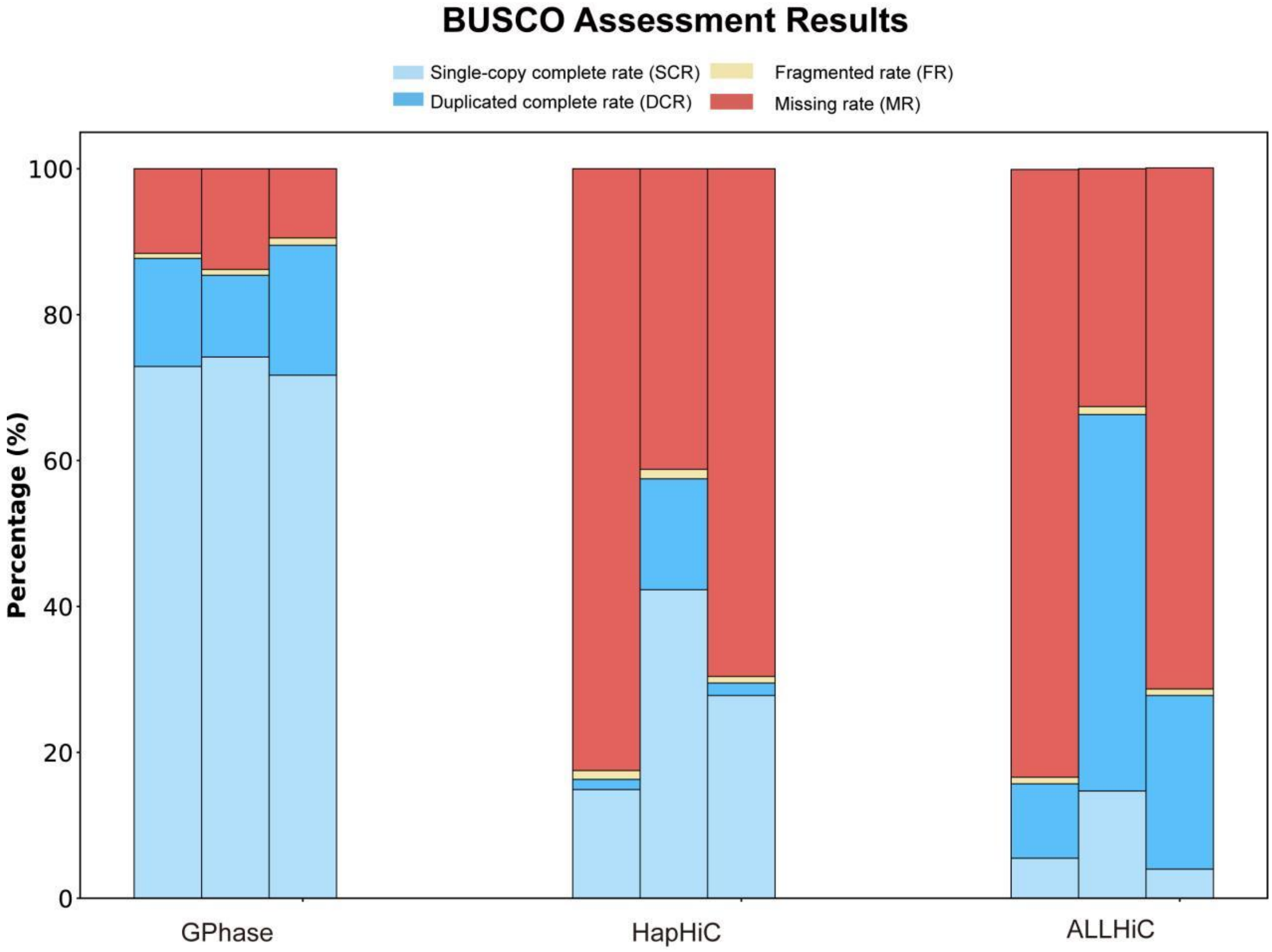

Supplementary Fig. 4: BUSCO completeness scores of the semi-simulated triploid genome assemblies generated by the three tools. Each bar represents one haplotype.

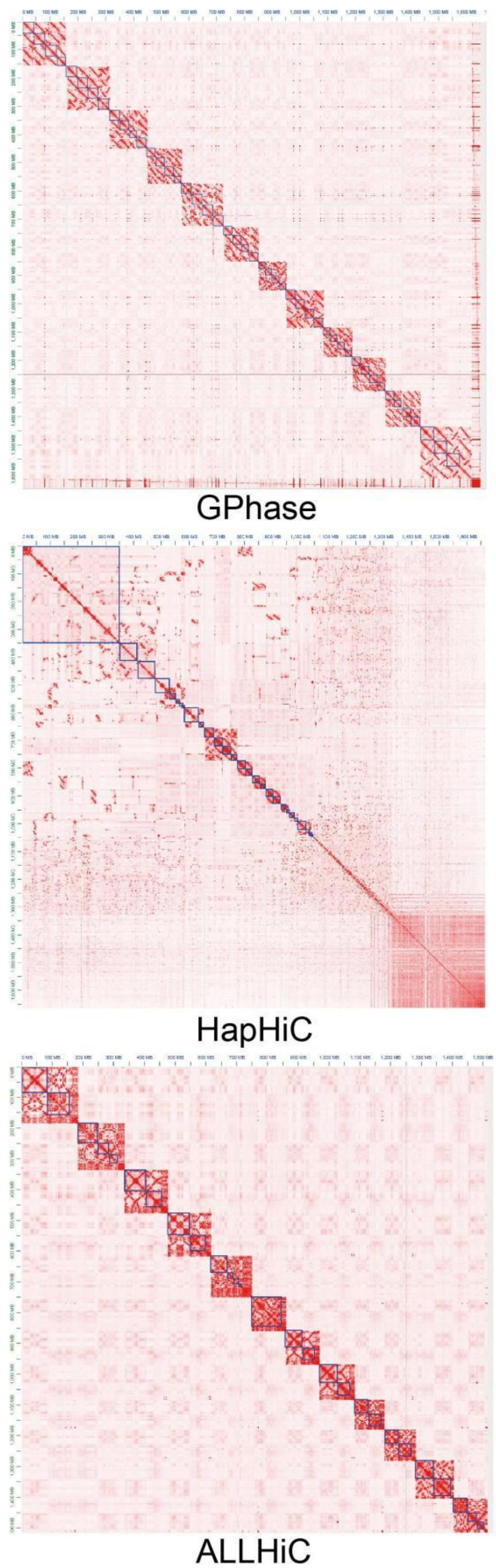

Supplementary Fig. 5: Hi-C contact maps of the semi-simulated tetraploid genome assemblies generated by the three tools.

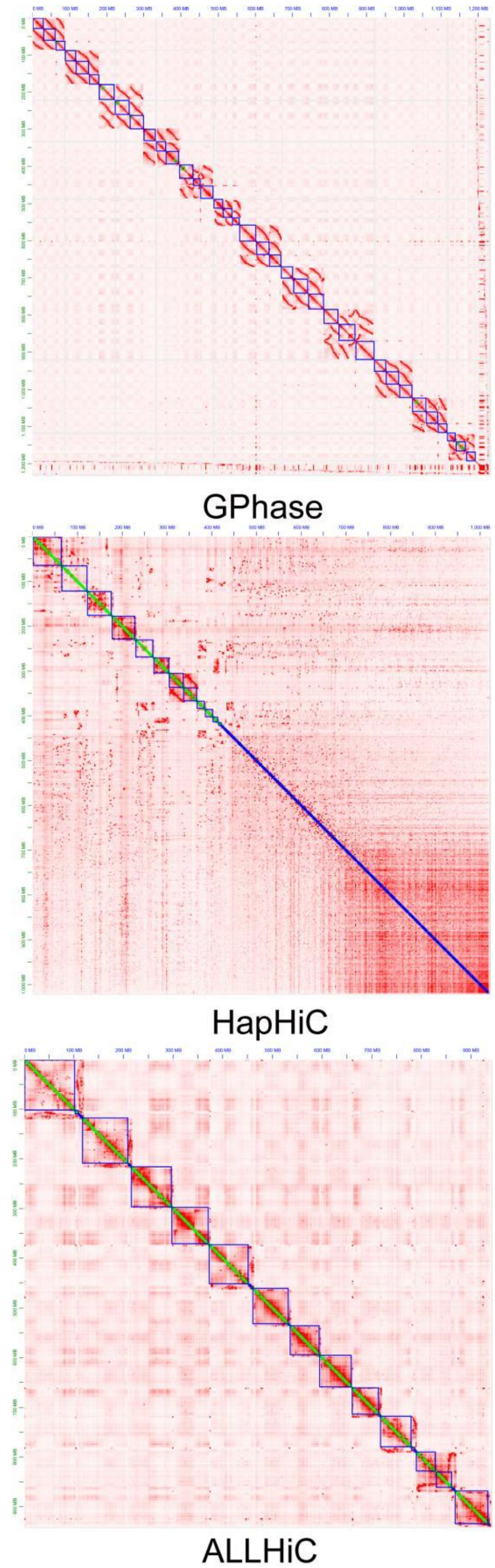

Supplementary Fig. 6: Hi-C contact maps of the semi-simulated triploid genome assemblies generated by the three tools.

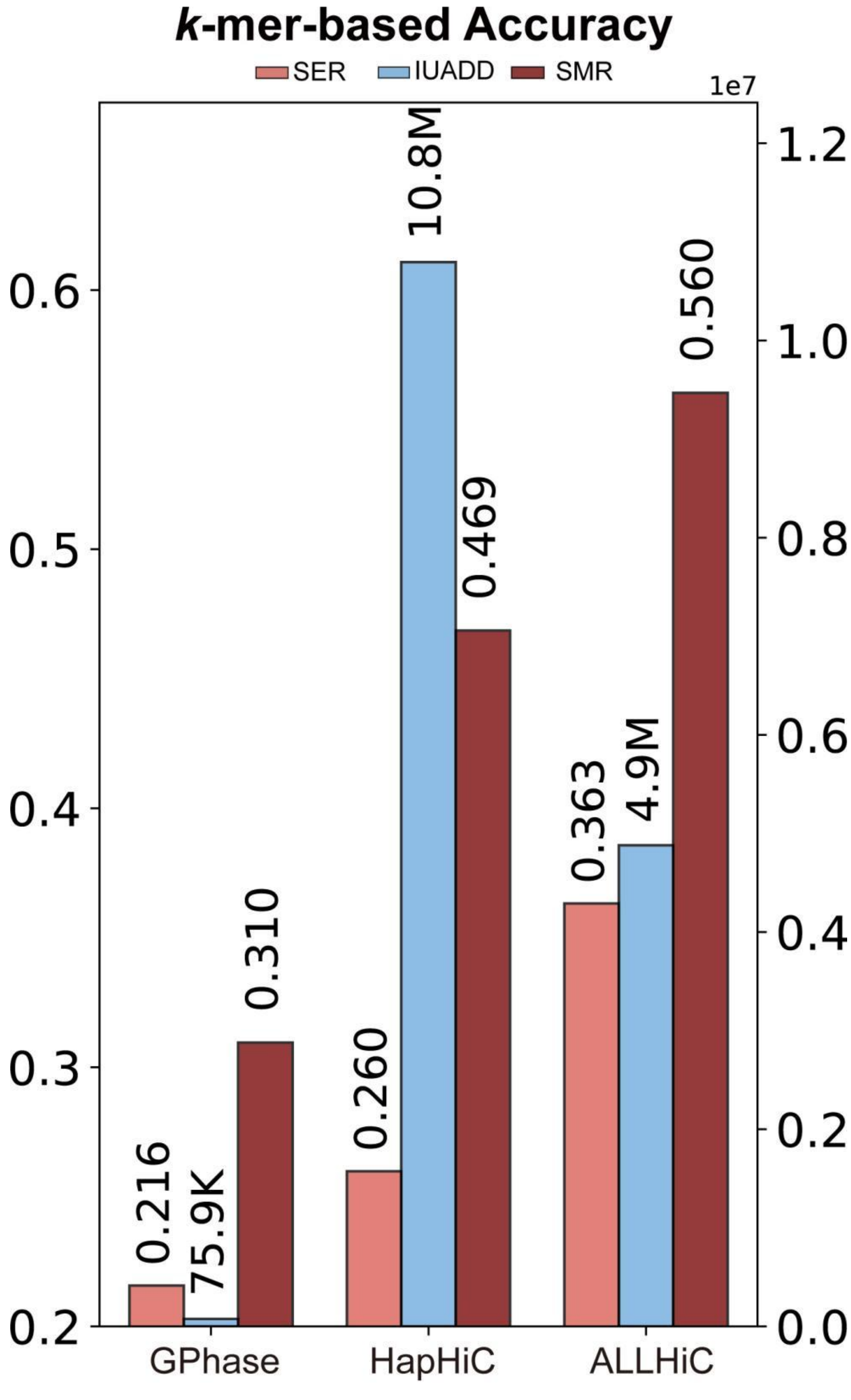

Supplementary Fig. 7: *k*-mer-based assessment scores of the semi-simulated triploid genome assemblies generated by the three tools: Sequence Error Rate (SER), Sequence Missing Rate (SMR), and Inter-unitig Average Distance Difference (IUADD), with lower values indicating higher accuracy.

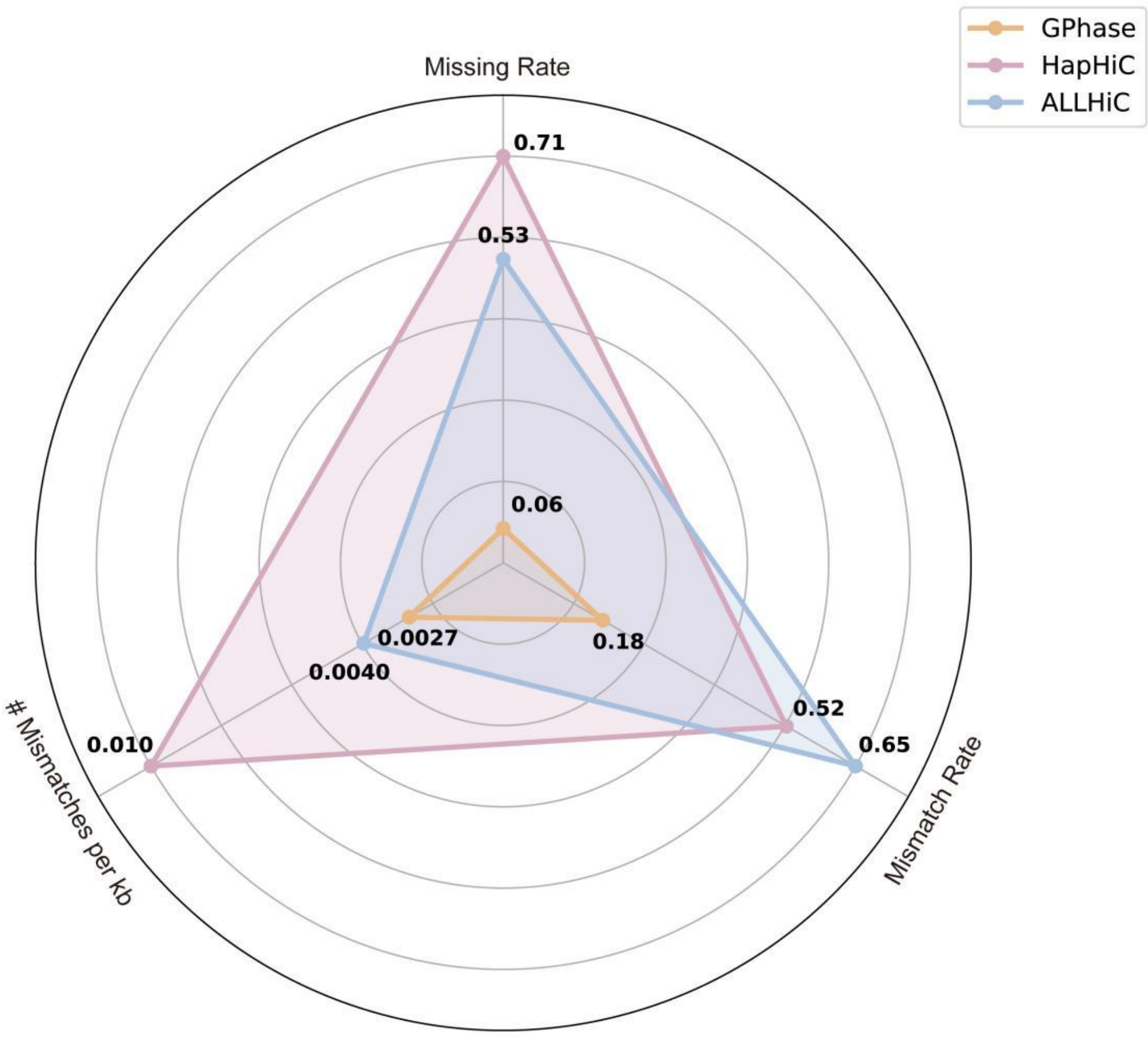

Supplementary Fig. 8: Sequence-alignment-based assessment scores of the semi-simulated triploid genome assemblies generated by the three tools: Missing Rate, Mismatch Rate, and #Mismatches per kb with the lower values indicate higher accuracy.

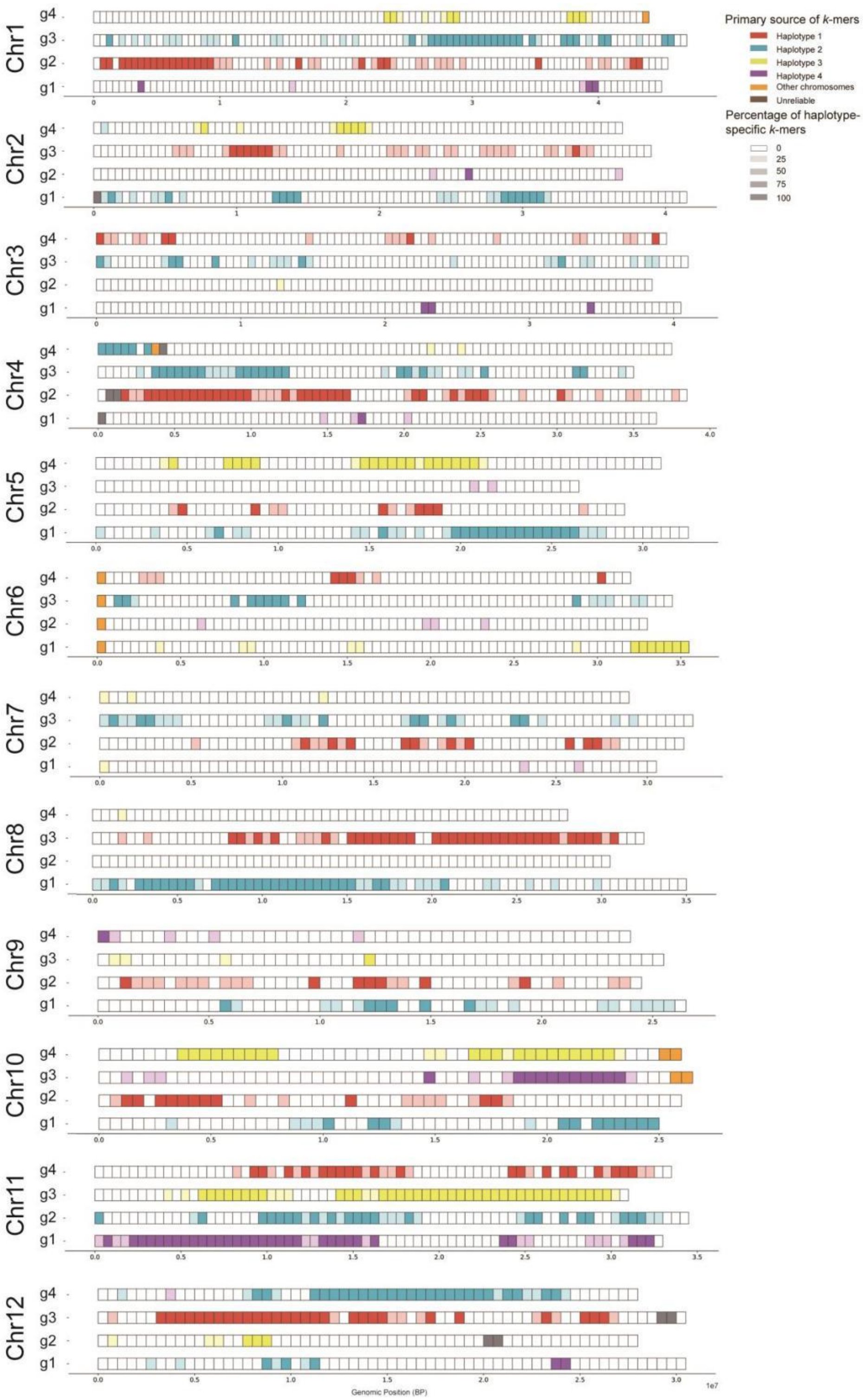

Supplementary Fig. 9: *k*-mer distribution plot of GPhase assembly for the simulated tetraploid genome. Each of the four rows represents a haplotype, and each bin corresponds to a 500 kb region of that haplotype. The colour indicates the haplotype origin: red, blue, yellow, and purple represent bins in which the majority of *k*-mers originate from haplotypes 1, 2, 3, and 4 of the ground-truth genome, respectively. White bins represent regions dominated by *k*-mers shared among multiple haplotypes in the ground truth; these regions cannot be reliably evaluated using this visualization approach.

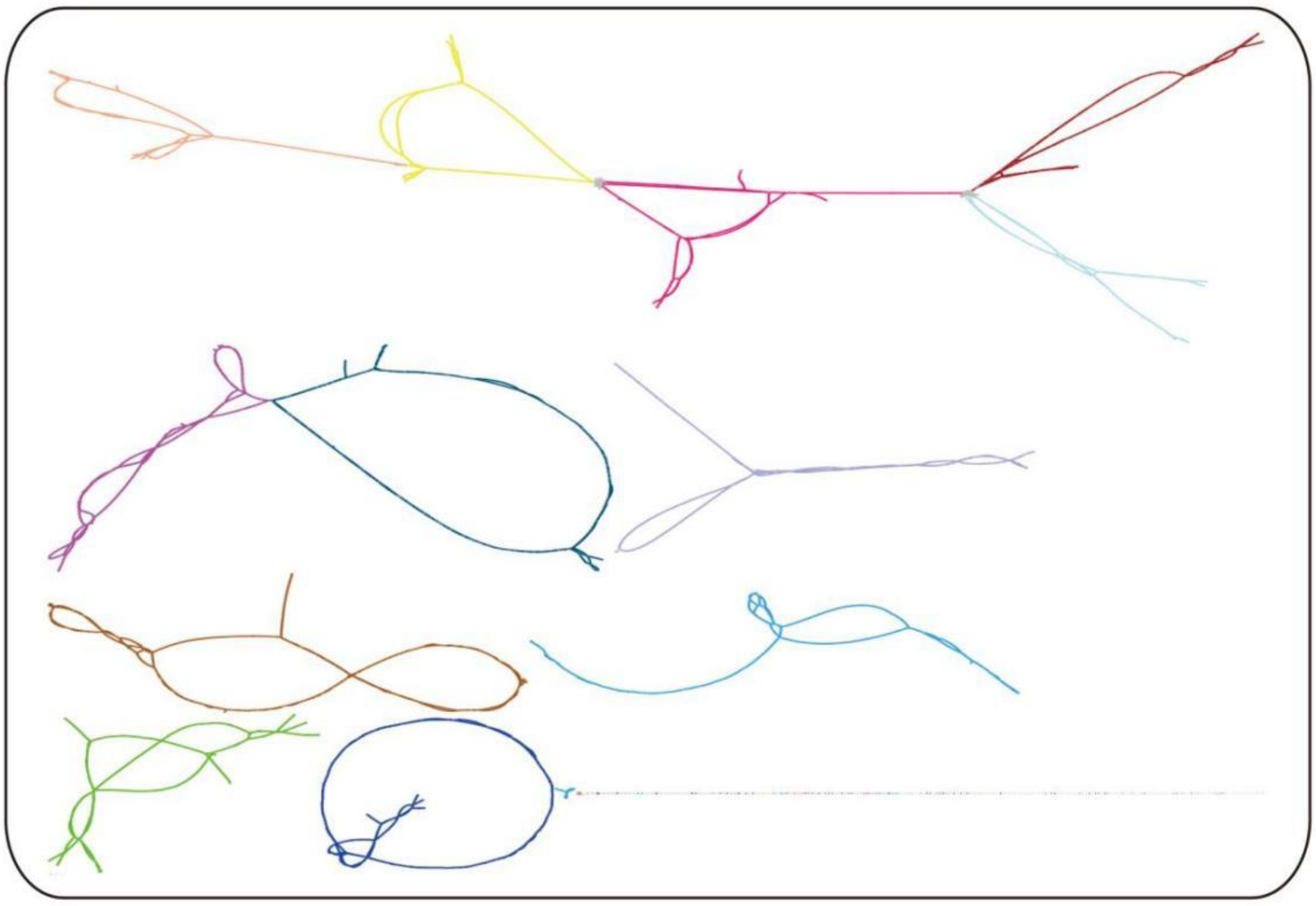

ground truth

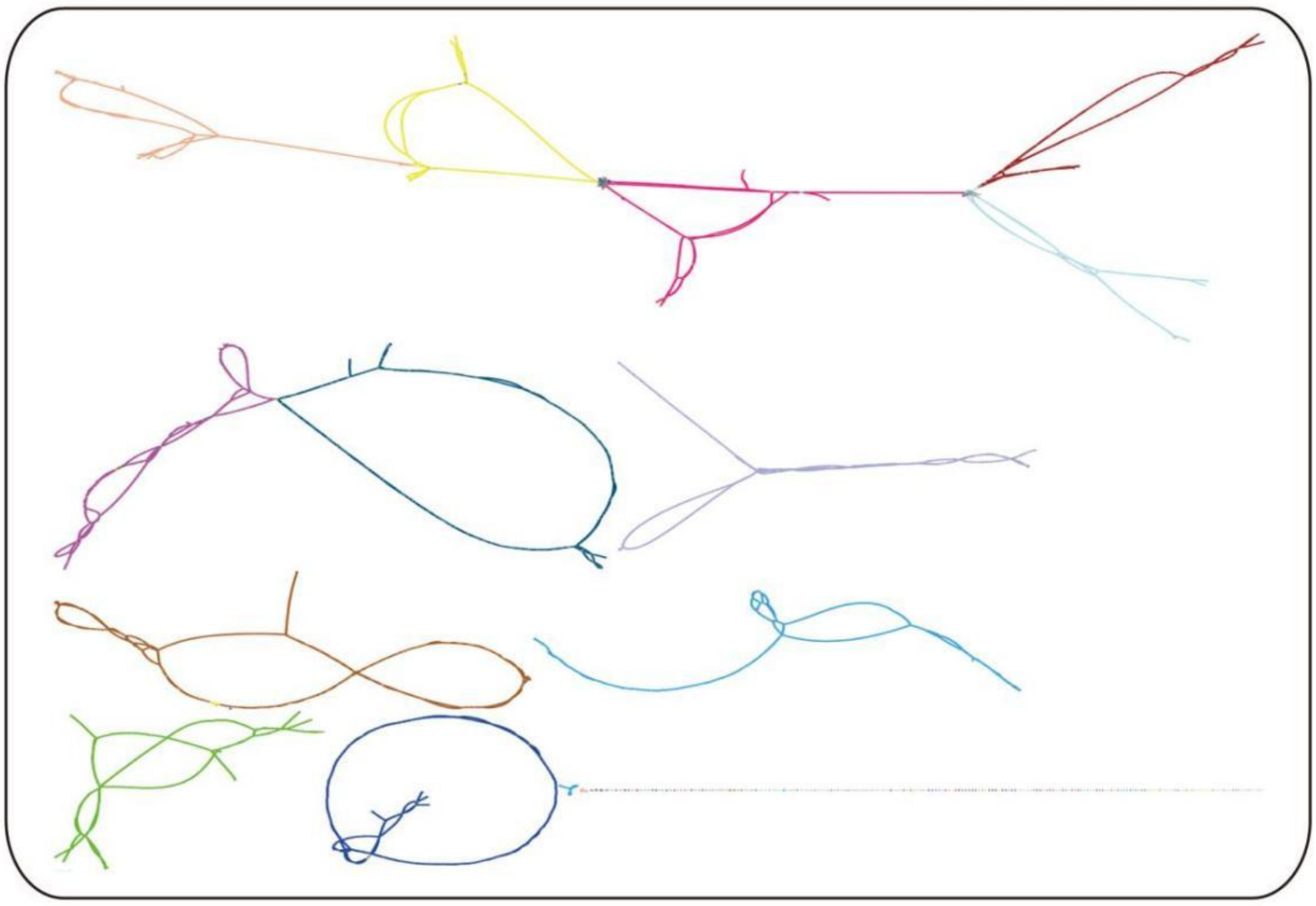

GPhase

Supplementary Fig. 10: Bandage visualization of unitig distributions across 12 chromosomes in the ground truth (top) and in the GPhase chromosome partitioning results (bottom; Step 1 of the GPhase algorithm) from the stepwise performance evaluation on the simulated autotetraploid genome. Distinct colors denote different chromosomes.

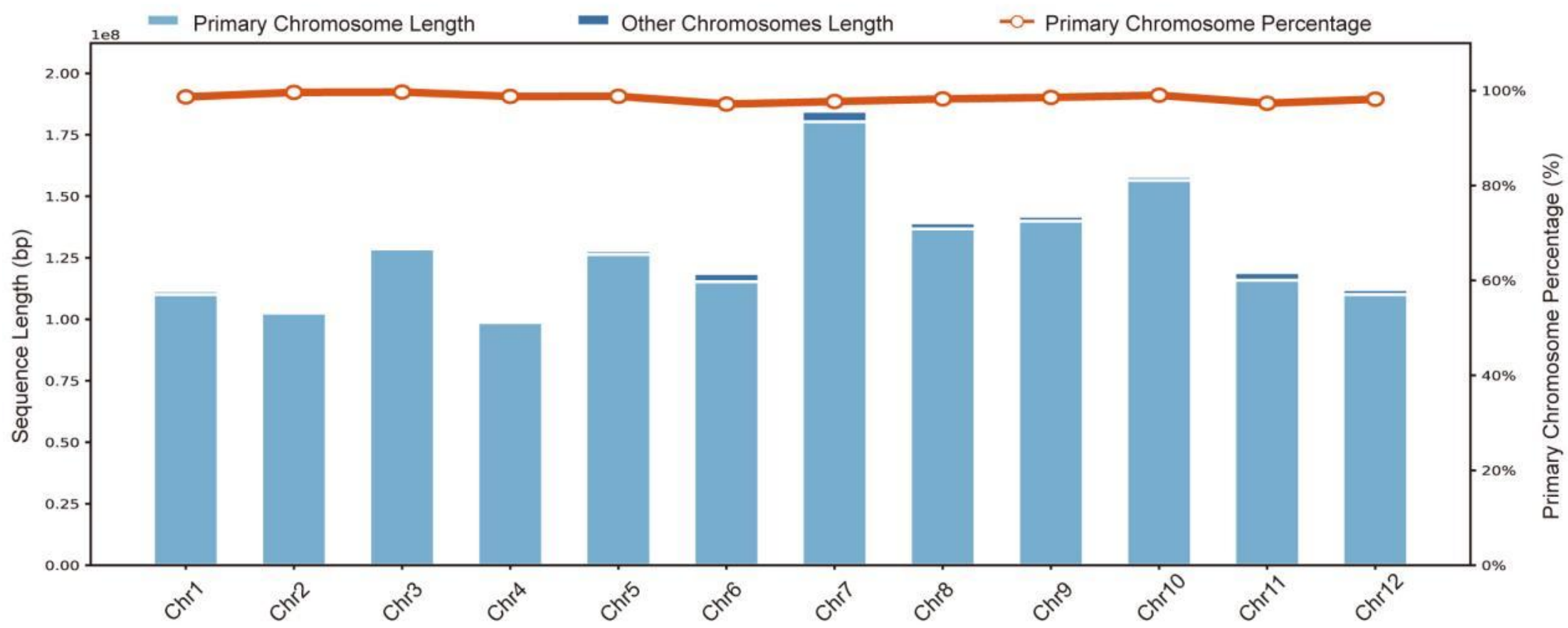

Supplementary Fig. 11: Total length and length proportion of unitigs correctly assigned to each chromosome during chromosome partitioning by GPhase (Step 1 of the GPhase algorithm) in the stepwise performance evaluation on the simulated autotetraploid genome. Light blue bars ("Primary Chromosome Length") indicate the total length of unitigs correctly assigned to the corresponding chromosome, whereas dark blue bars ("Other Chromosome Length") represent the total length of unitigs incorrectly assigned. Orange line segments ("Primary Chromosome Percentage") denote the proportion of unitig length correctly assigned to each chromosome.

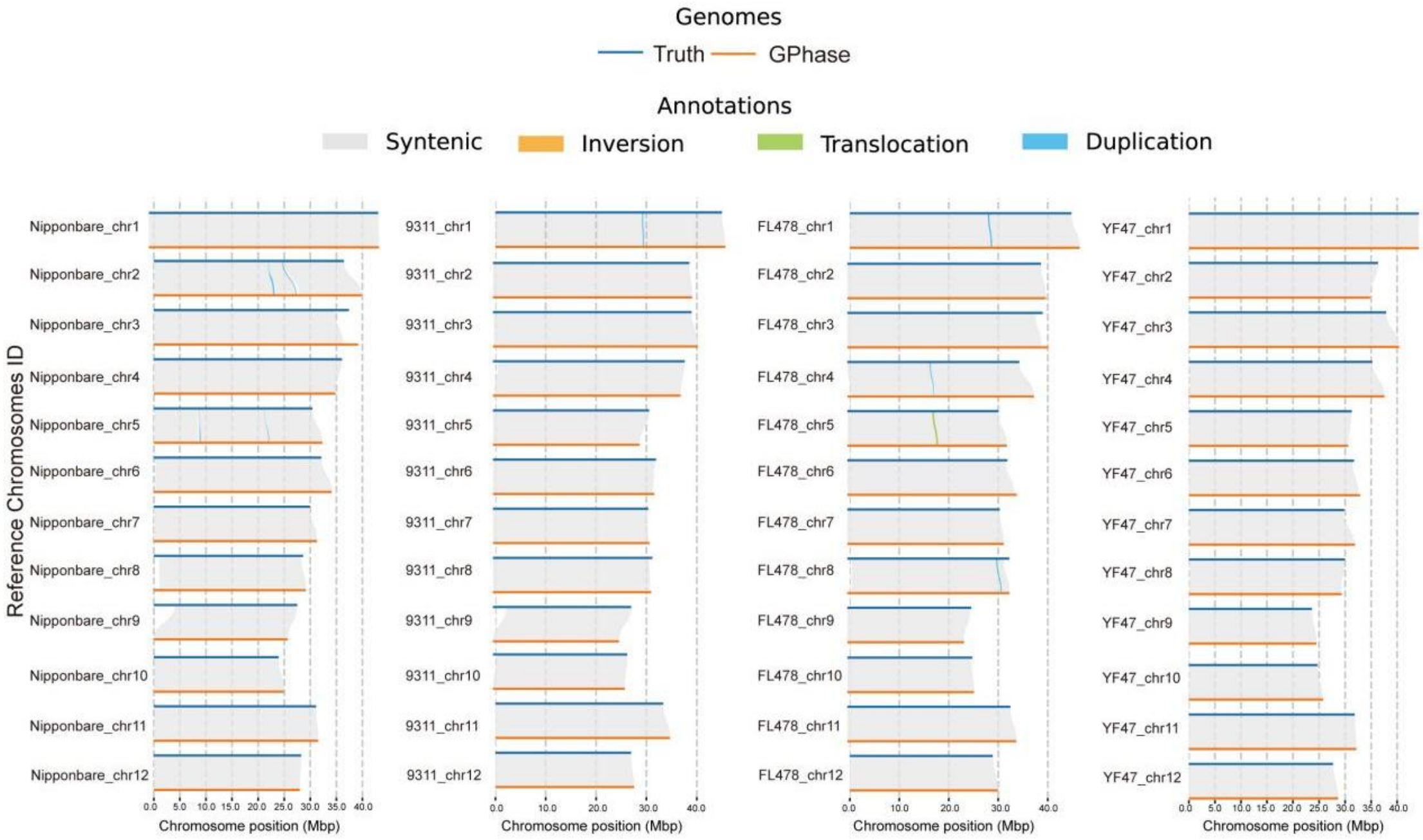

Supplementary Fig. 12: Validation of haplotype phasing (Step 2 of GPhase algorithm) in the Stepwise performance evaluation of GPhase on the simulated autotetraploid genome. Synteny plots between the ground truth and the assemblies from reference-based scaffolding of unitigs from haplotype phasing process. The ground truth chromosome sequences were used as reference.

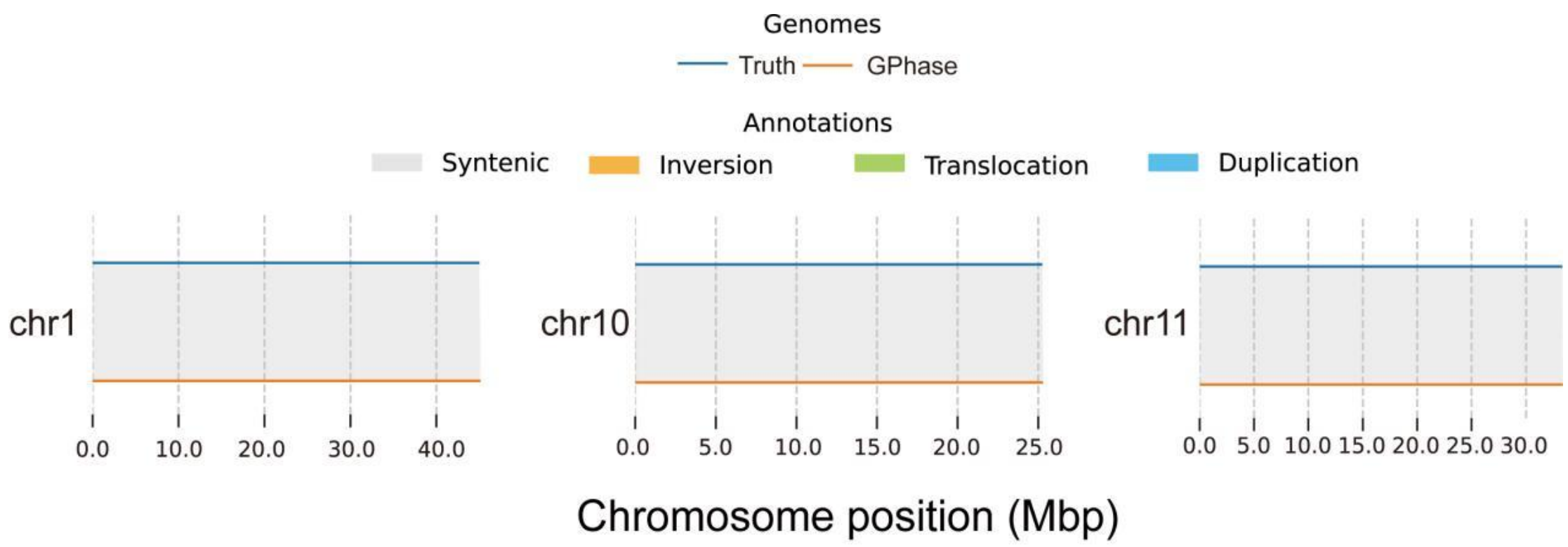

Supplementary Fig. 13: Validation of scaffolding (Step 3 of GPhase algorithm) in the stepwise performance evaluation of GPhase on the simulated autotetraploid genome. Synteny plots between the assemblies from GPhase scaffolding and the ground truth for three haplotypes on chromosome 1, 10, and 11.

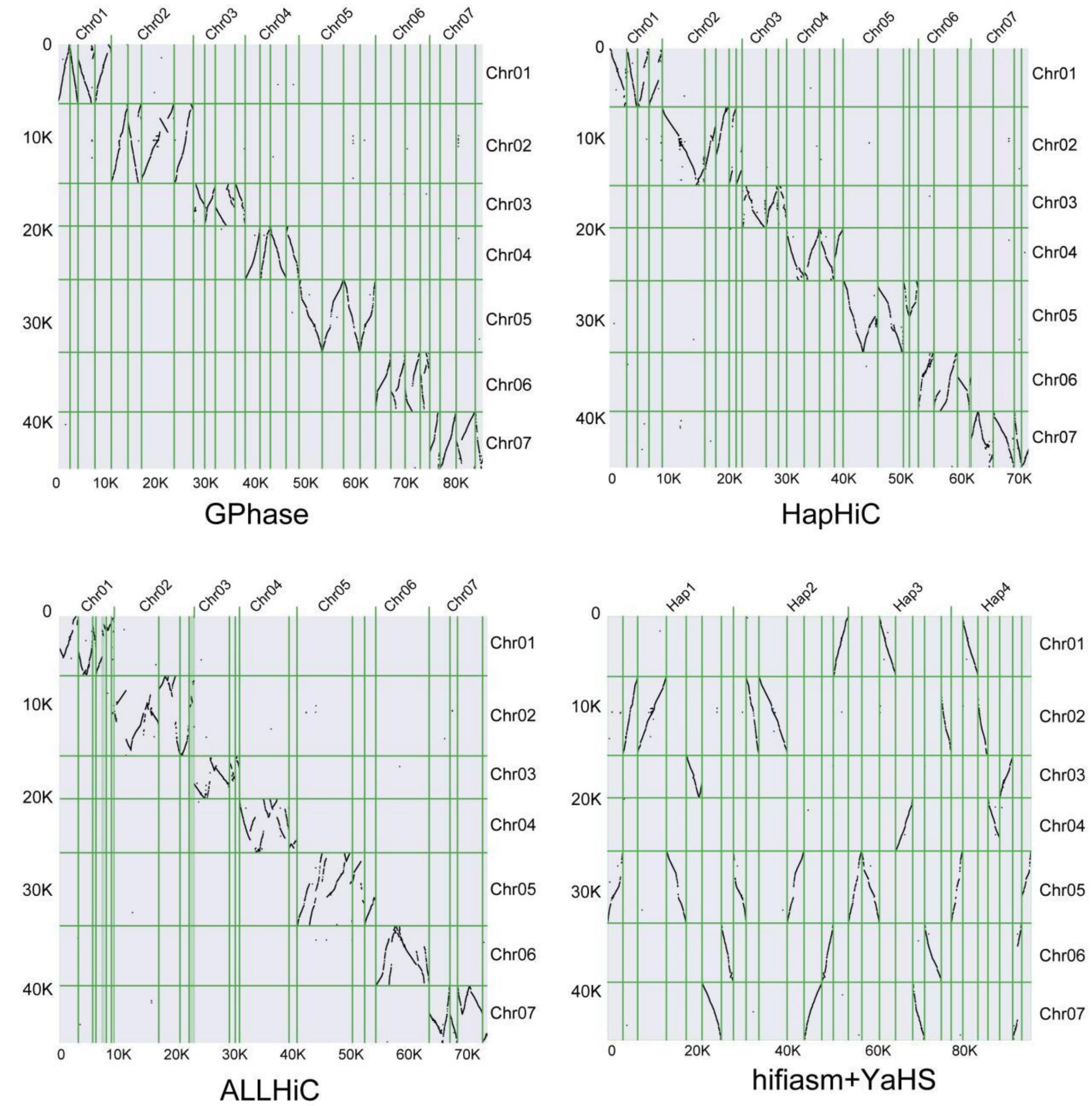

Supplementary Fig. 14: Gene-based synteny plots of the rose (*R. hybrida* ‘Samantha’) genome assemblies generated by the four methods using Hi-C data.

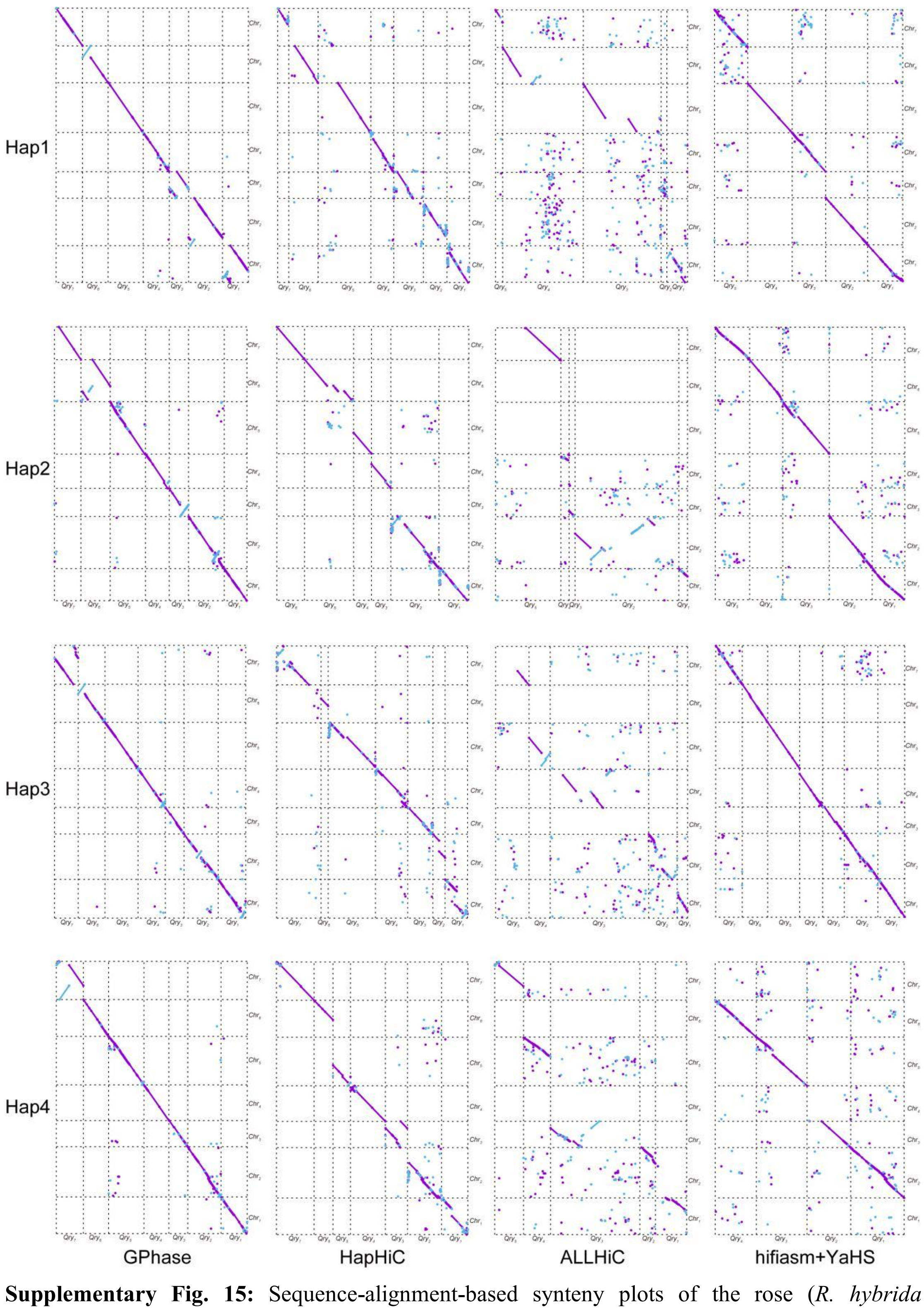

Supplementary Fig. 15: Sequence-alignment-based synteny plots of the rose (*R. hybrida* 'Samantha') genome assemblies generated by the four methods using Hi-C data.

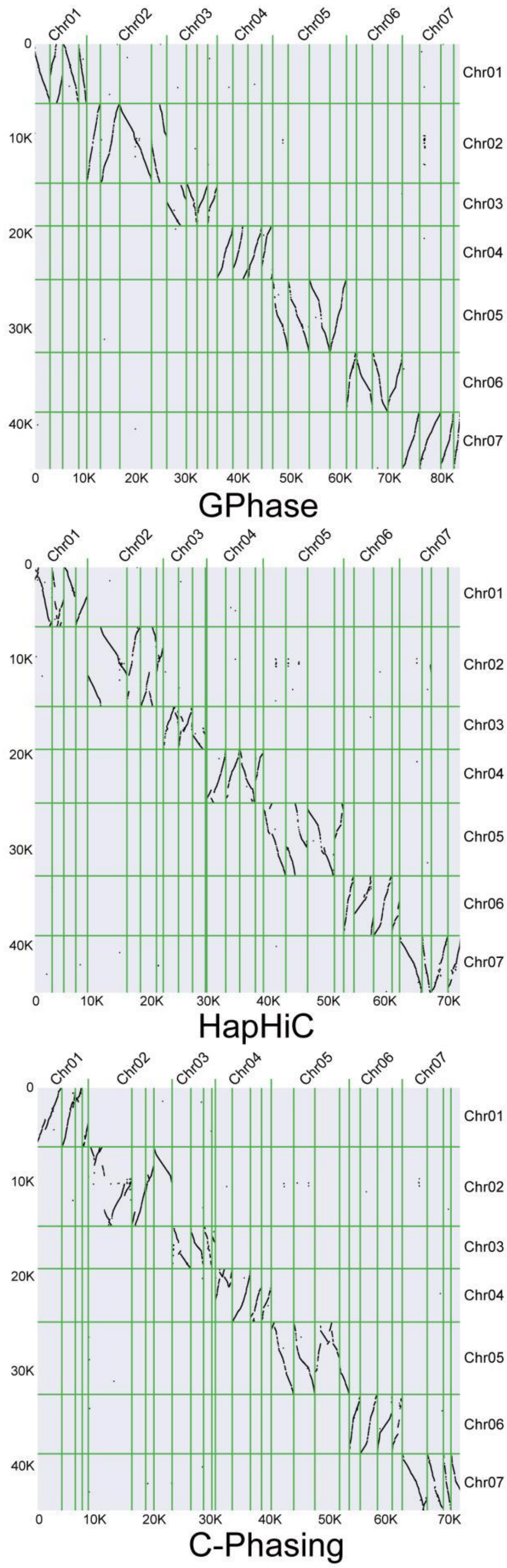

Supplementary Fig. 16: Gene-based synteny plots of the rose (*R. hybrida* 'Samantha') genome assemblies generated by the three tools using Pore-C data.

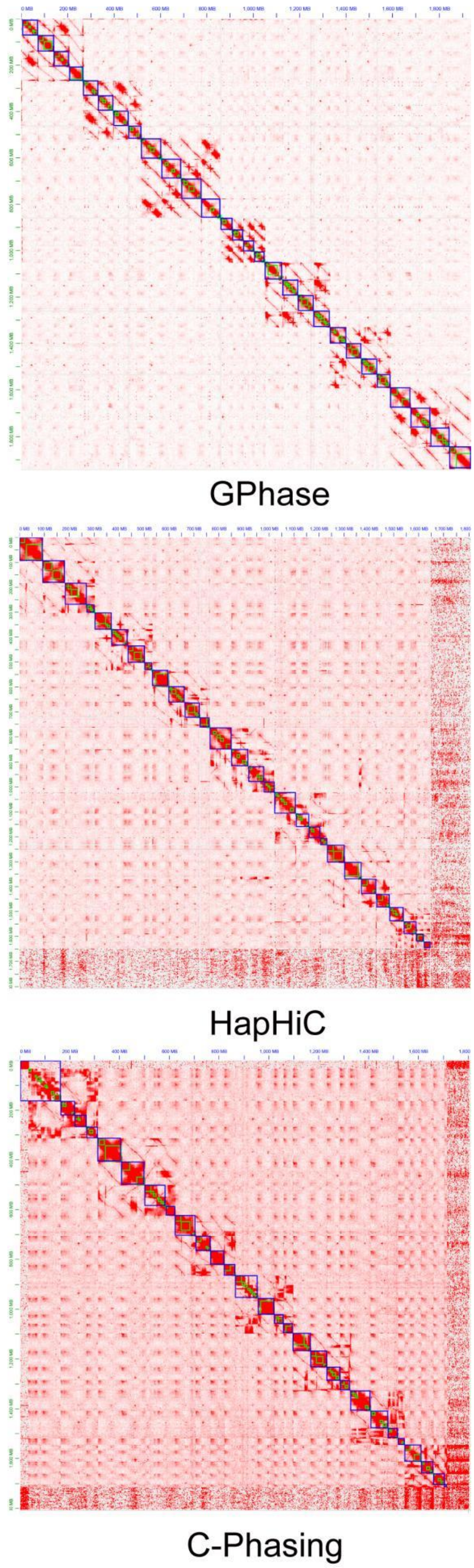

Supplementary Fig. 17: Pore-C contact maps of the rose (*R. hybrida* ‘Samantha’) genome assemblies generated by the three tools using Pore-C data.

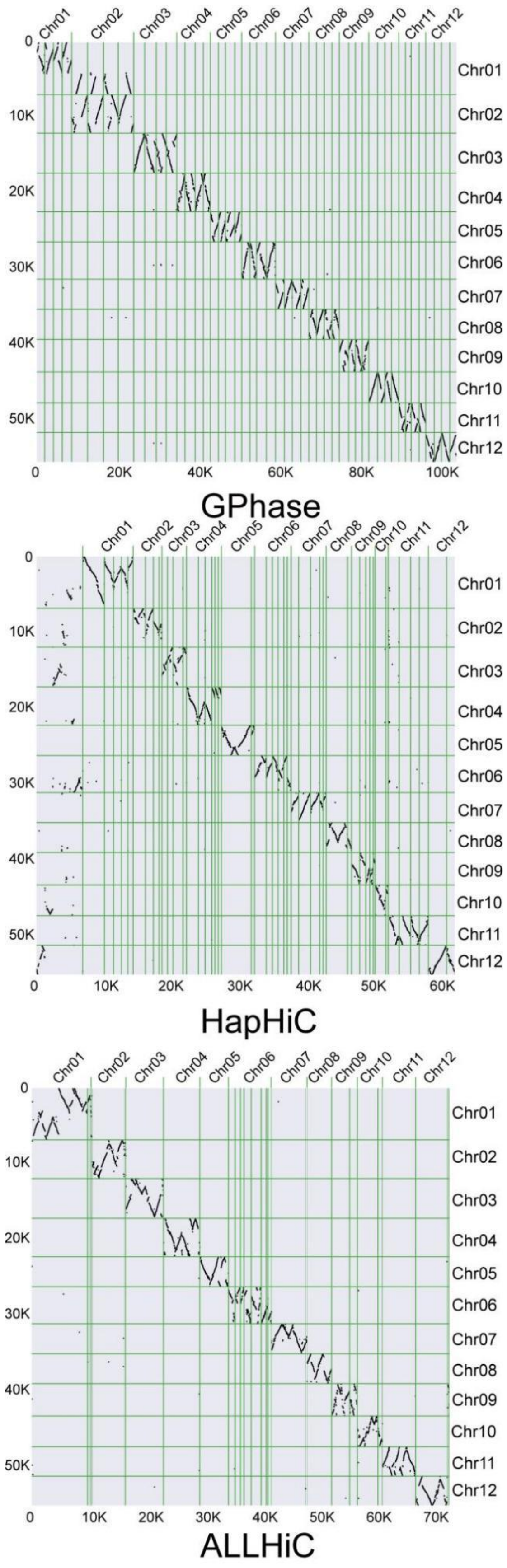

Supplementary Fig. 18: Gene-based synteny plots of the potato (*S. tuberosum* 'Otava') genome assemblies generated by the three tools.

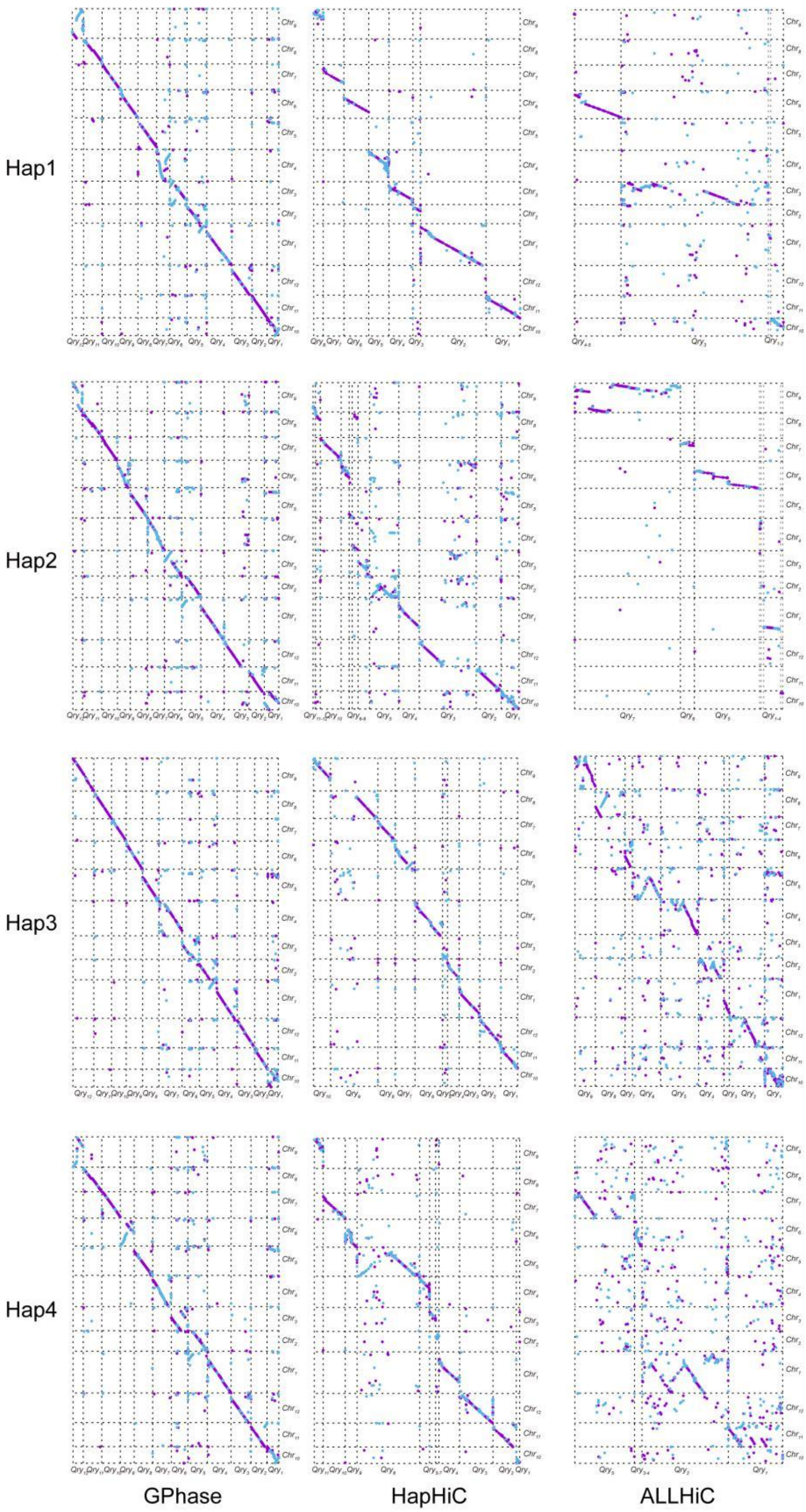

Supplementary Fig. 19: Sequence-alignment-based synteny plots of the potato (*S. tuberosum* 'Otava') genome assemblies generated by the three tools.

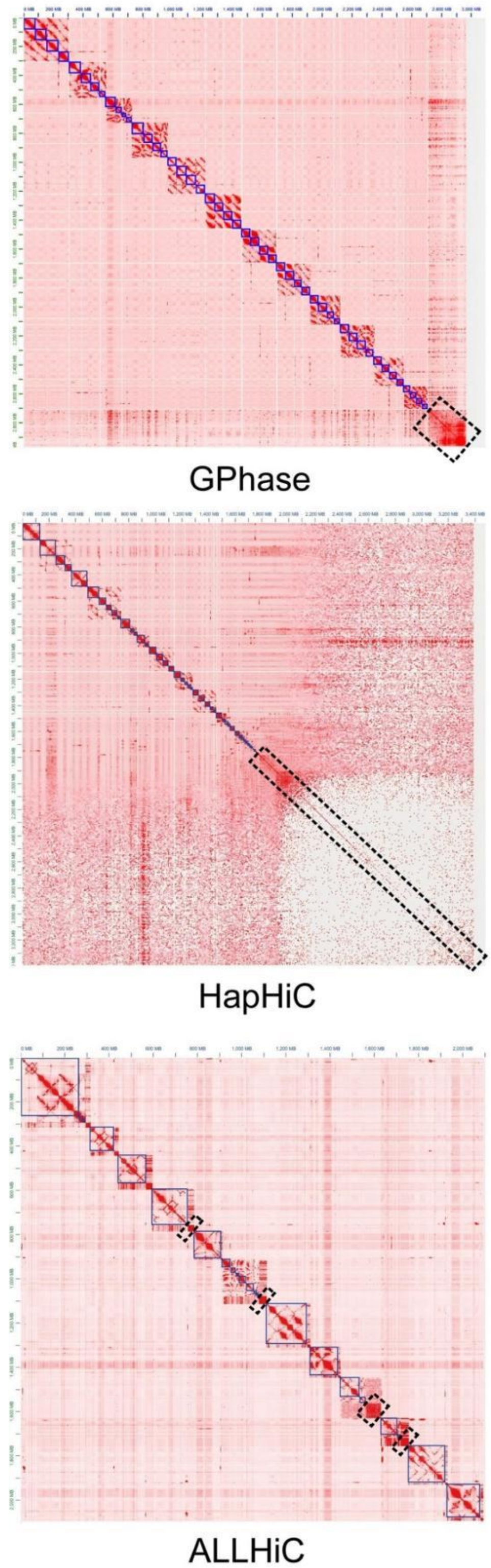

Supplementary Fig. 20: Hi-C contact maps of the potato (*S. tuberosum* 'Otava') genome assemblies generated by the three tools.Black dashed boxes highlight genomic regions that failed to be scaffolded by each tool. Unlike the other two methods, where all unresolved sequences are clustered at the far end of the Hi-C map, ALLHiC does not exclude unplaced sequences from chromosomes; rather, it attaches sequences with ambiguous phasing to the chromosomal termini (several of the most prominent such regions are indicated with dashed boxes).

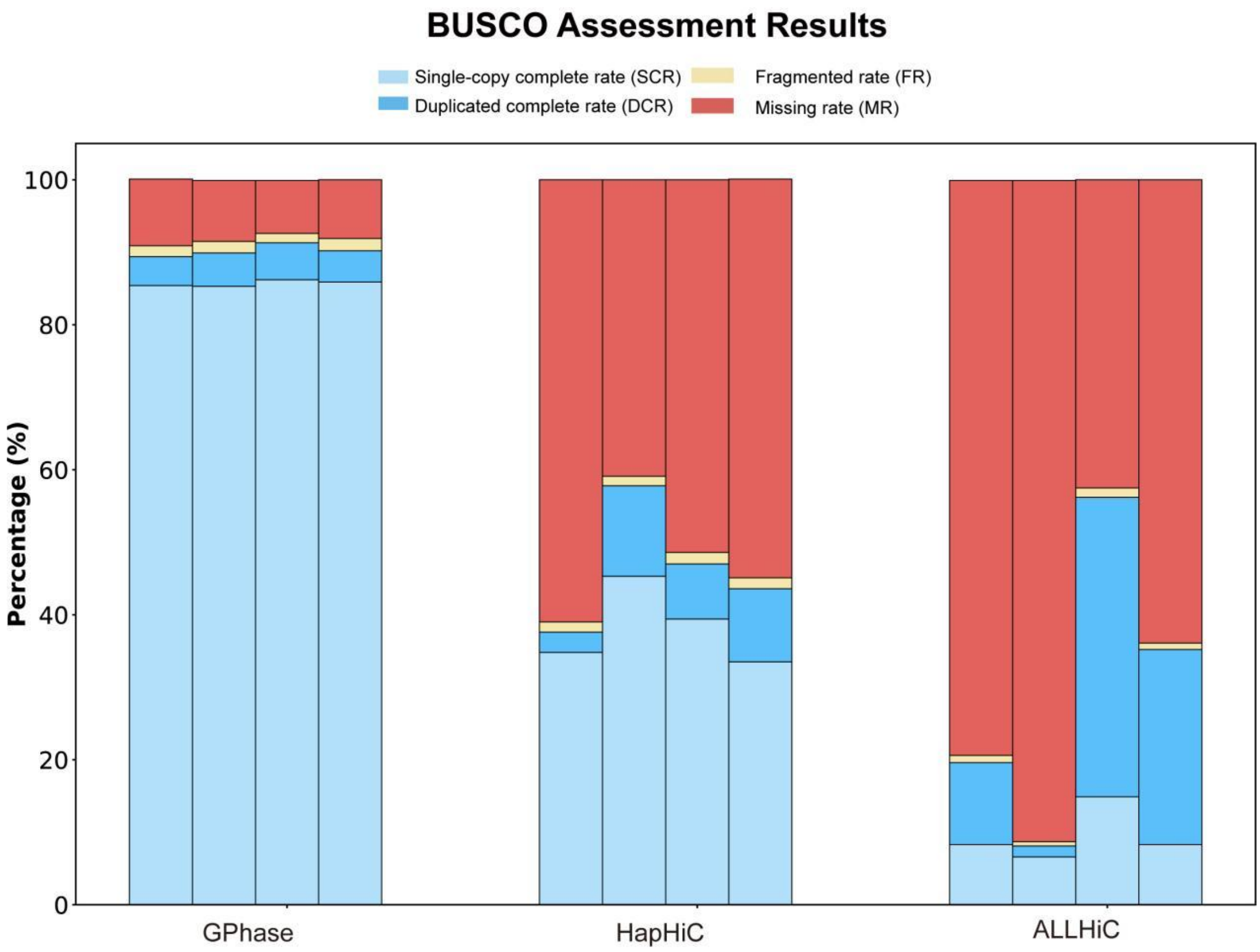

Supplementary Fig. 21: BUSCO completeness scores of the potato (*S. tuberosum* 'Otava') genome assemblies generated by the three tools. Each bar represents one haplotype.

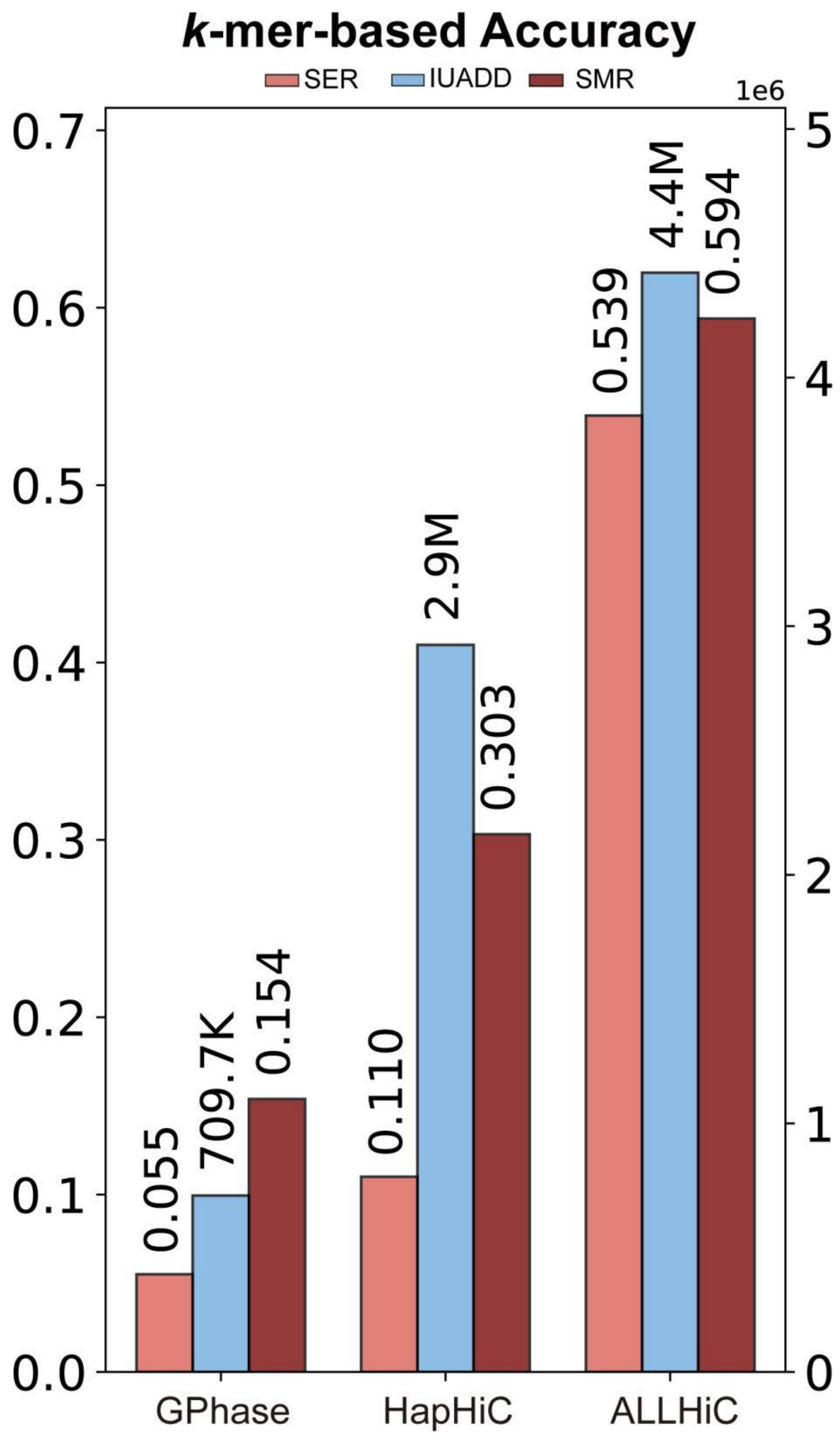

Supplementary Fig. 22: *k*-mer-based assessment scores of the potato (*S. tuberosum* 'Otava') genome assemblies generated by the three tools: Sequence Error Rate (SER), Sequence Missing Rate (SMR), and Inter-unitig Average Distance Difference (IUADD) with the lower values indicate higher accuracy.

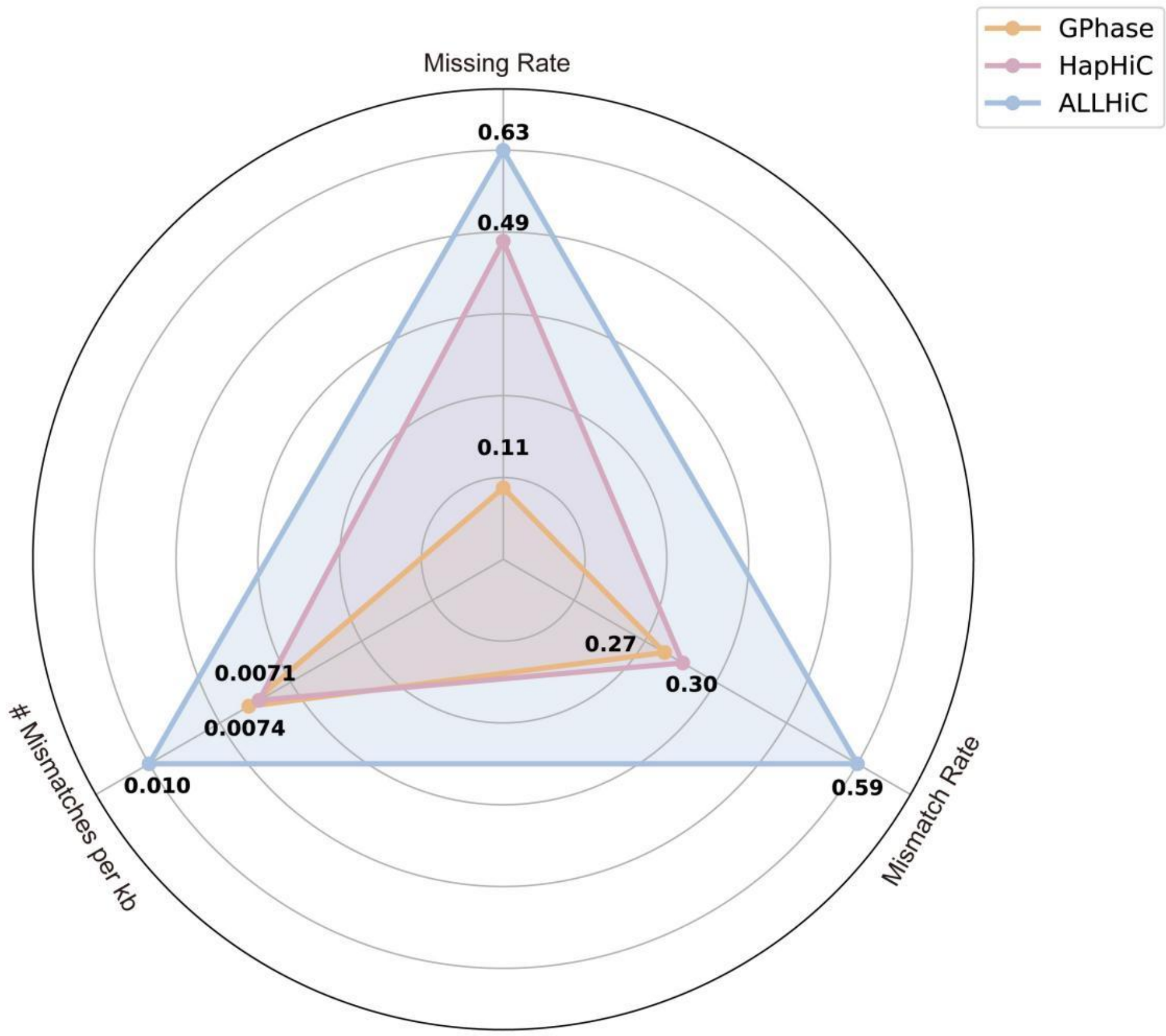

Supplementary Fig. 23: Sequence-alignment-based assessment scores of the potato (*S. tuberosum* 'Otava') genome assemblies generated by the three tools: Missing Rate, Mismatch Rate, and #Mismatches per kb with the lower values indicate higher accuracy.

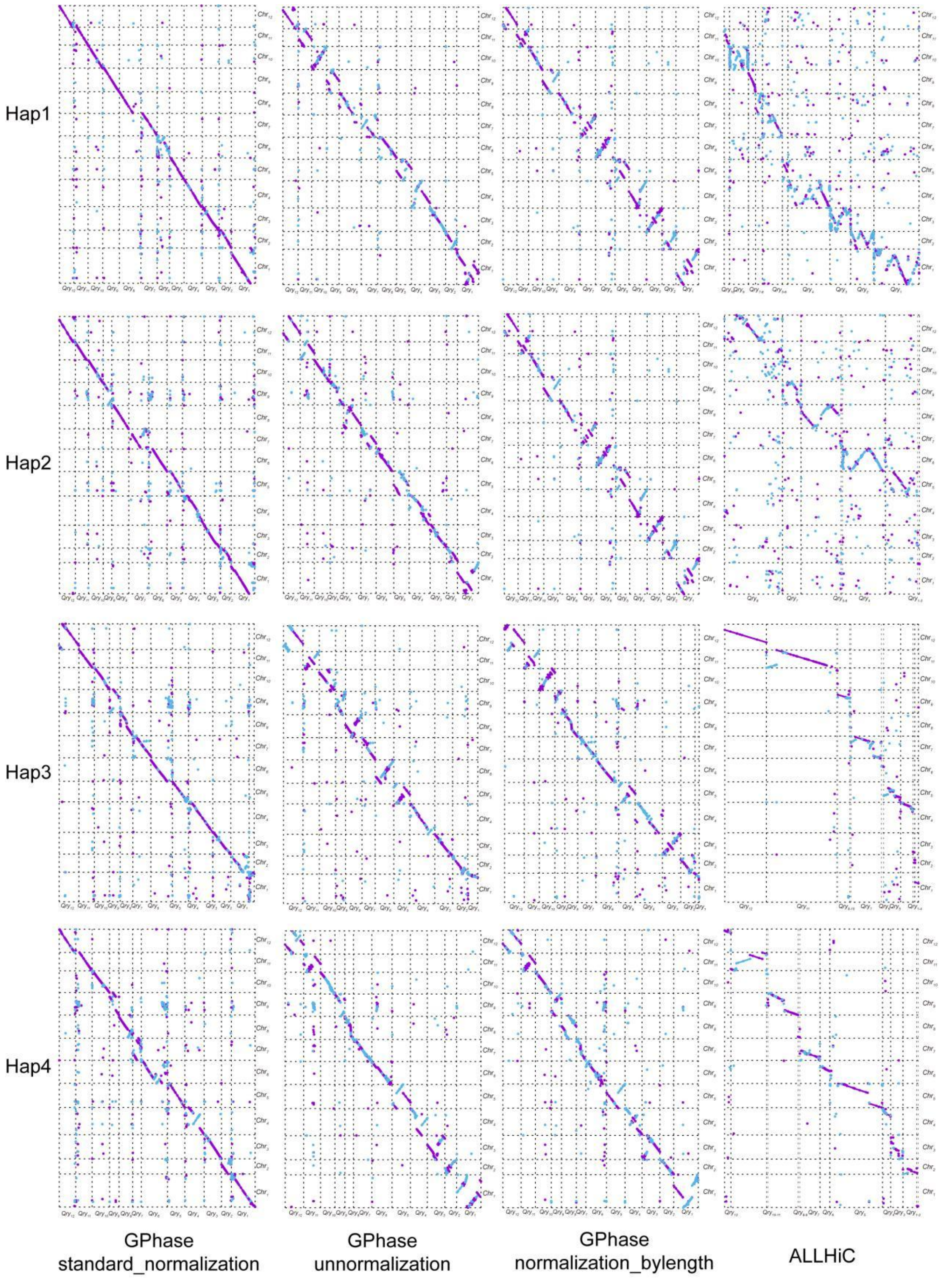

Supplementary Fig. 24: Sequence-alignment-based synteny plots of the potato (*S. tuberosum* 'Katahdin') genome assemblies generated by GPhase with different normalization methods and ALLHiC.

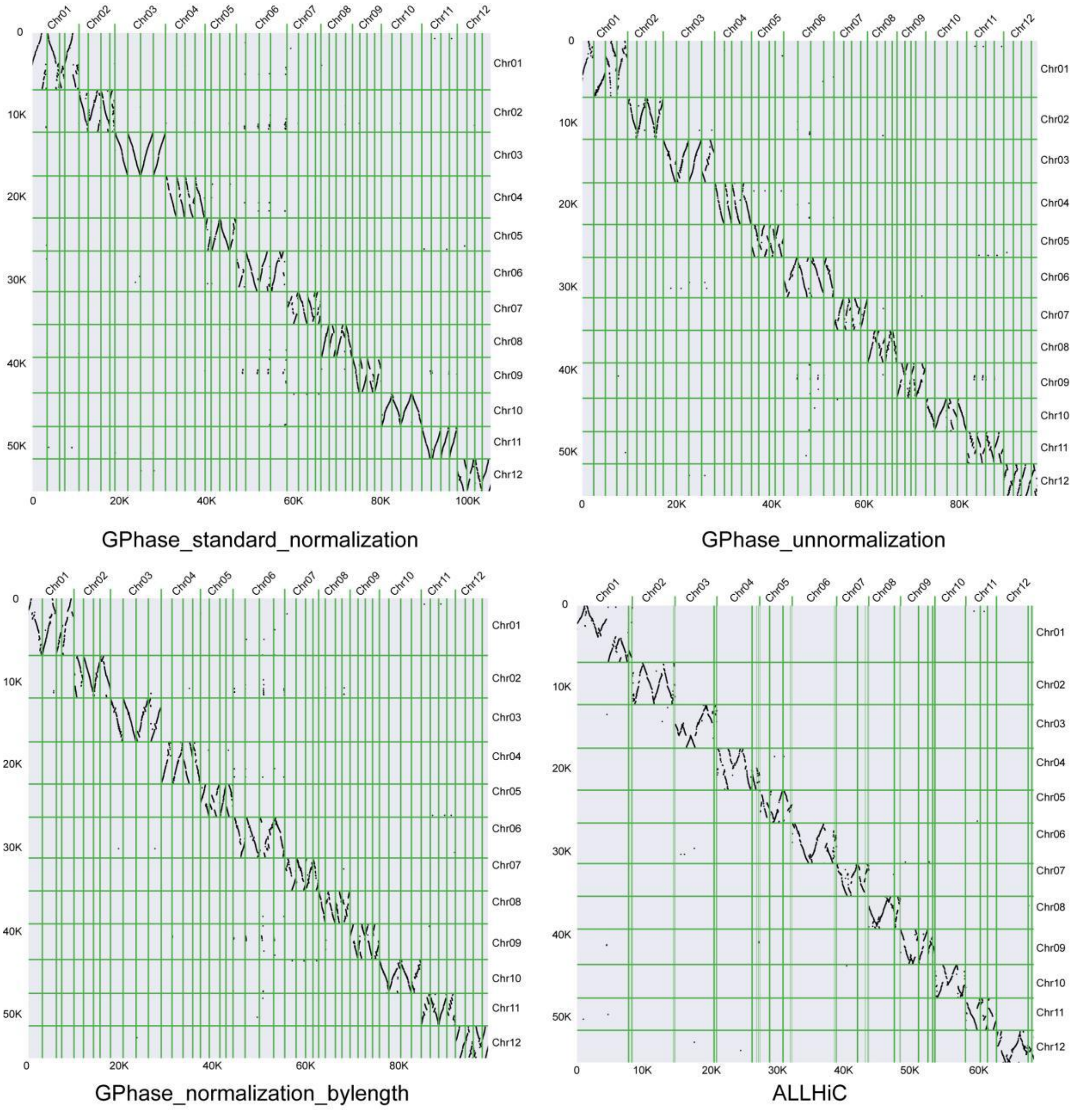

Supplementary Fig. 25: Gene-based synteny plots of the potato (*S. tuberosum* ‘Katahdin’) genome assemblies generated by GPhase with different normalization methods and ALLHiC.

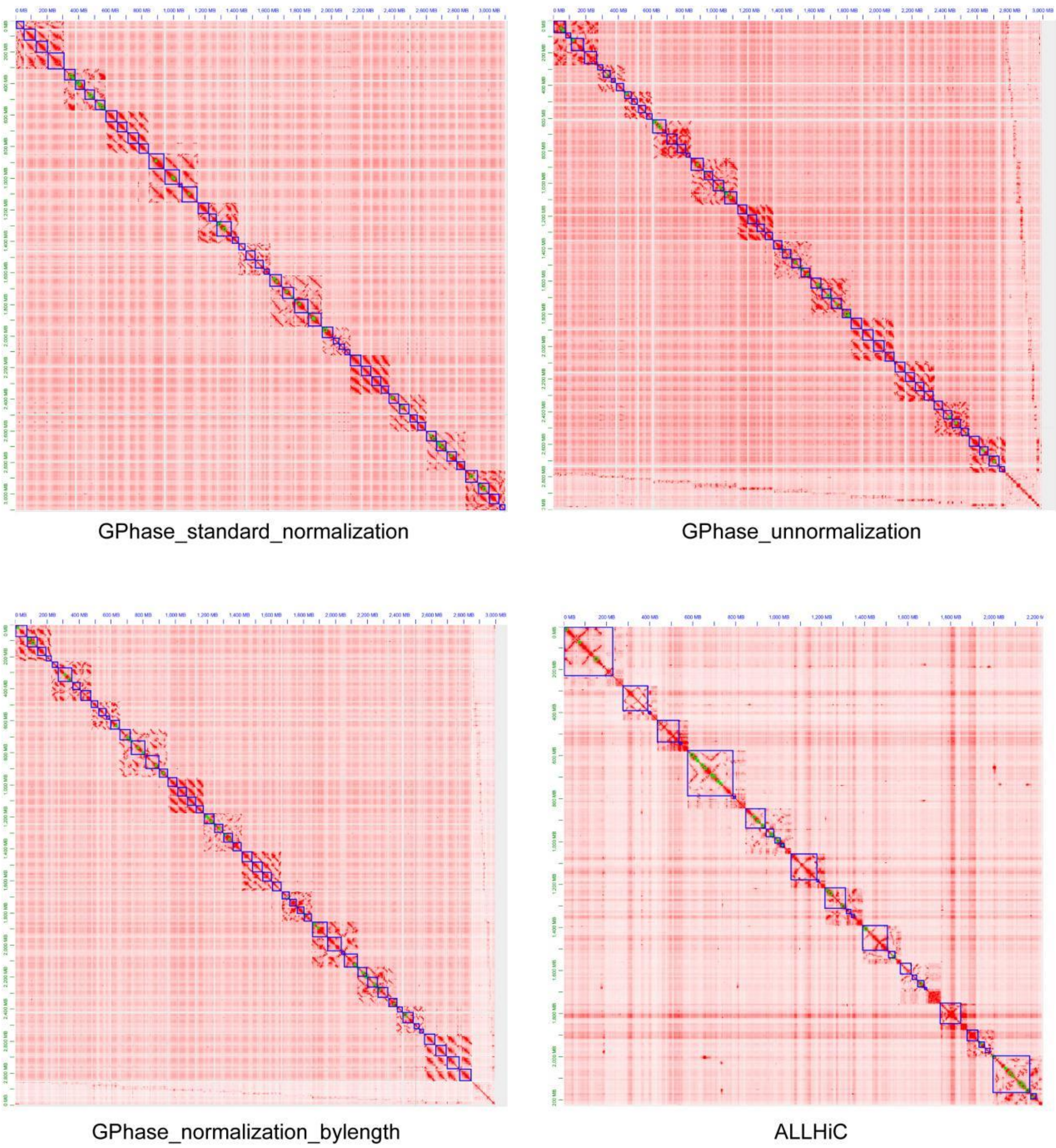

Supplementary Fig. 26: Omni-C contact maps of the potato (*S. tuberosum* 'Katahdin') genome assemblies generated by GPhase with different normalization methods and ALLHiC.

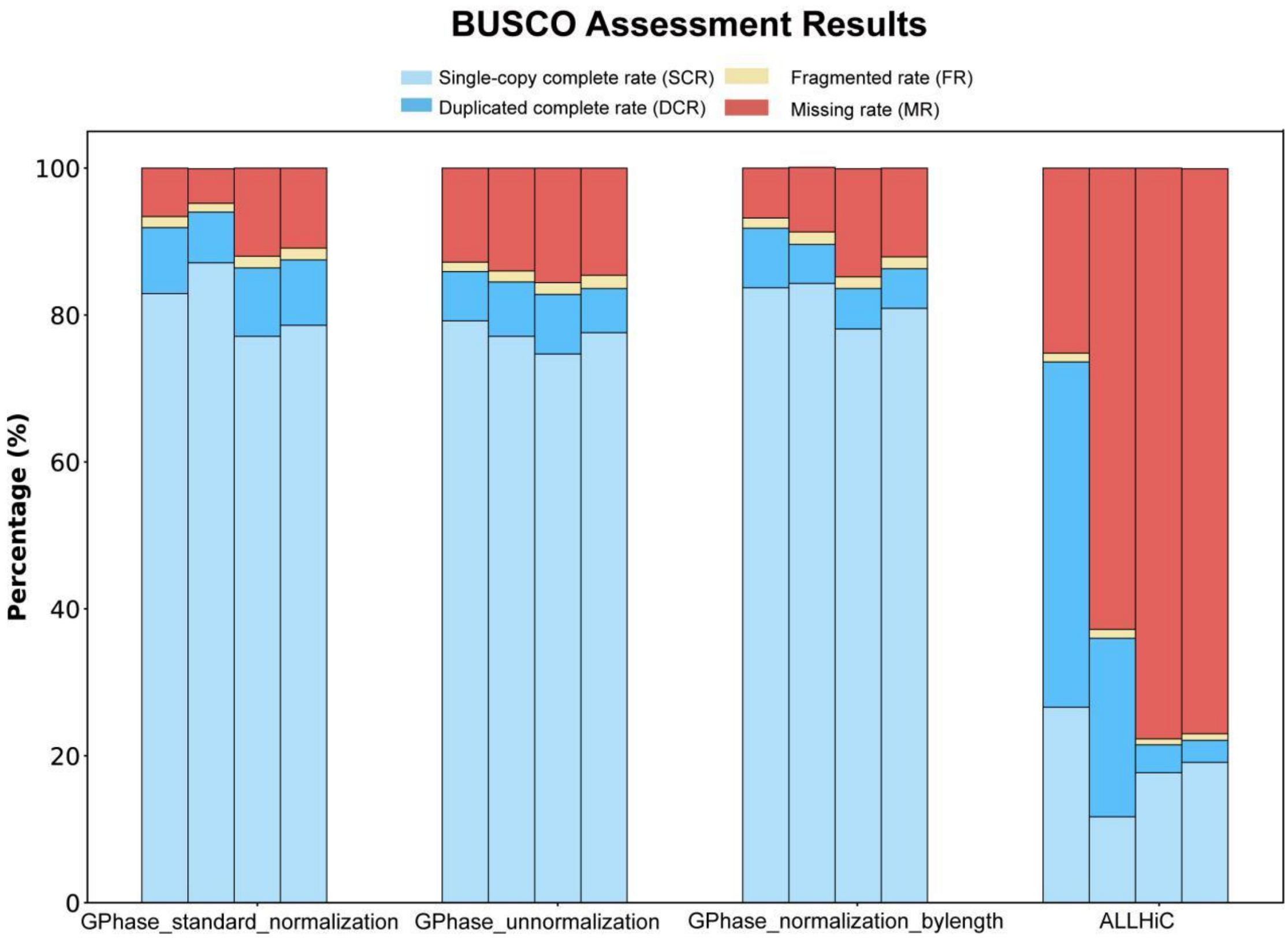

Supplementary Fig. 27: BUSCO completeness scores of the potato (*S. tuberosum* 'Katahdin') genome assemblies generated by GPhase with different normalization methods and ALLHiC. Each bar represents one haplotype.

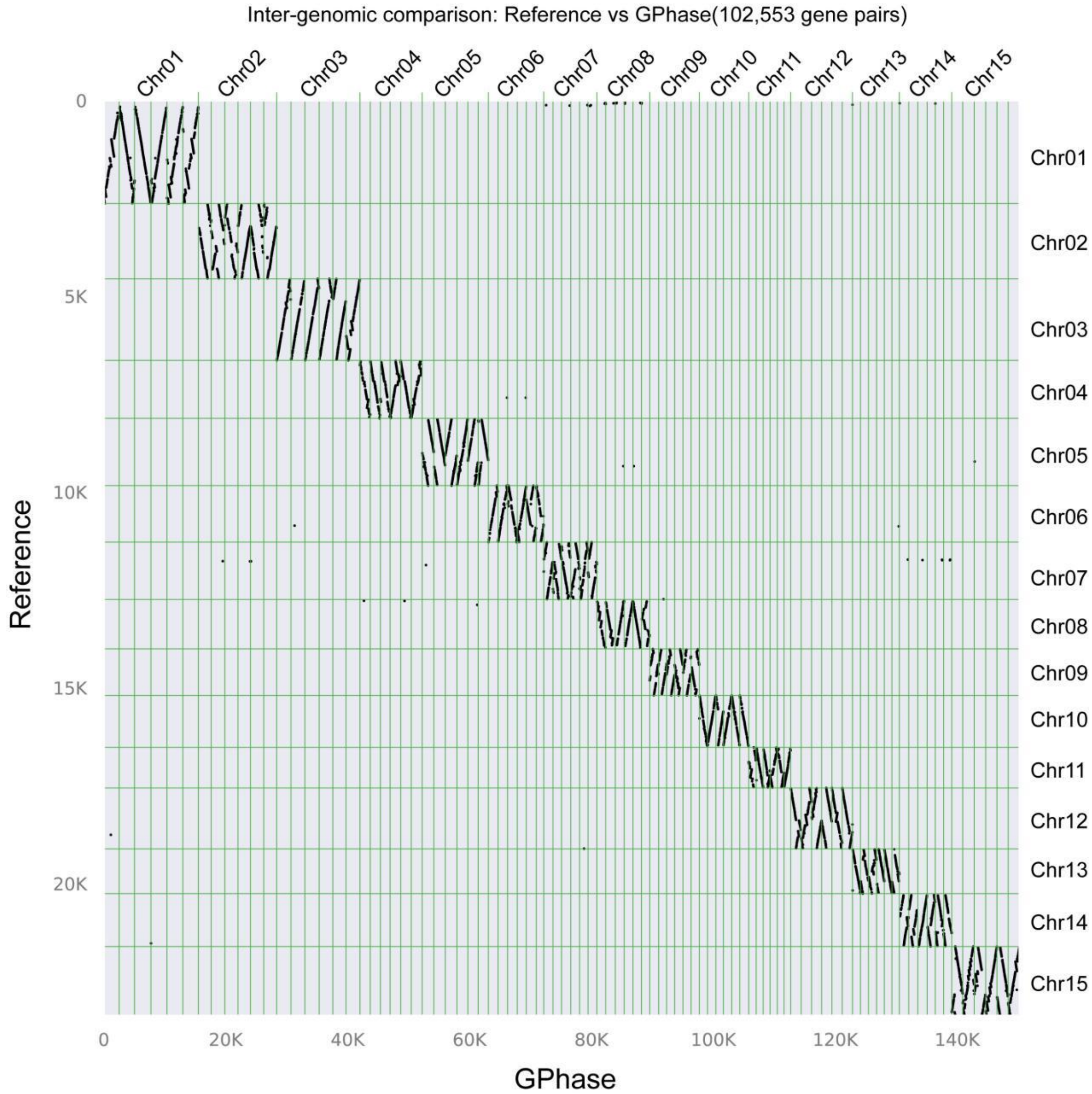

Supplementary Fig. 28: Gene-based synteny plot of the hexaploid *Diospyros kaki* 'Xiaoguotianshi' genome assembly generated by GPhase using Hi-C data.

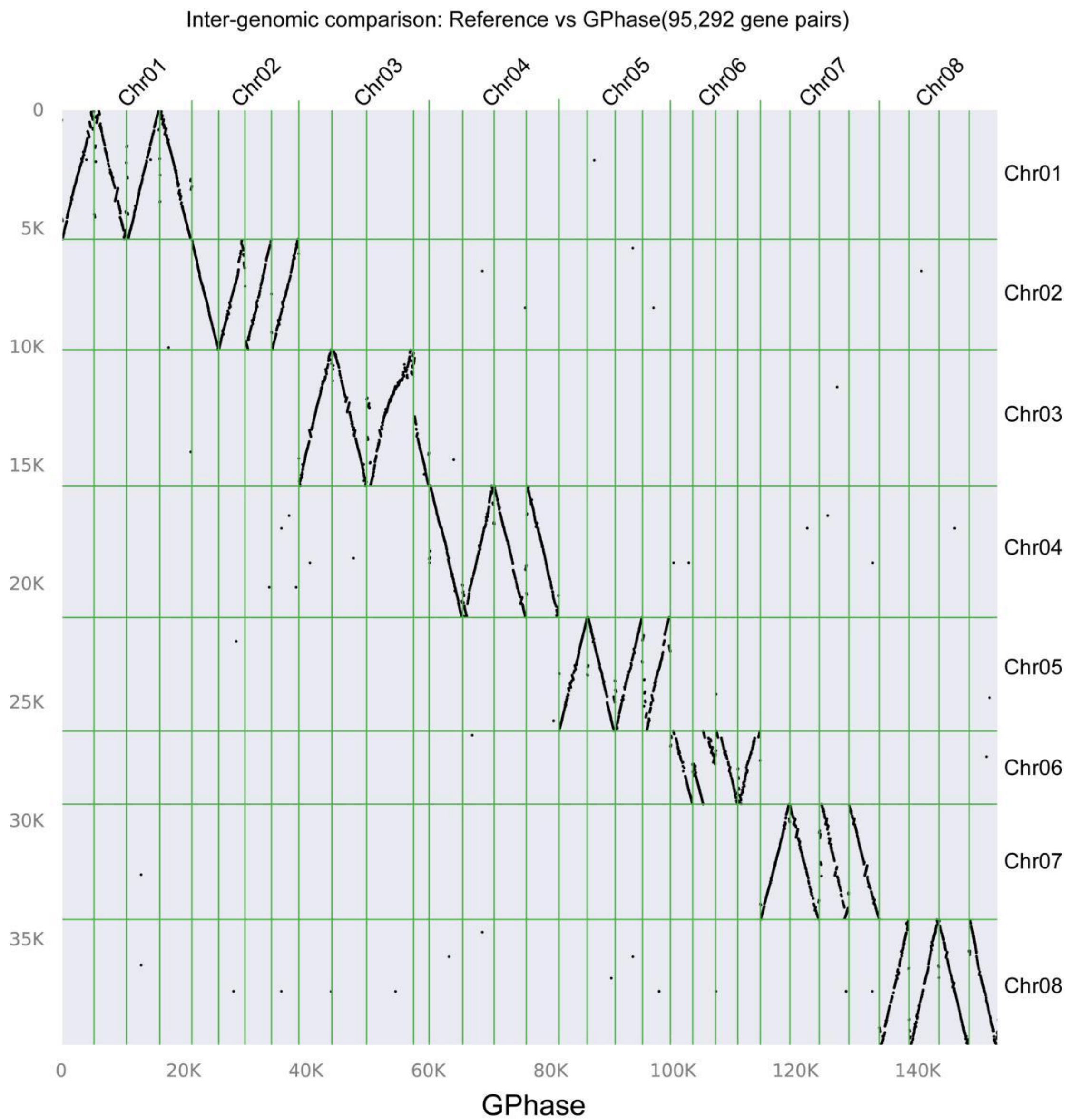

Supplementary Fig. 29: Gene-based synteny plot of a tetraploid *Medicago sativa* ‘Xinjiangdaye’ genome assembly generated by GPhase using Hi-C data.

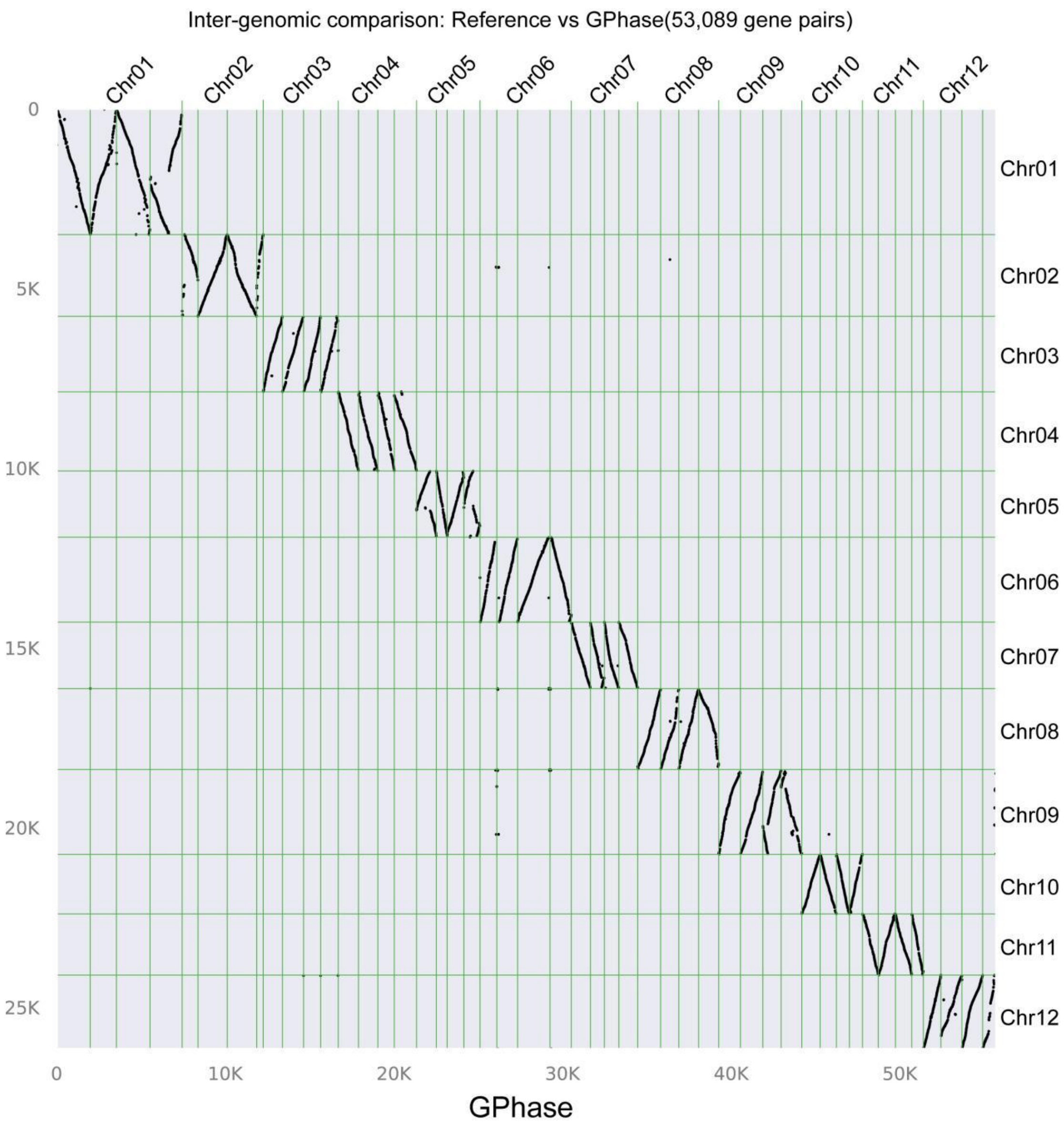

Supplementary Fig. 30: Gene-based synteny plot of the tetraploid *Ziziphus mauritiana* 'Xishuangyesheng' genome assembly generated by GPhase using Hi-C data.

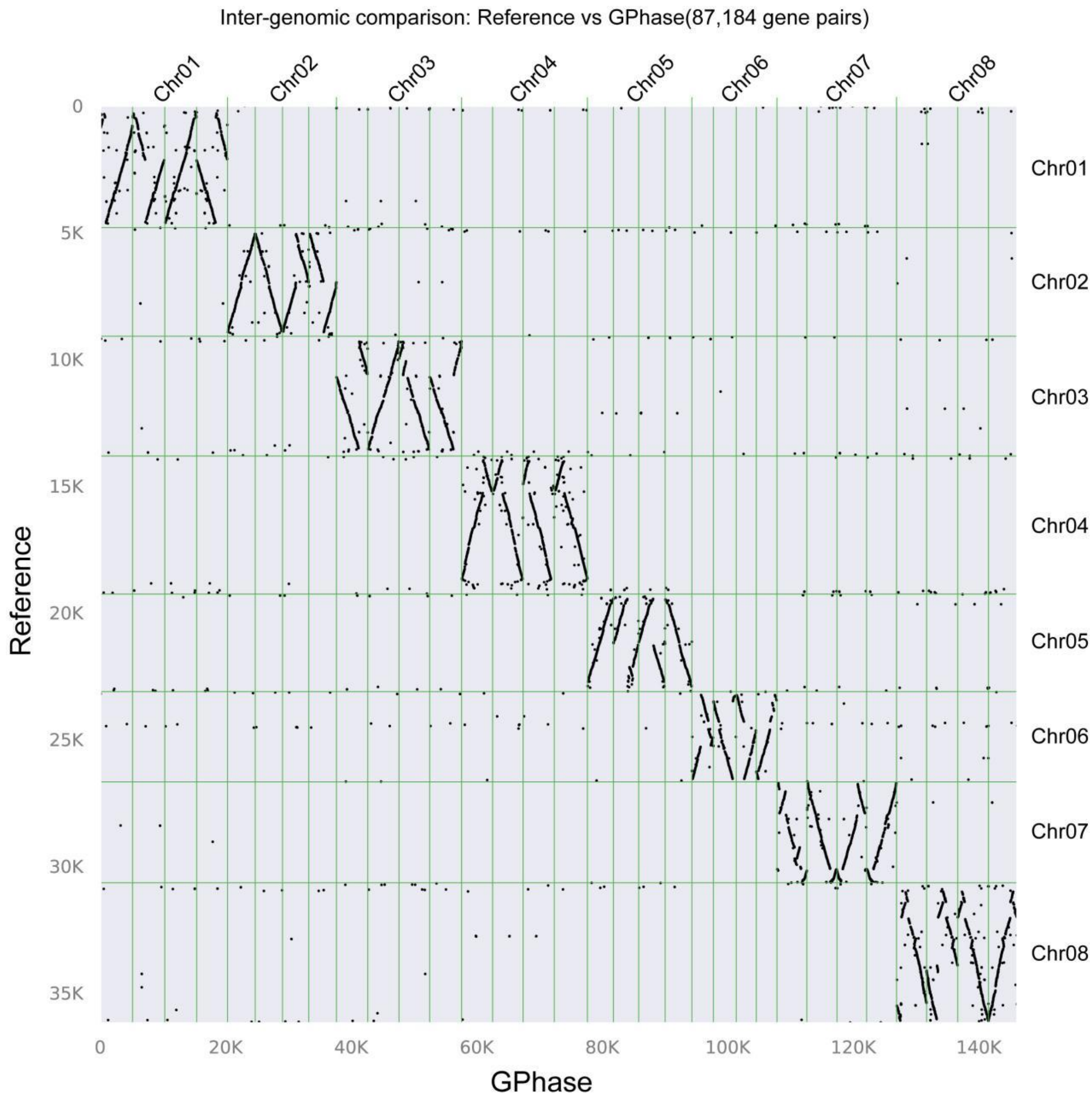

Supplementary Fig. 31: Gene-based synteny plot of the tetraploid *Medicago sativa* 'Zhongmu-4' genome assembly generated by GPhase using Pore-C data.

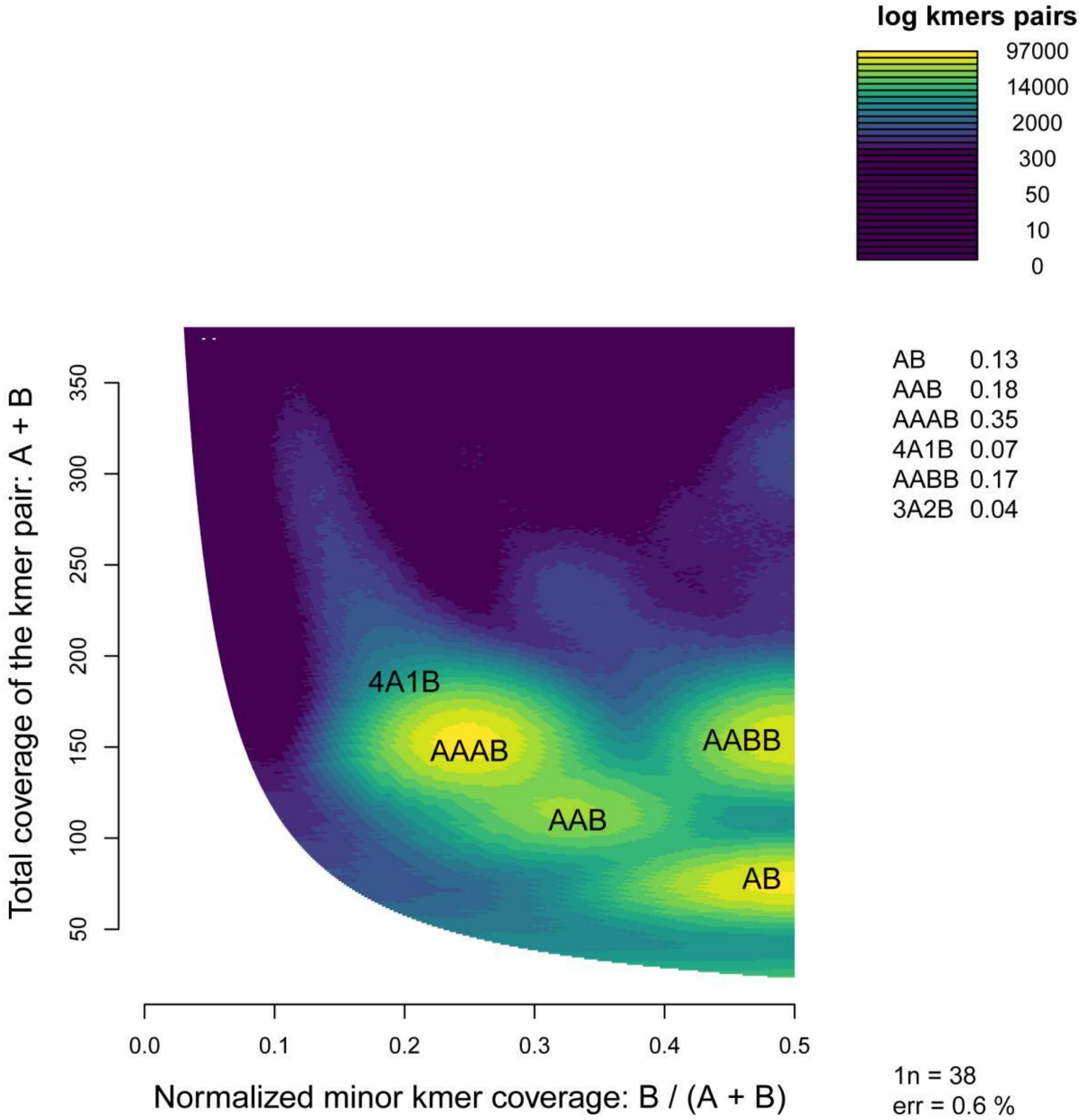

Supplementary Fig. 32: Smudgeplot-based ploidy estimation of the wax apple (*Syzygium samarangense* 'Tub') genome.

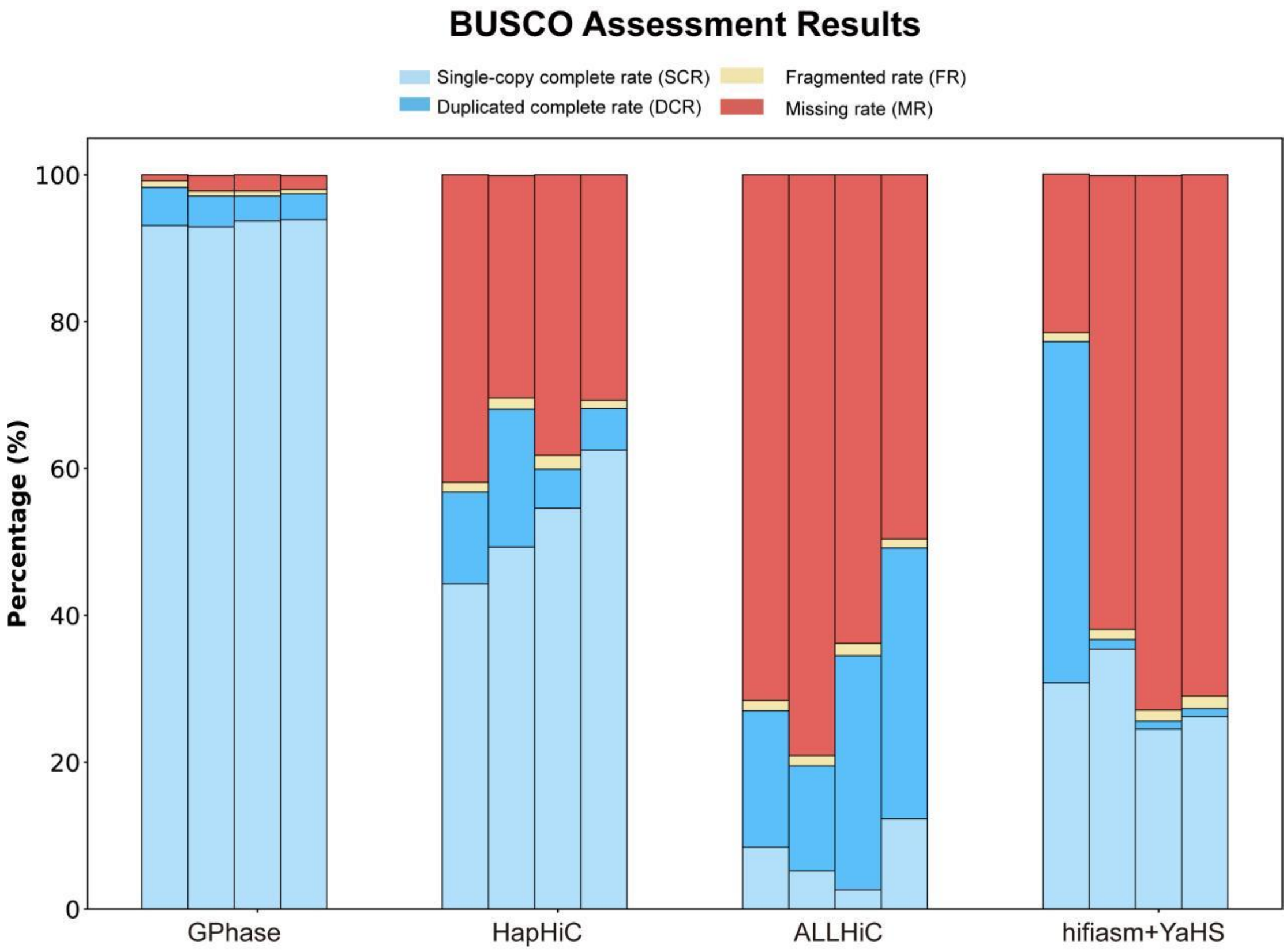

Supplementary Fig. 33: BUSCO completeness scores of the wax apple (*S. samarangense* 'Tub') genome assemblies generated by the four methods. Each bar represents one haplotype.

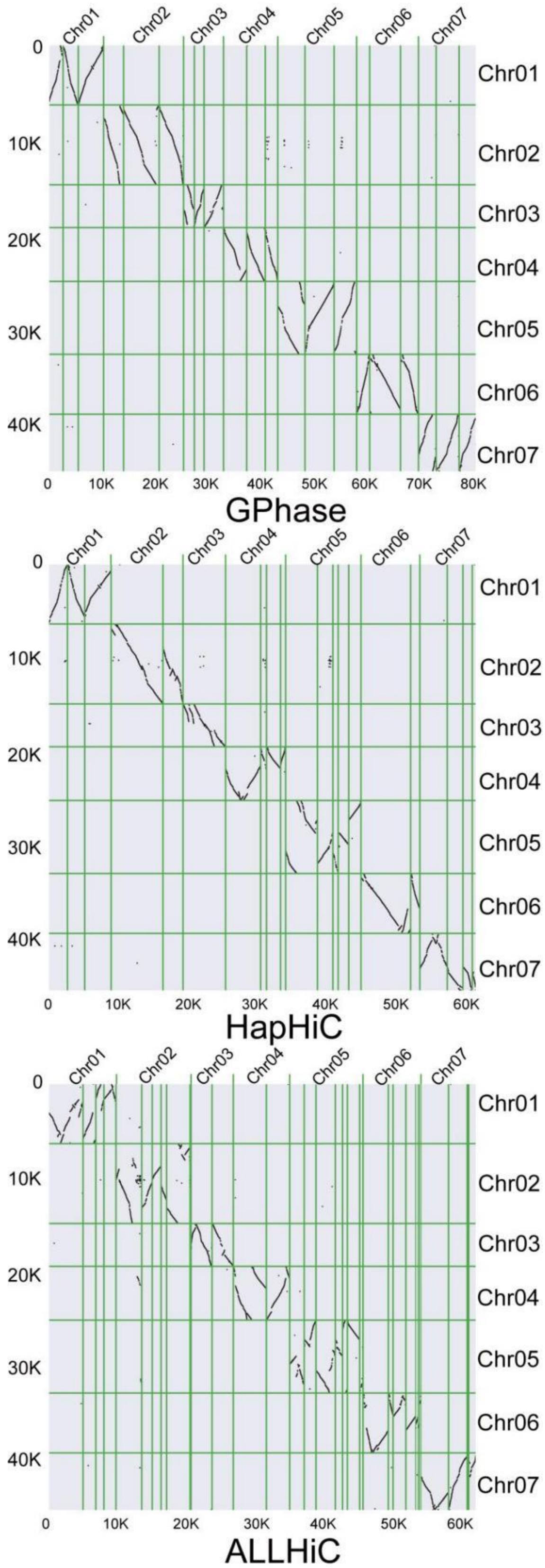

Supplementary Fig. 34: Gene-based synteny plots of chromosome-level rose (*R. chinensis* 'Jin Ou Fan Lv') genome assemblies generated by the three tools. The horizontal and vertical axes represent the assembly and the reference genome, respectively.

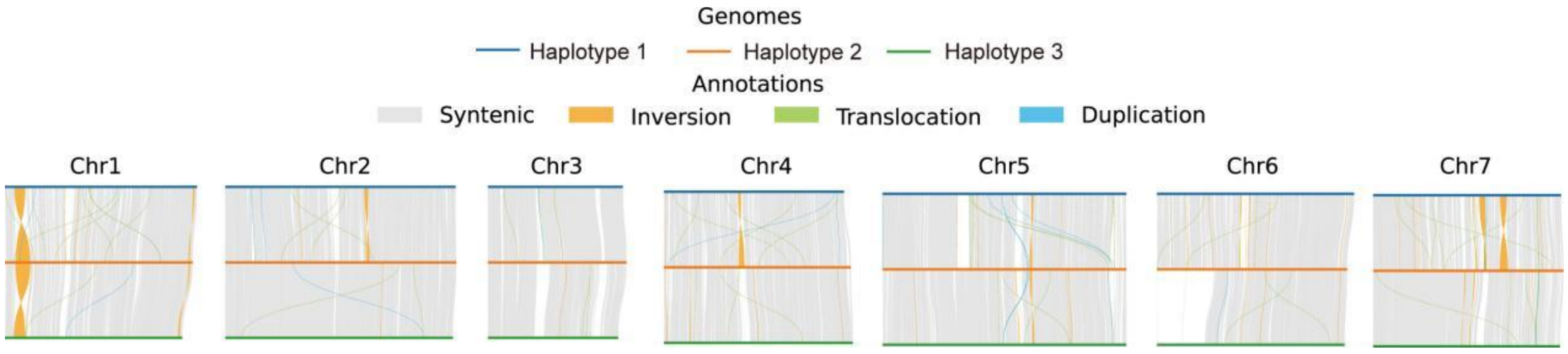

Supplementary Fig. 35: The synteny plots between the three haplotypes of each chromosome in the final rose (*R. chinensis* ‘Jin Ou Fan Lv’) genome assembly.

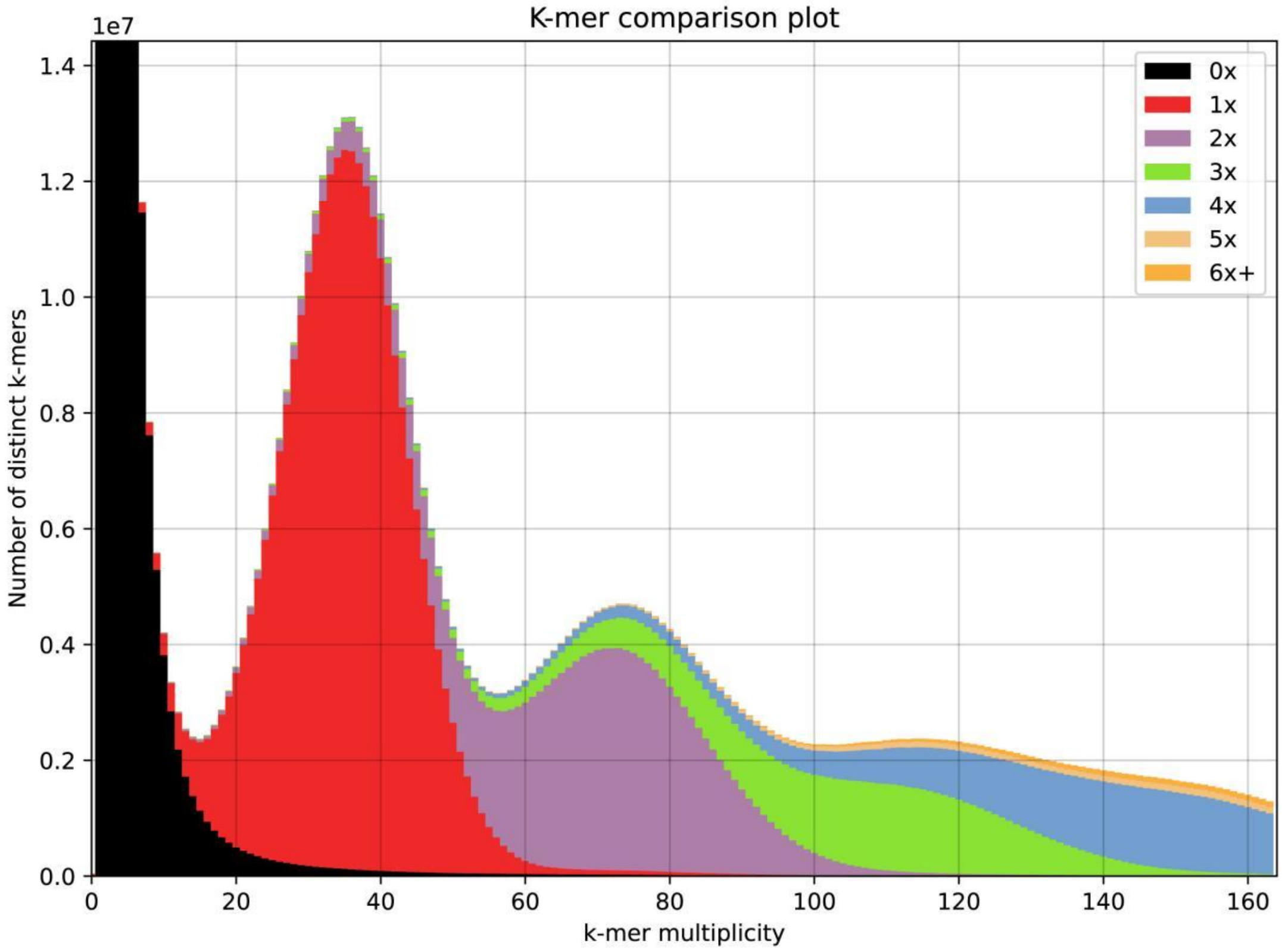

Supplementary Fig. 36: The KAT *k*-mer comparison plot for evaluating the completeness of final wax apple (*S. samarangense* 'Tub') genome assembly. No black peak corresponding to missing *k*-mers was observed, suggesting a high completeness of the assemblies.
