# Supplementary material for: Assembly-graph-assisted haplotype-resolved scaffolding of polyploid genomes
Source: Nat Plants. 2026 Oct 16;12(10):1960–75. doi: 10.1038/s41477-026-02422-5 (PMC13680272; doi:10.1038/s41477-026-02422-5)
Supplement: Supplementary file 2 [file 41477_2026_2422_MOESM2_ESM.pdf]

nature portfolio

Corresponding author(s): Weihua Pan; Zhiqiang Wu; Dongxi Li; Tingcai Cheng

Last updated by author(s): Aug. 21,2026

# Reporting Summary

Nature Portfolio wishes to improve the reproducibility of the work that we publish. This form provides structure for consistency and transparency in reporting. For further information on Nature Portfolio policies, see our Editorial Policies and the Editorial Policy Checklist.

## Statistics

For all statistical analyses, confirm that the following items are present in the figure legend, table legend, main text, or Methods section.

| n/a | Confirmed | |
|---|---|---|
| ☒ | ☐ | The exact sample size (*n*) for each experimental group/condition, given as a discrete number and unit of measurement |
| ☒ | ☐ | A statement on whether measurements were taken from distinct samples or whether the same sample was measured repeatedly |
| ☒ | ☐ | The statistical test(s) used AND whether they are one- or two-sided<br>*Only common tests should be described solely by name; describe more complex techniques in the Methods section.* |
| ☒ | ☐ | A description of all covariates tested |
| ☒ | ☐ | A description of any assumptions or corrections, such as tests of normality and adjustment for multiple comparisons |
| ☒ | ☐ | A full description of the statistical parameters including central tendency (e.g. means) or other basic estimates (e.g. regression coefficient) AND variation (e.g. standard deviation) or associated estimates of uncertainty (e.g. confidence intervals) |
| ☒ | ☐ | For null hypothesis testing, the test statistic (e.g. *F*, *t*, *r*) with confidence intervals, effect sizes, degrees of freedom and *P* value noted<br>*Give P values as exact values whenever suitable.* |
| ☒ | ☐ | For Bayesian analysis, information on the choice of priors and Markov chain Monte Carlo settings |
| ☒ | ☐ | For hierarchical and complex designs, identification of the appropriate level for tests and full reporting of outcomes |
| ☒ | ☐ | Estimates of effect sizes (e.g. Cohen's *d*, Pearson's *r*), indicating how they were calculated |

*Our web collection on statistics for biologists contains articles on many of the points above.*

## Software and code

Policy information about availability of computer code

| | |
|---|---|
| Data collection | GNU Wget (version 1.14)<br>SRA Toolkit (version 2.10.8) |

Data analysis

GPhase and all custom scripts are available on GitHub at https://github.com/panlab-bioinfo/GPhase.
For mapping and filtering of 3C eads: Chromap(vesion v0.2.7-r494), BWA (version v0.7.18-r1243-dirty), PPL-Toolbox (version v0.1.1), and juicer pipeline (https://github.com/aidenlab/juicer, vesion: commit 827fe44)
For ploidy estimation:Smudgeplot (version v0.5.3) and genomescope 2.0 (version v1.0.0)
For sequencing data simulation:Badread (vesion v0.4.1), SeqKit (version v2.10.1) and sim3C (vesion v0.7.1)
Other scaffolding tools used in benchmark or assembly of a reference dataset: ALLHiC (version 0.9.13), HapHiC (version v1.0.6), YaHS (version v1.2.2), Ragtag(version v2.1.0), 3D-DNA (version v201008) and C-Phasing (version 0.2.5.r291).
For scaffolding result visualization: HapHiC_plot scripts (https://github.com/zengxiaofei/HapHiC/blob/main/scripts/HapHiC_plot.py, GitHub commit 0f05366), IGV(version 2.19.7), Bandage(version 0.8.1), and juicebox (version 1.11.08).
For genome assembly: hifiasm (version v0.19.5-r592 for 'YF47' and 'FL478',version v0.24.0-r703 for others),Flye (version v2.9.3-b1797), Peregrine(version v1.58.0), Verkko (version v2.2.1)
For genome alignment, structural variation identification and alignment visualization: minimap2 (version 2.26-r1175),MUMmer (version v4.0.1), JCVI (version v1.4.16), and SyRI (version 1.7.1).
For genome assembly quality assessment: haplotype_kmers.py (https://github.com/zengxiaofei/HapHiC/blob/main/simulation/haplotype_kmers.py, GitHub commit 431b7b6)， BUSCO (version v5.8.3),PolySwitch(https://github.com/eden00Chen/polyswitch, GitHub commit 6cedfeb),KAT (version v2.4.2),CRAQ (version v1.10), Merqury (version v1.3), and LTR_finder (version v1.0.7), and LTR_retriever (version v3.0.2).

For manuscripts utilizing custom algorithms or software that are central to the research but not yet described in published literature, software must be made available to editors and reviewers. We strongly encourage code deposition in a community repository (e.g. GitHub). See the Nature Portfolio guidelines for submitting code & software for further information.

## Data

Policy information about availability of data

All manuscripts must include a data availability statement. This statement should provide the following information, where applicable:

- Accession codes, unique identifiers, or web links for publicly available datasets
- A description of any restrictions on data availability
- For clinical datasets or third party data, please ensure that the statement adheres to our policy

All newly generated sequencing data used in this study, together with the final chromosome-level assemblies of Rosa chinensis 'Jin Ou Fan Lv' and Syzygium samarangense 'Tub', have been deposited in the National Genomics Data Center (NGDC, https://ngdc.cncb.ac.cn) under BioProject ID PRJCA045971. For R. chinensis 'Jin Ou Fan Lv', both PacBio HiFi reads (CRR2125246) and Hi-C reads (CRR2125247) were generated in this study. Manual curation yielded the final chromosome-level assembly (GWHGRYV00000000), and the reference sequence is available at https://iris.angers.inra.fr/obh. For S. samarangense 'Tub', ONT ultra-long reads (CRR2125340) were generated in this study, whereas the PacBio HiFi reads (SRR23279229 and SRR23279230) and Hi-C reads (CRR568487) together with Illumina reads (CRR568488, CRR568489, and CRR568490) were obtained from published datasets. Manual curation yielded the final chromosome-level assembly, version 2 (GWHGQMQ00000000); the previously published reference genome, version 1, is recorded in the NGDC Genome Warehouse under accession GWHDVFZ00000000 (https://ngdc.cncb.ac.cn/gwh). The newly generated PacBio HiFi, ONT ultra-long, and Hi-C data for the haploid rice benchmark datasets based on Oryza sativa 'YF47' (CRR2127807, CRR2127808, and CRR2127809) and 'FL478' (CRR2127804, CRR2127805, and CRR2127806) were used to construct simulated benchmark datasets. The corresponding ground-truth assemblies are available at https://huggingface.co/datasets/YANchunX/Assemblies_generated_for_the_GPhase_benchmark_datasets/blob/main/rice_ground_truth/YF47.ref.fasta.gz and https://huggingface.co/datasets/YANchunX/Assemblies_generated_for_the_GPhase_benchmark_datasets/blob/main/rice_ground_truth/FL478.ref.fasta.gz, respectively. The remaining benchmark datasets were based on published sequencing data. These include the polyploid potato datasets Solanum tuberosum 'Otava' and 'Katahdin', the polyploid persimmon dataset Diospyros kaki Thunb 'Xiaoguotianshi', the polyploid alfalfa datasets Medicago sativa 'Xinjiangdaye' and 'Zhongmu-4', and the additional published datasets Rosa hybrida 'Samantha' and Ziziphus mauritiana 'Xishuangyesheng'. For R. hybrida 'Samantha', PacBio HiFi reads (SRR28905077), ONT ultra-long reads (SRR28905048), Pore-C reads (SRR28905076), Hi-C reads (SRR28905065), and MGI paired-end reads (SRR28905054) were obtained from NCBI; the reference assembly and annotation are available at https://iris.angers.inra.fr/obh, and the ground-truth assembly is available at https://doi.org/10.6084/m9.figshare.22774097. For S. tuberosum 'Otava', PacBio HiFi reads (SRR15206231) and Hi-C reads (SRR15198317, SRR15248701, and SRR15248702) were obtained from NCBI; the reference sequence is available at http://spuddb.uga.edu/c88_potato_download.shtml, and the ground-truth assembly is represented by JAIVGA000000000, JAIVGB000000000, JAIVGC000000000, and JAIVGD000000000, which can be retrieved from NCBI. For S. tuberosum 'Katahdin', PacBio HiFi reads (SRR32121363) and Omni-C reads (SRR32121374) were obtained from NCBI; the reference sequence is available at http://spuddb.uga.edu/c88_potato_download.shtml, and the ground-truth assembly is available at https://doi.org/10.5281/zenodo.14053896. The three additional rice genomes used to construct simulated datasets—O. sativa 'BD8', 'Nipponbare', and '9311'—were also based on published data. For O. sativa 'BD8', PacBio HiFi reads (CRR1720551), ONT reads (CRR1720552), and Hi-C reads (CRR1720553) were obtained from NGDC, and the ground-truth genome is GWHFWBB00000000. For O. sativa 'Nipponbare', PacBio HiFi reads (SRR25241090), ONT ultra-long reads (SRR25241091), and Hi-C reads (SRR25241089) were obtained from NCBI, and the ground-truth/reference genome is available at http://www.ricesuperpir.com/web/nip. For O. sativa '9311', PacBio HiFi reads (CRR1644118), ONT ultra-long reads (CRR1644119), and Hi-C reads (CRR1644120) were obtained from NGDC, and the ground-truth genome is GWHFLAP00000000. For D. kaki Thunb 'Xiaoguotianshi', PacBio HiFi reads (SRR18500470, SRR18500471, SRR18500472, SRR18500473, SRR18500474, SRR18500475, SRR18500476, and SRR18500477) and Hi-C reads (SRR18500481, SRR18500482, SRR18500483, SRR18500484, SRR18500485, SRR18500486, SRR18500487, and SRR18500488) were obtained from NCBI, and the ground-truth assembly is available at https://doi.org/10.6084/m9.figshare.19447256. For Z. mauritiana 'Xishuangyesheng', PacBio HiFi reads (SRR25180207 and SRR24951734) and Hi-C reads (SRR24951729) were obtained from NCBI, and the ground-truth assembly is available at https://doi.org/10.6084/m9.figshare.23530068. For M. sativa 'Xinjiangdaye', PacBio HiFi reads (SRR11285798) and Hi-C reads (SRR9026577) were obtained from NCBI, and the ground-truth assembly source is available at https://doi.org/10.6084/m9.figshare.12327602. For M. sativa 'Zhongmu-4', PacBio HiFi reads (CRR2374621), ONT ultra-long reads (CRR2374622), and Pore-C reads (CRR2374624) were obtained from NGDC; the reference assembly source is available at https://doi.org/10.6084/m9.figshare.12327602, and the ground-truth assembly and annotation are available at https://doi.org/10.6084/m9.figshare.30768716. SRR accessions can be searched in NCBI (https://www.ncbi.nlm.nih.gov/), whereas CRR accessions can be searched in NGDC (https://ngdc.cncb.ac.cn).

## Research involving human participants, their data, or biological material

Policy information about studies with human participants or human data. See also policy information about sex, gender (identity/presentation), and sexual orientation and race, ethnicity and racism.

| | |
|---|---|
| Reporting on sex and gender | No human participants were recruited for this study. |
| Reporting on race, ethnicity, or other socially relevant groupings | No human participants were recruited for this study. |
| Population characteristics | No human participants were recruited for this study. |
| Recruitment | No human participants were recruited for this study. |
| Ethics oversight | No human participants were recruited for this study. |

Note that full information on the approval of the study protocol must also be provided in the manuscript.

# Field-specific reporting

Please select the one below that is the best fit for your research. If you are not sure, read the appropriate sections before making your selection.

☒ Life sciences ☐ Behavioural & social sciences ☐ Ecological, evolutionary & environmental sciences

For a reference copy of the document with all sections, see nature.com/documents/nr-reporting-summary-flat.pdf

# Life sciences study design

All studies must disclose on these points even when the disclosure is negative.

| | |
|---|---|
| Sample size | The paper describes a software tool to process certain biological data and study design is not applicable here. |
| Data exclusions | No data was excluded from the analysis. |
| Replication | Experimental replication was not attempted here. |
| Randomization | Not applicable since no group allocation was performed. |
| Blinding | Not applicable since no group allocation was performed. |

# Reporting for specific materials, systems and methods

We require information from authors about some types of materials, experimental systems and methods used in many studies. Here, indicate whether each material, system or method listed is relevant to your study. If you are not sure if a list item applies to your research, read the appropriate section before selecting a response.

### Materials & experimental systems

| n/a | Involved in the study | |
|---|---|---|
| ☒ | ☐ | Antibodies |
| ☒ | ☐ | Eukaryotic cell lines |
| ☒ | ☐ | Palaeontology and archaeology |
| ☒ | ☐ | Animals and other organisms |
| ☒ | ☐ | Clinical data |
| ☒ | ☐ | Dual use research of concern |
| ☐ | ☒ | Plants |

### Methods

| n/a | Involved in the study | |
|---|---|---|
| ☒ | ☐ | ChIP-seq |
| ☒ | ☐ | Flow cytometry |
| ☒ | ☐ | MRI-based neuroimaging |

## Plants

Seed stocks

'YF47' and 'FL478' were collected from rice seedlings grown in the experimental field of the Agricultural Genomics Institute at Shenzhen, Chinese Academy of Agricultural Sciences (Guangdong Province, China).Syzygium samarangense 'Tub' were collected from an individual tree grown in the experimental field of the Fujian Academy of Agricultural Sciences (Fujian Province, China).Rosa chinensis 'Jin Ou Fan Lv' were collected from plants grown in the Flower Garden of Huazhong Agricultural University (Hubei Province, China).

Novel plant genotypes

Not applicable since no novel plant genotypes were generated in this study.

Authentication

N/A
