# Supplementary material for: Assembly-graph-assisted haplotype-resolved scaffolding of polyploid genomes
Source: Nat Plants. 2026 Oct 16;12(10):1960–75. doi: 10.1038/s41477-026-02422-5 (PMC13680272; doi:10.1038/s41477-026-02422-5)
Supplement: Supplementary file 3 [file 41477_2026_2422_MOESM3_ESM.pdf]

nature portfolio

Peer Review File

GPhase: Assembly Graph-Assisted Haplotype-Resolved Scaffolding of Polyploid Genome

Corresponding Author: Professor Weihua Pan

Version 0:

Reviewer comments:

Reviewer #1

(Comments for the Author)
I read the article with great interest and would like to congratulate the authors on developing a very interesting tool and providing a thorough benchmark of its performance. The manuscript describes a bioinformatic tool dedicated to the scaffolding of polyploid genomes, a task that remains challenging even with the availability of long-read and long-range technologies. Polyploid genomes can result from different evolutionary scenarios, and their histories may have shaped genomic architectures that include both regions with little or no homozygosity between haplotypes and, conversely, regions that are sufficiently divergent to be clearly distinguished. This mosaic pattern of genomic regions makes the generalization of polyploid genome assembly particularly difficult. Although this issue is not specific to plant genomes (even if polyploidy is more common in this phylum), I was somewhat surprised that the authors did not submit their work to a more generalist journal within the Nature portfolio.

Although the method and the tool are promising and make me enthusiastic, I should note that the manuscript could be improved before publication.
The manuscript is rather long and contains a fair amount of redundancy, particularly in the benchmarking section. The evaluation of the results produced by the three tools (GPhase, ALLHiC, and HapHiC) is repeated for each dataset, which feels somewhat redundant. I suggest that the authors consider moving the detailed figures (dot plots, Hi-C maps, BUSCO scores, K-mer analyses) to the Supplementary Data and include instead a single figure summarizing the results across the five datasets. While all the reported metrics are valuable, dot plots and Hi-C maps have limited utility for comparing methods, as they rely mainly on visual inspection. A comparison based on the proportion of collapsed regions and haplotype mixing would likely be more informative and impactful.

In my opinion, the stepwise validation of GPhase is not very informative and could be removed or moved to the Supplementary Information. Indeed, obtaining reliable ground-truth information is not straightforward, particularly as mentioned by the authors with the misclassification issue in RagTag (L316). Another example is the statement that the graph partitioning results produced by GPhase “closely match” the ground truth. The term closely is vague and not quantitative, and, when examining Figure 3A, readers will likely find it difficult to perceive clear differences between the GPhase graph partitioning and the ground truth.

The comparison between the two genome assemblies of the autotetraploid wax apple is, in my opinion, out of scope. For example, the differences observed for Chromosomes 5 and 6–11 are mainly due to the fact that the first version was not properly manually curated. The authors should therefore move this comparison to the Supplementary Data and focus the main manuscript on the comparison of polyploid scaffolding methods.

Likewise, I would expect some evaluation of how a de novo assembly generated with Hifiasm followed by YaHS scaffolding performs, as this would be of interest to readers. Since this is a common approach, users need to understand the impact of using a non-dedicated scaffolding strategy. More importantly, Hifiasm is theoretically able to handle polyploid genomes when using Hi-C data, one can provide the Hi-C data as input and specify the expected ploidy using the n_hap parameter. It would therefore be useful to know how this method performs in comparison to GPhase. Generally, to estimate ploidy, researchers rely on the Smudgeplot tool. I think it would again be useful for readers to see, through these concrete examples, how accurate Smudgeplot’s predictions are.

In Figures 2E and S5, there are many white bins. The authors should clarify whether these correspond to regions resulting from haplotype mixing (which could indicate a limitation of the method) or to regions that cannot be distinguished between haplotypes. I assume both cases are represented, but the authors should clearly specify what these white bins represent and, ideally, use additional colors in the figures to distinguish between the two situations.

When trying to download the assemblies generated for the benchmark datasets, I was unable to find the complete set of assemblies, as only the final ones for wax apple and China rose were available. Please, provide readers with access to all assemblies generated in the study, along with a supplementary table listing standard assembly metrics and the accession numbers for each.
More importantly, I tested the program on my own dataset. First, the installation requires access to a repository that provides very little information and is entirely written in Chinese, which does not inspire confidence for end users. I recommend that the authors distribute a containerized version of the tool (for example, using Apptainer) to simplify installation, since not all users have unrestricted access to Conda. The workflow also includes a pipeline dedicated to the initial step (gphase popcnv …), but the script does not seem to account for this workflow; the authors should address this issue.
Finally, I launched the tool on a pentaploid genome, and only the first step (popCNV) completed successfully. The GPhase workflow crashed, apparently due to a clustering error for chromosomes 1 and 17, resulting in missing files:
Missing or failed reassign.cluster.txt files:
['gphase/gphase_output/cluster_hap/chr1/output_prefix.reassign.cluster.txt',
'gphase/gphase_output/cluster_hap/chr17/output_prefix.reassign.cluster.txt']
I understand that building a workflow capable of running on every dataset is not an easy task, but I believe the authors should improve the robustness of GPhase. The adoption of a bioinformatics tool depends not only on the quality of its results but also on its usability and its ability to deliver consistent performance in the hands of users, not just developers. Improving documentation, error handling, and installation flexibility would significantly enhance the accessibility of the tool.

Minor points:
-L58: "...with an estimated 30–80% of extant species being polyploid." The range 30–80% is quite broad and therefore not very informative. I suggest removing this sentence or adding a supporting citation.
- L74: When referring to gamete binning, the authors should cite the following articles:
https://pubmed.ncbi.nlm.nih.gov/33372615/ and https://pubmed.ncbi.nlm.nih.gov/35241824/.
- L127: "...each corresponding to a chromosome". Please add the word ideally, as this is the expected, but not guaranteed, outcome.
- L211: Please add a citation for the two rice genome datasets.
- In the figure showing BUSCO scores, the authors should indicate in the legend that each bar represents a haplotype. Additionally, they should clearly describe how each haplotype was constructed. For example, is the first haplotype composed of all the longest chromosomes?
- Figure S7: ALLHiC seems to perform quite well — not necessarily in its raw output, but in its ability to generate an assembly that can be more easily curated manually. It merges several chromosomes, but these issues are relatively straightforward to correct during manual curation. Moreover, it has the advantage of showing a lower proportion of unplaced sequences compared to GPhase and HapHiC.

(Remarks on code availability)

Reviewer #2

(Comments for the Author)
The manucript from Du et al. presents GPhase, a Hi-C–guided phasing/scaffolding tool designed for polyploid assemblies using the assembly graph and Hi-C. The authors well acknowledge the shortcomings of Hi-C (Line 83-90), and their benchmarks suggest that GPhase outperforms two other Hi-C tools. However, my main concern is: does GPhase with Hi-C alone enable good-quality haplotype-resolved polyploid genomes, relative to other modern strategies?

The first part of the concern lies in the advanced sequencing strategies. As the authors introduced (Lines 71–78), there are many strategies to assign assembled contigs to different haplotypes. Some of them (such as gamete-based approaches) are labor-intensive, while others (such as Pore-C and Omni-C) are currently more expensive than traditional Hi-C. However, I doubt that Hi-C represents a true "balance of cost, scalability, and effectiveness" (Line 78). In particular, the quality of the three polyploid genomes cited here (refs. 17–19) is not as good as that of the later compared polyploid rose and potato genomes, which used Omni-C and pollen sequencing, respectively. Therefore, in my view, the authors need to demonstrate that GPhase together with Hi-C can actually improve the quality of polyploid genome assemblies. In particular, it would be valuable to include direct comparisons between GPhase assemblies generated with Hi-C and those using more advanced proximity-ligation strategies such as Omni-C, to clarify whether the proposed method can generalize across different data types and to what extent Hi-C remains competitive. The authors could also provide practical cost–benefit evidence to justify the claim that Hi-C offers the best balance among existing options.

The second part of my concern is about the evaluation approach used in the current manuscript. One main conclusion I can draw from these evaluations is that GPhase performs better than the other two Hi-C tools, but, as explained above, this does not necessarily suggest that GPhase can lead to high-quality polyploid genome assemblies. Based on the current evaluations, it is difficult to assess whether GPhase indeed performs better in polyploid genome assembly. I list my specific questions below:

1. Using the four cultivated rice genomes as a benchmark is a great idea. However, selecting two indica and two japonica

cultivars cannot represent an autotetraploid genome, especially since the heterozygosity is 1.87%. I would consider this more like an allotetraploid genome, and grasses tend to have higher substitution rates as well. In general, one would expect Hi-C to perform better in allopolyploids than in autopolyploids. Therefore, I suggest that the authors benchmark rice cultivars with different genetic distances, which would show to what extent GPhase (and Hi-C) can handle autopolyploid genomes. Alternatively, simulations of genomes with controlled levels of divergence (from near-identical to highly diverged haplotypes) could be used to test where Hi-C–based phasing starts to fail, providing practical guidance for the community.

2. As a general strategy to compare genome assemblies, I would recommend focusing on DNA-level comparisons rather than gene-order comparisons (as shown in Figs. 2A, 4A–D, and 5A), since repetitive gene sequences also need to be evaluated in the final assembly. Also, in Fig. 3C (same for later benchmarking), because the same data were used, the null hypothesis should be that the assembled genome is identical; however, the authors did not adequately discuss the differences between the “polyploid” assembly and the ground-truth assemblies. It would always be helpful to include dot plots or alignment-based comparisons at the DNA level to visualize structural differences and to quantify the number and size of mismatches or mis-joins relative to the ground truth. Only one or two correct assembly cases (lke in Fig. 5D) cannot prove the improvment of assembly.

3. For benchmarking on the two real polyploid genomes, the authors again did not compare their assembly with haplotype genomes at the DNA level directly but instead compared gene orders with a reference genome (and in some cases it is unclear where the reference genome comes from). In addition, other technologies besides Hi-C were used to assemble these two genomes, so it is not entirely clear how the authors selected data from the original publications for their benchmarking. Please clarify which datasets (including sequencing platforms, coverage, and chemistry) were used from the original publications, and whether additional data such as Omni-C or long-read data were excluded to emulate a Hi-C–only scenario. This clarification is essential to interpret the results fairly.

4. The authors provided two assembly cases for the wax apple and rose genomes. Again, the results show that GPhase appears to perform better than HapHiC and ALLHiC (as expected) but provide little evidence that the newly assembled genomes are high-quality haplotype-resolved assemblies.

5. Lastly, in the discussion, the authors may also discuss if GPhase would also integrate other proximity data (Omni-C, Pore-C) in future versions? And if it is worth it?

Minor:
Here are just a few things I spotted.
1. Lines 59–61: The low percentage of polyploid genomes alone does not necessarily indicate the need for a new genome assembly tool.
2. Fig. 2E: The legend specifies chromosome 11, but the figure appears to show chromosome 3.
3. Line 178: low homology or high homology?
4. Lines 211 and 220: No references are provided for 9311 and FL478.

(Remarks on code availability)

Version 1:

Reviewer comments:

Reviewer #1

(Comments for the Author)
The authors have now addressed all my main comments, and the article is now suitable for publication. I would like to congratulate the authors for providing such a valuable tool, which will help the community build high-quality genomes for polyploid species.

I retested the gphase workflow on my pentaploid species and must admit that the installation process is now much easier. However, I still encountered an issue during the final stage of the workflow, specifically during the merge step. The following error message appeared: "/tmp/tmp7kqf377r: line 3: /app/scaffold_hap/../src/HapHiC/haphic: No such file or directory". I will report this issue on the GitHub repository, as I believe it should be straightforward to resolve.

(Remarks on code availability)
I was able to easily install the gphase tool and run it on my own dataset. However, the workflow crashed during the final step, which may indicate that it is not yet sufficiently robust and requires further improvement. While the code and datasets provided on Code Ocean worked well, this issue arose when using my own data.

Reviewer #2

(Comments for the Author)
The revised manuscript from Du et al. has been much improved by integrating various types of 3C data and solid DNA-level comparisons with simulated and well-assembled polyploid genomes. Therefore, my previous main concern has been largely

resolved in this version.

Below are a few remarks I think the manuscript could benefit from before publication.

1. Most importantly, I attempted to run the tool but encountered issues with environmental settings and missing scripts in the pipeline, preventing me from testing it on a polyploid genome in-house.

2. As pointed out by Reviewer 1, Hifiasm can also make use of Hi-C data mapped to the assembly graph to produce phased assemblies. Although this functon(as well as Hifiasm in general, as acknowledged by the authors) can only be applied to diploid genomes, the authors should compare their tool with Hifiasm with Hi-C (with option -h1 -h2), since both use the same concept of Hi-C scaffolding assisted by the assembly graph. Furthermore, a polyploid scaffolding tool should be able to handle diploid genomes as well.

3. Since the authors have mentioned in their rebuttal that making use of the assembly graph is central to the tool, I think they should cite that Hifiasm has also implemented the same idea, but that GPhase can apparently be applied to polyploid genomes. This would be an added value worth highlighting.

4. In my opinion, the current title and introduction do not sufficiently reflect the use of the assembly graph, so it would be better if the authors could rephrase them accordingly. That said, the discussion is quite clear, e.g., the sentence at Lines 560–562 is a good summary of this contribution.

5. Fig. 1 does not serve as a clear overview of the algorithm, and the legend is too concise to explain the figure. I think the authors should put more effort into making it accessible to a wide audience. Specifically, Fig. 1B has no explanation for the colors, numbers, and other visual elements.

6. Fig. 3 has varying scales for the y-axes. It would be helpful to use the same scale where possible, to better reflect the differences among the three scaffolding tools.

7. Line 61: the newly added word "ancient" is not accurate. Most if not all extant angiosperms are paleopolyploids, so this qualifier does not meaningfully narrow the scope. Please double-check the citation and the cited number.

8. Line 633–634: "In addition, non-3C data types, such as gamete sequencing data, may also be integrated in the future." This sentence is speculative without a concrete path forward. Consider removing it or elaborating on how this integration might be achieved.

(Remarks on code availability)
See my remark on this in the main comments.

Version 2:

Reviewer comments:

Reviewer #1

(Comments for the Author)

(Remarks on code availability)
The authors have addressed the issue reported in my previous review, and I was able to successfully run the workflow on my pentaploid dataset.

I did not have sufficient time to perform an in-depth assessment of the resulting assembly and phasing quality. However, the workflow now runs smoothly, and the results appear promising based on a first inspection.

Overall, I believe the authors have satisfactorily addressed the reviewers' comments, and I support publication of the manuscript. I would again like to congratulate the authors for developing and maintaining a valuable tool that will help the community generate high-quality phased assemblies for polyploid species.

Reviewer #2

(Comments for the Author)
In the revision, the authors have addressed all my previous remarks. I have also tested the tool with two polyploidy species sequenced in house and it can run smoothly in the end. Based on the results, I again think this tool is a valuable contribution to the community.

(Remarks on code availability)
I did not review the code line by line but installed the tool and ran it on a dataset in house. In general, it was straightforward to install in the current round of revision, and after fixing some issues, we could run the code and get the results.

Reviewer #3

(Comments for the Author)

(Remarks on code availability)

Open Access This Peer Review File is licensed under a Creative Commons Attribution 4.0 International License, which permits use, sharing, adaptation, distribution and reproduction in any medium or format, as long as you give appropriate credit to the original author(s) and the source, provide a link to the Creative Commons license, and indicate if changes were made.
In cases where reviewers are anonymous, credit should be given to 'Anonymous Referee' and the source.
The images or other third party material in this Peer Review File are included in the article's Creative Commons license, unless indicated otherwise in a credit line to the material. If material is not included in the article's Creative Commons license and your intended use is not permitted by statutory regulation or exceeds the permitted use, you will need to obtain permission directly from the copyright holder.
To view a copy of this license, visit https://creativecommons.org/licenses/by/4.0/

Reviewers' Comments to Authors:

Reviewer: 1

I read the article with great interest and would like to congratulate the authors on developing a very interesting tool and providing a thorough benchmark of its performance. The manuscript describes a bioinformatic tool dedicated to the scaffolding of polyploid genomes, a task that remains challenging even with the availability of long-read and long-range technologies. Polyploid genomes can result from different evolutionary scenarios, and their histories may have shaped genomic architectures that include both regions with little or no homozygosity between haplotypes and, conversely, regions that are sufficiently divergent to be clearly distinguished. This mosaic pattern of genomic regions makes the generalization of polyploid genome assembly particularly difficult. Although this issue is not specific to plant genomes (even if polyploidy is more common in this phylum), I was somewhat surprised that the authors did not submit their work to a more generalist journal within the Nature portfolio.

Response:

We thank the reviewer for the recognition of and positive assessment of our work. Polyploid genome assembly remains a highly challenging problem and is far from being fully resolved. Although polyploidy is not restricted to plants, polyploid species are considerably more prevalent in plants than in animals, and many of the most pressing methodological challenges in this area arise from complex plant genomes. Moreover, two widely used algorithms in this field, ALLHiC and HapHiC, were both published in *Nature Plants*, making this journal a natural and appropriate venue for presenting the GPhase algorithm.

Although the method and the tool are promising and make me enthusiastic, I should note that the manuscript could be improved before publication.

Response:

We thank the reviewer for these valuable suggestions. Our responses to each point are provided below.

The manuscript is rather long and contains a fair amount of redundancy, particularly in the benchmarking section. The evaluation of the results produced by the three tools (GPhase, ALLHiC, and HapHiC) is repeated for each dataset, which feels somewhat redundant. I suggest that the authors consider moving the detailed figures (dot plots, Hi-C maps, BUSCO scores, K-mer analyses) to the Supplementary Data and include instead a single figure summarizing the results across the five datasets. While all the reported metrics are valuable, dot plots and Hi-C maps have limited utility for comparing methods, as they rely mainly on visual inspection. A comparison based on the proportion of collapsed regions and haplotype mixing would likely be more informative and impactful.

Response:

We fully agree with the reviewer that the benchmarking section in the previous version of the manuscript contained redundancy. To address this issue, we have substantially reorganized the structure of the Results section in the revised manuscript. Specifically, we have selectively merged several figures and moved a considerable portion of the evaluation results, including dot plots, Hi-C contact maps, BUSCO scores, and *k*-mer-based analyses, to the Supplementary Figures. To further reduce repetition across datasets, we now present different types of results and use distinct visualization strategies in the figures corresponding to different datasets, rather than displaying identical analyses in the same format for each dataset.

While we agree that quantitative metrics are essential for method comparison, we believe that visual inspections such as dot plots and Hi-C maps provide an intuitive and complementary perspective that allows readers to directly perceive the differences in assembly quality among methods. In particular, these visualizations enable straightforward identification of error patterns and assembly characteristics produced by different tools—features that are often difficult to capture using summary metrics alone. For this reason, we have retained a limited number of representative dot plots and Hi-C maps in the main text, while moving the remaining ones to the Supplementary Figures.

In addition, we have substantially expanded our analyses using simulated and semi-simulated datasets to enable a more systematic comparison of tool performance across genomes with different levels of heterozygosity and collapse rates. First, we introduced a semi-simulated triploid genome constructed from three *japonica* rice genomes, which exhibits lower heterozygosity and a higher collapse rate compared with the previously used tetraploid genome constructed from two *indica* and two *japonica* genomes. Together, these two datasets allow comparisons across distinct collapse-rate regimes. Furthermore, we generated four fully simulated tetraploid genomes with varying levels of sequence divergence and conducted comprehensive comparative analyses. Across all these datasets, GPhase consistently outperformed existing methods, and its advantage became increasingly pronounced as genome complexity increased and the assembly task became more challenging (**Fig. 2-3**, **Supplementary Fig. 6-14**).

In my opinion, the stepwise validation of GPhase is not very informative and could be removed or moved to the Supplementary Information. Indeed, obtaining reliable ground-truth information is not straightforward, particularly as mentioned by the authors with the misclassification issue in RagTag (L316). Another example is the statement that the graph partitioning results produced by GPhase “closely match” the ground truth. The term closely is vague and not quantitative, and, when examining Figure 3A, readers will likely find it difficult to perceive clear differences between the GPhase graph partitioning and the ground truth.

Response:

We thank the reviewer for this valuable suggestion. As requested, we have moved the stepwise

validation analyses to Supplementary Notes. We fully acknowledge that the ground truth constructed for each intermediate step in the stepwise validation is not perfectly accurate. However, we note that such inaccuracies occur only in a limited number of cases, and that, overall, these ground-truth datasets remain sufficiently reliable for evaluating the performance of individual components of GPhase. For this reason, we have retained the stepwise validation in the Supplementary Notes rather than removing it entirely.

Regarding Figure 3A in the original manuscript, we emphasize that this figure was intended to provide a high-level, qualitative overview of the graph partitioning results, allowing readers to gain an intuitive understanding of the phasing outcome. The apparent difficulty in visually distinguishing the GPhase partitioning from the ground truth in this figure in fact reflects the high accuracy of the GPhase graph partitioning. More detailed and quantitative evaluations of partitioning accuracy are provided in Figure 3B of the original manuscript, which explicitly reports the relevant metrics.

The comparison between the two genome assemblies of the autotetraploid wax apple is, in my opinion, out of scope. For example, the differences observed for Chromosomes 5 and 6–11 are mainly due to the fact that the first version was not properly manually curated. The authors should therefore move this comparison to the Supplementary Data and focus the main manuscript on the comparison of polyploid scaffolding methods.

Response:

We fully agree that the focus of the manuscript should be on the comparison among polyploid scaffolding methods. As requested, we have moved the detailed figures related to chromosomes 5 and 6–11 of the wax apple genome to the Supplementary Figures (**Supplementary Fig. 41**).

Likewise, I would expect some evaluation of how a de novo assembly generated with Hifiasm followed by YaHS scaffolding performs, as this would be of interest to readers. Since this is a common approach, users need to understand the impact of using a non-dedicated scaffolding strategy. More importantly, Hifiasm is theoretically able to handle polyploid genomes when using Hi-C data, one can provide the Hi-C data as input and specify the expected ploidy using the n_hap parameter. It would therefore be useful to know how this method performs in comparison to GPhase. Generally, to estimate ploidy, researchers rely on the Smudgeplot tool. I think it would again be useful for readers to see, through these concrete examples, how accurate Smudgeplot's predictions are.

Response:

We thank the reviewer for this valuable suggestion. As requested, we evaluated the performance of the hifiasm + YaHS pipeline on both the tetraploid rose and tetraploid wax apple datasets. In both cases, this strategy performed poorly compared with the methods evaluated in our study (**Fig. 4-5**, **Supplementary Fig. 19-20**, **Supplementary Fig. 23** and **Supplementary Fig. 40**).

For the tetraploid rose genome, the assembly produced by hifiasm + YaHS exhibited a total assembly size that was substantially larger than the expected genome size. This issue was not caused by the YaHS-based scaffolding step but rather originated from the phased contig sets generated by hifiasm itself, whose total length (3.82 Gb) already far exceeded the expected genome size (1.97 Gb). Upon closer inspection, although contigs were partitioned into four groups (as *n_hap* was set to 4), many chromosomes contained more than one haplotype representation within each group, which directly led to the inflated assembly size (**Supplementary Fig. 19**, **Supplementary Fig. 23** and **Supplementary Table 5**).

In the tetraploid wax apple genome, while the total assembly size was not excessively inflated, hifiasm assigned the vast majority of sequences to a single haplotype, leaving the remaining three haplotypes with only a very small fraction of the total sequence. This resulted in an extremely unbalanced phasing outcome (**Supplementary Table 6**).

To our knowledge, although hifiasm theoretically provides support for polyploid phasing, this functionality has not yet been widely adopted in practice. In recent years, de novo polyploid genome assemblies that leverage chromatin conformation capture data have predominantly relied on tools such as ALLHiC and HapHiC. Moreover, the main developers of hifiasm have repeatedly acknowledged, through GitHub discussions and conference presentations, the instability of its polyploid phasing module. These observations are consistent with our empirical results on the rose and wax apple datasets.

Regarding the Smudgeplot-based ploidy estimation, we used Smudgeplot to infer the ploidy of the wax apple genome, which was predicted to be tetraploid (**Supplementary Fig. 39**). This result is consistent with previous reports in the literature and also agrees with our assembly results, supporting the accuracy of the Smudgeplot-based ploidy prediction.

In Figures 2E and S5, there are many white bins. The authors should clarify whether these correspond to regions resulting from haplotype mixing (which could indicate a limitation of the method) or to regions that cannot be distinguished between haplotypes. I assume both cases are represented, but the authors should clearly specify what these white bins represent and, ideally, use additional colors in the figures to distinguish between the two situations.

Response:

We thank the reviewer for pointing this out. The white bins correspond to regions dominated by *k*-mers that appear in more than one haplotype in the ground truth. In other words, these regions are overly similar among haplotypes in the ground truth, making it difficult to evaluate the phasing accuracy of GPhase using this visualization. Therefore, the white bins represent regions that cannot be effectively assessed by this method, rather than regions where our algorithm performs

poorly. We acknowledge the limitations of this visualization approach. We have also clarified this point in the figure legend, and in the revised manuscript, we have moved this figure to the Supplementary Figures (**Supplementary Fig. 14**).

When trying to download the assemblies generated for the benchmark datasets, I was unable to find the complete set of assemblies, as only the final ones for wax apple and China rose were available. Please, provide readers with access to all assemblies generated in the study, along with a supplementary table listing standard assembly metrics and the accession numbers for each.

Response:

We thank the reviewer for this valuable suggestion. As requested, all assemblies generated by the tools included in the comparison have been deposited in Hugging Face, along with their basic assembly metrics and direct download links, which are provided in **Supplementary Table 7**. The raw sequencing data for the newly generated datasets, as well as the public datasets used in this study, along with their access links and basic metadata, are summarized in **Supplementary Table 3**. For each dataset used to evaluate GPhase and other scaffolding tools, the reference sequences or ground truth genome assemblies are listed with their download links in **Supplementary Table 2**. Furthermore, the final assemblies of wax apple and Chinese rose remain available at **PRJCA045971**, and all newly sequenced data have been successfully uploaded and made publicly accessible. The simulated HiFi and Hi-C reads for the fully simulated genomes were not uploaded due to their large file size; however, detailed information regarding the simulation process is provided in **Supplementary Note 3**.

More importantly, I tested the program on my own dataset. First, the installation requires access to a repository that provides very little information and is entirely written in Chinese, which does not inspire confidence for end users. I recommend that the authors distribute a containerized version of the tool (for example, using Apptainer) to simplify installation, since not all users have unrestricted access to Conda. The workflow also includes a pipeline dedicated to the initial step (gphase popcnv …), but the script does not seem to account for this workflow; the authors should address this issue.

Response:

We sincerely thank the reviewer for their constructive and insightful suggestions, which are invaluable for enhancing the credibility and usability of the tool. We apologize for the issues raised regarding the code repository and have taken immediate action. A detailed usage guide has now been added to the GitHub repository, covering installation steps, parameter explanations, and input/output descriptions. Additionally, necessary English comments have been added to the key code sections to ensure that users worldwide can accurately understand the algorithm's logic.

Regarding installation and deployment, we fully agree with the reviewer's concern about the limitations of Conda environments. In response, we have specifically built and released a Docker

image, which significantly enhances the tool's portability, especially in complex environments such as high-performance computing clusters (HPC). Furthermore, we have addressed the logical issue with the GPhase popcnv step in the main program and completed the necessary code fixes, ensuring that this module is correctly called.

All updates have been synchronized to the GitHub repository, and the updated workflow has been rigorously tested and validated on datasets of varying sizes to ensure stability and robustness. We are confident that these improvements will significantly optimize the user experience.

Finally, I launched the tool on a pentaploid genome, and only the first step (popCNV) completed successfully. The GPhase workflow crashed, apparently due to a clustering error for chromosomes 1 and 17, resulting in missing files: Missing or failed reassign.cluster.txt files: ['gphase/gphase_output/cluster_hap/chr1/output_prefix.reassign.cluster.txt', 'gphase/gphase_output/cluster_hap/chr17/output_prefix.reassign.cluster.txt'] I understand that building a workflow capable of running on every dataset is not an easy task, but I believe the authors should improve the robustness of GPhase. The adoption of a bioinformatics tool depends not only on the quality of its results but also on its usability and its ability to deliver consistent performance in the hands of users, not just developers. Improving documentation, error handling, and installation flexibility would significantly enhance the accessibility of the tool.

Response:

We sincerely apologize for GPhase's suboptimal performance on the pentaploid genome. To improve the effectiveness and robustness of both the algorithm and its software implementation, we have undertaken several key improvements: 1) Enhanced Software Robustness: We strengthened the handling of invalid or unexpected inputs across different steps and modules. As a result, even when upstream steps produce suboptimal or invalid intermediate results, the downstream modules are less likely to crash and can still generate meaningful outputs. 2) Algorithm Refinement: We made several adjustments to the chromosome and haplotype clustering steps, improving the clustering process and enhancing the algorithm's overall robustness. 3) Cross-Platform Testing: GPhase was tested on multiple server platforms, and platform-specific issues were identified and resolved to further improve the software's reliability. 4) Broader Polyploid Genome Evaluation: We further expanded the evaluation of GPhase beyond the primary benchmark datasets to a broader range of polyploid genomes, including, but not limited to, the hexaploid *Diospyros kaki* 'Xiaoguotianshi', the tetraploid *Medicago sativa* 'Xinjiangdaye', the tetraploid *Ziziphus mauritiana* 'Xishuangyesheng', and the tetraploid *Medicago sativa* 'Zhongmu-4' genomes. The results of these additional experiments have been incorporated into the revised manuscript (**Supplementary Fig. 35-38**). In addition, independent researchers have applied GPhase to their own genome datasets, including tetraploid rose and triploid pear species, achieving favorable results. Furthermore, all example datasets provided with GPhase have been successfully executed on the official Code Ocean platform. After implementing these

improvements and conducting extensive testing, we believe GPhase's robustness has been significantly enhanced, and it is now capable of producing valid outputs and high-quality assemblies for most polyploid genomes.

Nevertheless, given the diversity and complexity of polyploid genomes, there may still exist edge cases that have not yet been fully considered. Therefore, we would greatly appreciate it if the pentaploid genome and the corresponding raw data could be provided in an anonymized form, so that we can further test and improve GPhase. We believe that this would be highly beneficial for further enhancing the robustness and performance of the method.

Minor points:

-L58: "...with an estimated 30–80% of extant species being polyploid." The range 30–80% is quite broad and therefore not very informative. I suggest removing this sentence or adding a supporting citation.

Response:

We have removed this sentence from the manuscript.

- L74: When referring to gamete binning, the authors should cite the following articles: https://pubmed.ncbi.nlm.nih.gov/33372615/ and https://pubmed.ncbi.nlm.nih.gov/35241824/.

Response:

We have cited these articles in the revised manuscript.

- L127: "...each corresponding to a chromosome". Please add the word ideally, as this is the expected, but not guaranteed, outcome.

Response:

We have added "ideally" in this sentence.

- L211: Please add a citation for the two rice genome datasets.

Response:

We have added the citations for the two rice genome datasets.

- In the figure showing BUSCO scores, the authors should indicate in the legend that each bar represents a haplotype. Additionally, they should clearly describe how each haplotype was constructed. For example, is the first haplotype composed of all the longest chromosomes?

Response:

We thank the reviewer for pointing this out. We have clarified in the legend of each BUSCO-related figure that each bar represents one haplotype, and we have further explained the haplotype construction procedure in the 'Validation' section in Methods. Specifically, all assembled chromosome sequences were aligned to the ground-truth genome, and for each chromosome

sequence, the best-matching haplotype in the ground truth was identified. The assembly haplotypes were then constructed based on this one-to-one matching strategy. We note that the haplotypes in the ground-truth genomes were generated by the original authors using different approaches, such as leveraging parental information or predefined haplotype assignments.

- Figure S7: ALLHiC seems to perform quite well — not necessarily in its raw output, but in its ability to generate an assembly that can be more easily curated manually. It merges several chromosomes, but these issues are relatively straightforward to correct during manual curation. Moreover, it has the advantage of showing a lower proportion of unplaced sequences compared to GPhase and HapHiC.

Response:

We thank the reviewer for pointing this out.

First, we clarify that the observed difference mainly reflects how different tools handle unplaced sequences, rather than a genuine difference in their amount. In the ALLHiC assembly, unplaced sequences are not absent from individual chromosomes; instead, sequences that cannot be confidently phased are appended to the end of each chromosome. As shown in **Supplementary Fig. 27** in the revised manuscript (the same figure as Supplementary Fig. 7 in the original manuscript, now including explicit annotation of unplaced sequences), contact maps of all chromosomes contain matrices of varying sizes at their ends, corresponding to these unplaced sequences. In contrast, GPhase and other tools place all such unplaced sequences together at the end of the assembly. Overall, the amount of unplaced sequences in ALLHiC is not smaller than that in GPhase.

Second, while we acknowledge the reviewer's perspective, we respectfully hold a different view regarding the manual curatability of the ALLHiC assembly. In our evaluation, ALLHiC does not achieve effective phasing for most chromosomes, resulting in sequences from different haplotypes being grouped together and often arranged in an incorrect order. Under these conditions, we find that performing reliable manual curation is challenging in practice, particularly when haplotype resolution is required.

Reviewer: 2

The manucript from Du et al. presents GPhase, a Hi-C–guided phasing/scaffolding tool designed for polyploid assemblies using the assembly graph and Hi-C. The authors well acknowledge the shortcomings of Hi-C (Line 83-90), and their benchmarks suggest that GPhase outperforms two other Hi-C tools. However, my main concern is: does GPhase with Hi-C alone enable good-quality haplotype-resolved polyploid genomes, relative to other modern strategies?

Response:

We thank the reviewer for this very valuable and insightful comment. We would like to emphasize that the central innovation of this work is the introduction of assembly graph information into polyploid genome scaffolding. This conceptual advance is not limited to Hi-C–based haplotype-resolved scaffolding but is equally applicable to scaffolding approaches based on other types of chromatin conformation capture data. Accordingly, the core steps of the GPhase algorithm are largely independent of the specific 3C data type used, and only minor modifications in the data preprocessing stage are required to extend GPhase to other technologies such as Pore-C and Omni-C.

To demonstrate this generality, we extended GPhase to support polyploid haplotype-resolved scaffolding using both Pore-C and Omni-C data. Benchmarking on Pore-C and Omni-C datasets shows that GPhase continues to substantially outperform state-of-the-art algorithms across these different chromatin conformation capture modalities, including methods specifically developed for Pore-C data. These results provide strong evidence for the broad utility of assembly graph integration and the robustness of the GPhase framework. In line with this broader scope, we have revised the manuscript title to "GPhase: Haplotype-resolved Polyploid Genome Scaffolding via Integration of Chromatin Conformation Capture Data and Assembly Graph."

We also note that the limitations discussed in the manuscript are not specific to Hi-C data, but apply more generally to chromatin conformation capture technologies as a whole. Although newer data types such as Pore-C and Omni-C partially mitigate some of these limitations through longer read lengths and reduced dependence on restriction enzyme sites, all chromatin interaction data fundamentally provide coarse-grained linkage information. As such, they cannot fully resolve challenges arising from short unitigs or collapsed regions. In contrast, the assembly graph encodes fine-grained long-read overlap information, which effectively complements chromatin interaction data and substantially enhances polyploid genome assembly performance when the two are integrated.

We acknowledge that experimental approaches combining wet-lab techniques with sequencing, such as pollen sequencing, can further improve polyploid genome assembly quality. However, these methods typically require labor-intensive and time-consuming experimental procedures, and are applicable to only a limited number of species, which constrains their broader adoption.

Therefore, we believe that the use of pollen sequencing data is unlikely to represent the mainstream direction of future polyploid genome assembly. Moreover, pollen sequencing also provides relatively coarse-grained linkage information and is therefore conceptually complementary rather than contradictory to the fine-grained information captured by assembly graphs. Although GPhase does not currently support pollen sequencing data, future work—by us or by others—may explore the integration of assembly graph information with such experimental data to further improve polyploid genome assembly.

In summary, the key contribution of this work is the integration of assembly graph information into polyploid genome scaffolding, a strategy whose applicability extends beyond Hi-C and even beyond chromatin conformation capture data. We believe that assembly graph–based approaches will play an increasingly important role in future polyploid genome assembly methods. Consistent with this view, the concept of incorporating assembly graph information into polyploid scaffolding has already begun to attract attention in the community, and we are aware of ongoing efforts to introduce similar ideas into the next generation of existing tools.

The first part of the concern lies in the advanced sequencing strategies. As the authors introduced (Lines 71–78), there are many strategies to assign assembled contigs to different haplotypes. Some of them (such as gamete-based approaches) are labor-intensive, while others (such as Pore-C and Omni-C) are currently more expensive than traditional Hi-C. However, I doubt that Hi-C represents a true "balance of cost, scalability, and effectiveness" (Line 78). In particular, the quality of the three polyploid genomes cited here (refs. 17–19) is not as good as that of the later compared polyploid rose and potato genomes, which used Omni-C and pollen sequencing, respectively. Therefore, in my view, the authors need to demonstrate that GPhase together with Hi-C can actually improve the quality of polyploid genome assemblies. In particular, it would be valuable to include direct comparisons between GPhase assemblies generated with Hi-C and those using more advanced proximity-ligation strategies such as Omni-C, to clarify whether the proposed method can generalize across different data types and to what extent Hi-C remains competitive. The authors could also provide practical cost–benefit evidence to justify the claim that Hi-C offers the best balance among existing options.

Response:

We thank the reviewer for these valuable comments. As most of the points raised have already been addressed in our response to the previous comment, we provide additional clarification on several specific details below.

We acknowledge that it was not entirely accurate to claim that Hi-C alone represents a balance of cost, scalability, and effectiveness. Since we have extended GPhase to support multiple types of 3C data, including Hi-C, Pore-C, and Omni-C, and have revised the overall scope of the study accordingly, we have modified this statement in the revised manuscript to indicate that chromatin

conformation capture data, rather than Hi-C specifically, represent a balance of cost, scalability, and effectiveness.

As requested, we evaluated GPhase on Pore-C and Omni-C data and compared its performance with state-of-the-art haplotype-resolved scaffolding tools. First, using the tetraploid rose (*R. hybrida* ' Samantha ') genome, for which both Hi-C and Pore-C data are available, we systematically compared GPhase+Hi-C and GPhase+Pore-C with representative state-of-the-art methods, including HapHiC+Hi-C, ALLHiC+Hi-C, hifiasm+YaHS+Hi-C, HapHiC+Pore-C, and C-Phasing+Pore-C (the latter being specifically developed for Pore-C–based scaffolding). Across all evaluations, GPhase+Hi-C and GPhase+Pore-C consistently ranked among the top two methods and clearly outperformed the other approaches (**Fig. 4A-E**, **Supplementary Fig. 20-24**). Second, using one potato genome with Omni-C data, we compared GPhase with ALLHiC. At present, there are no scaffolding tools specifically designed for Omni-C data, and HapHiC could not be run successfully on the dataset. The results showed that GPhase consistently and substantially outperformed ALLHiC (**Fig. 4F-G**, **Supplementary Fig. 31-34**). Taken together, these results demonstrate the superior performance of GPhase and the effectiveness of incorporating assembly graph information across different types of chromatin conformation capture data.

Given that we have expanded the scope of this study from Hi-C to encompass chromatin conformation capture data more broadly, we believe that it is no longer necessary to specifically justify the claim that Hi-C offers the best balance among existing options.

The second part of my concern is about the evaluation approach used in the current manuscript. One main conclusion I can draw from these evaluations is that GPhase performs better than the other two Hi-C tools, but, as explained above, this does not necessarily suggest that GPhase can lead to high-quality polyploid genome assemblies. Based on the current evaluations, it is difficult to assess whether GPhase indeed performs better in polyploid genome assembly. I list my specific questions below:

Response:

We acknowledge the limitations of the evaluation approach in the previous version of the manuscript. To address these shortcomings, we have added multiple complementary evaluation methods, including both quantitative metrics and visualization-based analyses. In addition, we substantially expanded the benchmarking by incorporating a wide range of test datasets to evaluate GPhase and compare it with other tools, including semi-simulated datasets, fully simulated datasets, and real datasets. We detail these changes below.

1. Using the four cultivated rice genomes as a benchmark is a great idea. However, selecting two indica and two japonica cultivars cannot represent an autotetraploid genome, especially since the

heterozygosity is 1.87%. I would consider this more like an allotetraploid genome, and grasses tend to have higher substitution rates as well. In general, one would expect Hi-C to perform better in allopolyploids than in autopolyploids. Therefore, I suggest that the authors benchmark rice cultivars with different genetic distances, which would show to what extent GPhase (and Hi-C) can handle autopolyploid genomes. Alternatively, simulations of genomes with controlled levels of divergence (from near-identical to highly diverged haplotypes) could be used to test where Hi-C–based phasing starts to fail, providing practical guidance for the community.

Response:

We thank the reviewer for these valuable suggestions.

As requested, to further evaluate performance on polyploid genomes with lower heterozygosity (heterozygosity = 0.7%), we constructed an additional semi-simulated autotriploid genome using three *japonica* rice genomes. Consistent with our previous results, GPhase again substantially outperformed state-of-the-art tools across all evaluated metrics on this dataset (**Supplementary Fig. 7-9** and **Supplementary Fig. 11-13**).

Furthermore, we generated four fully simulated tetraploid genomes with different levels of sequence similarity (divergence rates of 3%, 1%, 0.5%, and 0.2%). The results demonstrate that as haplotype similarity increases, the performance advantage of GPhase over other tools becomes progressively more pronounced across all evaluation metrics (**Fig. 3**). These findings highlight the superior performance of GPhase on more challenging polyploid genomes with highly similar haplotypes.

Additionally, we respectfully hold a different perspective on whether the semi-simulated tetraploid genome should be classified as an allopolyploid. Although the divergence between *indica* and *japonica* rice genomes is greater than that between two *indica* genomes or two *japonica* genomes, both *indica* and *japonica* belong to the AA genome group of cultivated rice. We therefore consider the resulting semi-simulated tetraploid genome to represent an autopolyploid rather than an allopolyploid. Importantly, even in real autopolyploid genomes, the similarity among the haplotypes is unlikely to be perfectly balanced. Moreover, using *indica* and *japonica* rice genomes to construct simulated polyploid genomes has precedent in the field. For example, the ALLHiC study (Zhang *et al.*, *Nature Plants*, 2019) used one *indica* and one *japonica* genome to generate a simulated diploid genome for evaluating phasing accuracy, indicating that this design is an accepted practice.

2. As a general strategy to compare genome assemblies, I would recommend focusing on DNA-level comparisons rather than gene-order comparisons (as shown in Figs. 2A, 4A–D, and 5A), since repetitive gene sequences also need to be evaluated in the final assembly. Also, in Fig. 3C (same for later benchmarking), because the same data were used, the null hypothesis should be

that the assembled genome is identical; however, the authors did not adequately discuss the differences between the "polyploid" assembly and the ground-truth assemblies. It would always be helpful to include dot plots or alignment-based comparisons at the DNA level to visualize structural differences and to quantify the number and size of mismatches or mis-joins relative to the ground truth. Only one or two correct assembly cases (lke in Fig. 5D) cannot prove the improvment of assembly.

Response:

We fully agree that DNA-level comparisons are more informative than gene-order–based comparisons, and we acknowledge that the previous evaluation lacked sufficient validation against ground truth. To address this concern, we have added a comprehensive set of evaluations based on direct alignment between the assemblies and the ground truth. These evaluations were conducted on two semi-simulated and three real genomes, covering datasets generated with Hi-C, Pore-C, and Omni-C data.

First, we added dot plot analyses between the assemblies and the ground truth, which provide an intuitive visualization of their structural concordance and discrepancies (**Fig. 2**, **Fig. 4**, **Supplementary Fig. 8**, **Supplementary Fig. 20**, **Supplementary Fig. 22**, **Supplementary Fig. 26** and **Supplementary Fig. 31**). Second, we introduced several quantitative, sequence-alignment–based metrics, including completeness metrics such as the *missing rate* and accuracy metrics such as *number of mismatches per kb* and *mismatch rate*, enabling a rational and numerical assessment of differences between the assemblies and the ground truth (**Fig. 2D**, **Fig. 4E**, **Fig. 4G**, **Supplementary Fig. 13** and **Supplementary Fig. 30**). In addition, we evaluated the assemblies using unique *k*-mer–based metrics (including SER, IUADD, and SMR). Although these metrics are not based on direct sequence alignment, they are nonetheless DNA-level measures that effectively quantify discrepancies between assemblies and the ground truth (**Fig. 2C**, **Fig. 4D**, **Fig. 4G**, **Supplementary Fig. 12** and **Supplementary Fig. 29**). Across all datasets and all evaluated 3C data types, GPhase consistently outperformed state-of-the-art tools according to all these evaluation criteria.

Regarding Fig. 3C (in the original manuscript), we also provided corresponding sequence-alignment–based quantitative metrics, such as the missing rate, the number of mismatches per kb and the mismatch rate, to evaluate the differences between the assemblies and the ground truth (**Supplementary Table 1**).

3. For benchmarking on the two real polyploid genomes, the authors again did not compare their assembly with haplotype genomes at the DNA level directly but instead compared gene orders with a reference genome (and in some cases it is unclear where the reference genome comes from). In addition, other technologies besides Hi-C were used to assemble these two genomes, so it is not entirely clear how the authors selected data from the original publications for their benchmarking.

Please clarify which datasets (including sequencing platforms, coverage, and chemistry) were used from the original publications, and whether additional data such as Omni-C or long-read data were excluded to emulate a Hi-C–only scenario. This clarification is essential to interpret the results fairly.

Response:

We thank the reviewer for pointing this out. As mentioned in our response to the previous comment, for the benchmark on every real polyploid genome with high-quality ground-truth assembly, we have added sequence-alignment-based evaluation methods in the revised manuscript to directly assess the differences between the assemblies and the ground truth genomes. These evaluations include intuitive visualizations, such as dot plots, as well as multiple quantitative metrics derived from sequence alignments. The newly added results consistently demonstrate the superior performance of GPhase compared with state-of-the-art tools (**Fig. 4A**, **Fig. 4E-G**, **Supplementary Fig. 20**, **Supplementary Fig. 22**, **Supplementary Fig. 26**, **Supplementary Fig. 30** and **Supplementary Fig. 31**).

In addition, we aligned long reads back to the assemblies to compute the switch error rate. Notably, on the rose genome, the assemblies generated by GPhase combined with either Hi-C or Pore-C data achieved switch error rates that were very close to those of the ground truth, whereas the results produced by other tools deviated substantially from the ground truth (**Fig. 4C**). This example clearly demonstrates that GPhase can generate high-quality haplotype-resolved polyploid genome assemblies when used with either Hi-C or Pore-C data alone.

We also retained gene-based synteny plots in the main text and/or Supplementary Figures. All sources of the reference genomes used for the synteny analyses have now been clearly specified in the revised manuscript.

For each dataset, we clarified the relevant information—including sequencing platforms, sequencing coverage, the type of 3C data used, and data sources—in **Supplementary Table 2–3**. In addition, for each validation experiment, we have provided detailed descriptions of the experimental procedures in the **Methods** and **Supplementary Note 2**, explicitly specifying which datasets from the original publications were used in this study. To avoid any ambiguity, only a single type of 3C data was used for scaffolding in each benchmarking experiment. In contrast, the downloaded ground-truth genomes may have been generated using multiple types of 3C data during their original assembly processes.

4. The authors provided two assembly cases for the wax apple and rose genomes. Again, the results show that GPhase appears to perform better than HapHiC and ALLHiC (as expected) but provide little evidence that the newly assembled genomes are high-quality haplotype-resolved assemblies.

Response:

We thank the reviewer for pointing this out. In the revised manuscript, we have added a series of evaluation analyses to demonstrate the high quality of the two genome assemblies.

As no ground-truth assemblies are available, we mainly performed reference-free evaluations based on sequencing reads. First, both HiFi and short reads were aligned to the two assemblies, and assembly quality was assessed using CRAQ based on the alignment results. The obtained quality scores were 93.535 for wax apple and 90.714 for rose. According to the CRAQ documentation, assemblies with quality scores above 90 are considered high quality.

Second, we evaluated assembly completeness using KAT(**Supplementary Fig. 45-46**). For both genomes, the results indicated that the assemblies captured the vast majority of *k*-mers present in the HiFi reads, with no black peaks corresponding to missing *k*-mers, suggesting high completeness of the assemblies.

In addition, for the wax apple genome, we assessed switch errors by aligning ONT reads to the assembly, and the estimated switch error rate was 0.37%.

Taken together, these results demonstrate that GPhase, even when using only Hi-C data, is capable of producing high-quality haplotype-resolved assemblies.

5. Lastly, in the discussion, the authors may also discuss if GPhase would also integrate other proximity data (Omni-C, Pore-C) in future versions? And if it is worth it?

Response:

We thank the reviewer for this valuable suggestion. As GPhase has already been extended to support Pore-C and Omni-C data, we further discuss in the Discussion section its potential extension to other data types. These include additional forms of chromatin interaction data, such as HiFi-C, as well as complementary data sources beyond chromatin conformation capture, for example pollen sequencing.

Minor:

Here are just a few things I spotted.

1. Lines 59–61: The low percentage of polyploid genomes alone does not necessarily indicate the need for a new genome assembly tool.

Response:

We agree that there was a minor logical gap in this statement. In the revised manuscript, we have revised the text to: “However, among currently available high-quality genome assemblies, polyploid genomes account for only about 18%.” This striking discrepancy highlights the substantial challenges associated with polyploid genome assembly and underscores the need for

Reviewers' Comments to Authors:

Reviewer: 1

The authors have now addressed all my main comments, and the article is now suitable for publication. I would like to congratulate the authors for providing such a valuable tool, which will help the community build high-quality genomes for polyploid species.

Response:

We sincerely thank the reviewer for the positive evaluation and encouraging comments. We are pleased that the revisions have addressed the main concerns, and we hope that GPhase will be a useful resource for the community.

I retested the gphase workflow on my pentaploid species and must admit that the installation process is now much easier. However, I still encountered an issue during the final stage of the workflow, specifically during the merge step. The following error message appeared: "/tmp/tmp7kqf377r: line 3: /app/scaffold_hap/../src/HapHiC/haphic: No such file or directory". I will report this issue on the GitHub repository, as I believe it should be straightforward to resolve.

Response:

We apologize for this issue and thank the reviewer for identifying this important problem. It was caused by submodules not being correctly packaged in the previous software image, and this has now been fixed. In addition, as we describe in the response to the editor's comments, we have further comprehensively improved the software, including the following aspects:

1) We rebuilt a more stable and streamlined Conda environment, reducing dependency complexity and improving installation success rates. In addition, we repackaged Docker and Singularity images to facilitate usage across diverse computational environments.
2) To address runtime errors of the Pore-C Pipeline-Toolbox on large genomes (>8 Gb) and its inefficiency on smaller genomes (<8 Gb), GPhase no longer depends on this tool. We have redesigned the Pore-C data processing workflow, improving overall performance and adding support for CiFi data.
3) We added genome size estimation to the popcnv_pipeline script, improving its performance and stability when handling large genomes (>8 Gb).
4) Two new parameters (--t_len_T and --a_len_T) have been introduced in gphase_pipeline, allowing more flexible handling of cases where rDNA sequences are clustered as separate chromosomes.
5) We fixed an issue in the conversion from BAM to pairs format where self-alignments of unitigs were not retained.
6) We resolved issues in the phasing process under low or ultra-low coverage 3C data, improving the overall stability and robustness of the pipeline.
7) We enhanced the handling of large collapsed regions within chromosomes by enabling automatic duplication of unitigs according to the inferred haplotype number.
8) We implemented consistency checks for input files, automatically verifying that unitigs in GFA or copy number files fully match those in the FASTA file.

Furthermore, we are fully committed to the long-term maintenance of GPhase on GitHub. We will actively incorporate user feedback and provide continuous updates to ensure that the software remains robust, stable, and user-friendly for the broader research community.

I was able to easily install the gphase tool and run it on my own dataset. However, the workflow crashed during the final step, which may indicate that it is not yet sufficiently robust and requires further improvement. While the code and datasets provided on Code Ocean worked well, this issue arose when using my own data.

Response:

We sincerely apologize again for this issue. As noted in our response above, we have made substantial efforts to comprehensively improve the software, and we hope that the reviewers' datasets can now be successfully processed with reliable performance.

We also note that software maturation is an iterative process, and we warmly welcome feedback from the reviewers and the broader community via GitHub to further improve the robustness of GPhase. We sincerely appreciate their support.

Reviewer: 2

The revised manuscript from Du et al. has been much improved by integrating various types of 3C data and solid DNA-level comparisons with simulated and well-assembled polyploid genomes. Therefore, my previous main concern has been largely resolved in this version.

Response:

We sincerely thank the reviewer for the positive feedback. We are glad that the revisions have addressed the main concerns and greatly appreciate the reviewer's recognition of our improvements.

Below are a few remarks I think the manuscript could benefit from before publication.

1. Most importantly, I attempted to run the tool but encountered issues with environmental settings and missing scripts in the pipeline, preventing me from testing it on a polyploid genome in-house.

Response:

We apologize for this issue and thank the reviewer for identifying this important problem. It was caused by submodules not being correctly packaged in the previous software image, and this has now been fixed. To address this, the updated version of GPhase provides a more robust Conda-based installation workflow and Docker/Singularity image support. We have also streamlined the submodules by removing unnecessary components, thereby reducing dependency complexity and improving installation success rates. In addition, as we describe in the response to the editor's comments, we have further comprehensively improved the software, including the following aspects:

1) We rebuilt a more stable and streamlined Conda environment, reducing dependency complexity and improving installation success rates. In addition, we repackaged Docker and Singularity images to facilitate usage across diverse computational environments.
2) To address runtime errors of the Pore-C Pipeline-Toolbox on large genomes (>8 Gb) and its inefficiency on smaller genomes (<8 Gb), GPhase no longer depends on this tool. We have redesigned the Pore-C data processing workflow, improving overall performance and adding support for CiFi data.
3) We added genome size estimation to the popcnv_pipeline script, improving its performance and stability when handling large genomes (>8 Gb).
4) Two new parameters (--t_len_T and --a_len_T) have been introduced in gphase_pipeline, allowing more flexible handling of cases where rDNA sequences are clustered as separate chromosomes.
5) We fixed an issue in the conversion from BAM to pairs format where self-alignments of unitigs were not retained.
6) We resolved issues in the phasing process under low or ultra-low coverage 3C data, improving the overall stability and robustness of the pipeline.
7) We enhanced the handling of large collapsed regions within chromosomes by enabling automatic duplication of unitigs according to the inferred haplotype number.
8) We implemented consistency checks for input files, automatically verifying that unitigs in GFA or copy number files fully match those in the FASTA file.

Furthermore, we are fully committed to the long-term maintenance of GPhase on GitHub. We will actively incorporate user feedback and provide continuous updates to ensure that the software remains robust, stable, and user-friendly for the broader research community.

2. As pointed out by Reviewer 1, Hifiasm can also make use of Hi-C data mapped to the assembly graph to produce phased assemblies. Although this functon (as well as Hifiasm in general, as acknowledged by the authors) can only be applied to diploid genomes, the authors should compare their tool with Hifiasm with Hi-C (with option -h1 -h2), since both use the same concept of Hi-C scaffolding assisted by the assembly graph. Furthermore, a polyploid scaffolding tool should be able to handle diploid genomes as well.

Response:

We agree that hifiasm adopts a similar assembly graph-based strategy for diploid genome assembly, and in principle, polyploid phasing algorithms can also be applied to haplotype-resolved assembly of diploid genomes. In the manuscript, we have already provided comparisons between GPhase and hifiasm + YaHS on polyploid datasets, showing that the phasing performance of hifiasm is limited in polyploid settings.

To further compare GPhase with hifiasm + YaHS on diploid genomes, we have added two new experiments using a diploid kiwifruit genome and an unpublished diploid orchid genome. On the kiwifruit dataset, the assembly quality of GPhase is slightly lower than that of hifiasm + YaHS, but remains comparable overall(Gene-based synteny result as R2R Figure 1). On the orchid dataset, which lacks a ground truth or closely related reference genome, the two haplotypes assembled by GPhase achieved higher BUSCO completeness scores, indicating more complete assemblies and potentially more accurate phasing(details as R2R Table 1). Overall, based on these limited datasets, we believe that GPhase can achieve phasing performance comparable to hifiasm in diploid genome assembly.

R2R Table 1 Performance comparison of GPhase with hifiasm+YaHS on the orchid dataset

	GPhase		hifiasm+YaHS	
	Hap1	Hap2	Hap1	Hap2
BUSCO(%)	89.6(S：87.4)	90.3(S：86.8)	83(S：54.9)	84.3(S：57.4)
Total length(Gb)	2.47	2.47	2.79	2.79
Map rate(%)	99.73		99.76	
Coverage rate(%)	99.97		99.78	

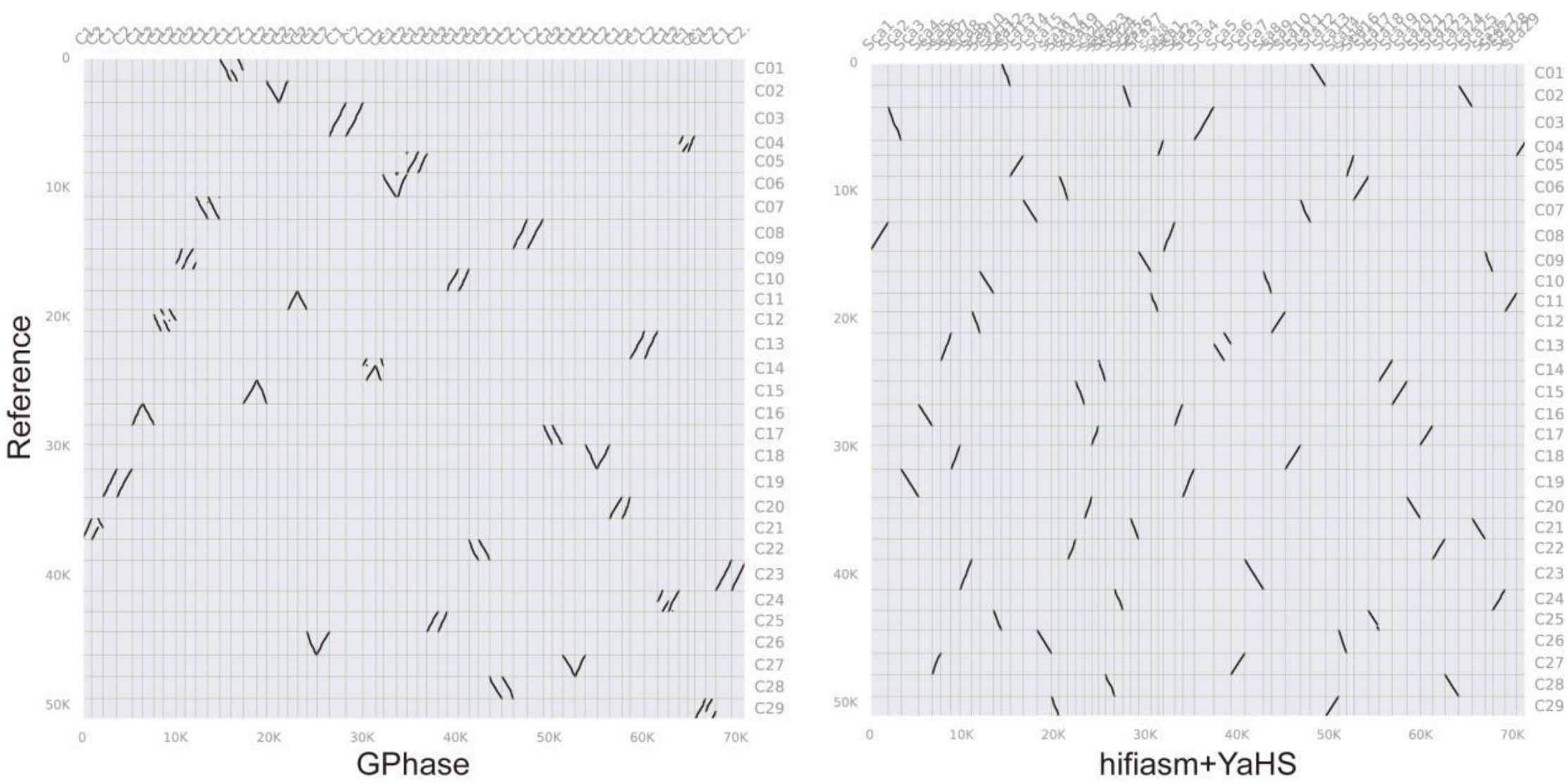

R2R Figure 1. Gene-based synteny plots of chromosome-level kiwifruit genome assemblies generated by the two methods. The horizontal and vertical axes represent the assembly and the reference genome, respectively.

Nevertheless, we emphasize that GPhase is primarily designed for haplotype-resolved assembly of polyploid genomes. For diploid genomes, we still recommend the use of hifiasm for phasing. This recommendation is based on two considerations: (1) GPhase has not yet been extensively benchmarked on diploid genomes; and (2) polyploid phasing algorithms are designed to handle more complex scenarios and may involve trade-offs in parameter settings, which could lead to suboptimal performance compared to methods specifically optimized for diploid assembly.

Because we believe that these additional experimental results are not directly relevant to the main focus of the manuscript, they have not been included in the revised version but are provided here for reference.

3. Since the authors have mentioned in their rebuttal that making use of the assembly graph is central to the tool, I think they should cite that Hifiasm has also implemented the same idea, but that GPhase can apparently be applied to polyploid genomes. This would be an added value worth highlighting.

Response:

We thank the reviewer for this valuable suggestion. As recommended, we have added the following sentence to the Introduction of the revised manuscript: "It is worth noting that although some diploid assembly tools, such as hifiasm, also leverage assembly graphs to assist 3C-based phasing, they do not perform well when directly extended to polyploid genomes, highlighting the intrinsic challenges of this problem."

4. In my opinion, the current title and introduction do not sufficiently reflect the use of the assembly graph, so it would be better if the authors could rephrase them accordingly. That said, the discussion is quite clear, e.g., the sentence at Lines 560–562 is a good summary of this contribution.

Response:

We thank the reviewer for pointing this out. In the revised manuscript, we have revised the title and the relevant statements in the Introduction to better reflect the role of the assembly graph, following the expression in Lines 560–562 of the original manuscript. The title has been updated to: "GPhase: Assembly Graph–Assisted Haplotype-Resolved Scaffolding of Polyploid Genome". In addition, the first sentence of the third paragraph in the Introduction has been revised as follows: "To overcome these limitations, we propose a new method that integrates a simplified assembly graph—produced during the unitig assembly step—to assist in the 3C-based phasing and scaffolding process. The assembly graph, which captures overlap relationships between long reads, provides complementary connectivity that mitigates 3C weaknesses."

5. Fig. 1 does not serve as a clear overview of the algorithm, and the legend is too concise to explain the figure. I think the authors should put more effort into making it accessible to a wide audience. Specifically, Fig. 1B has no explanation for the colors, numbers, and other visual elements.

Response:

As suggested, we have revised and expanded the legend of Fig. 1 to provide a more detailed explanation of the figure.

6. Fig. 3 has varying scales for the y-axes. It would be helpful to use the same scale where possible, to better reflect the differences among the three scaffolding tools.

Response:

As suggested, we have changed the y-axis scales of Fig. 3A and Fig. 3B from 0-100 to 0-1, making them consistent with Fig. 3C-F.

7. Line 61: the newly added word "ancient" is not accurate. Most if not all extant angiosperms are paleopolyploids, so this qualifier does not meaningfully narrow the scope. Please double-check the citation and the cited number.

Response:

We thank the reviewer for pointing this out. As suggested, we have removed the word "ancient" from the sentence. Reference 4 (a 1994 *Science* paper entitled "Stomatal size in fossil plants: evidence for polyploidy in the majority of angiosperms") reports that "most angiosperms (approximately 70 percent) have polyploidy in their history." We consider this to be consistent with our statement that "approximately 70% of angiosperms show evidence of polyploid ancestry." In the revised manuscript, to better align with the original wording, we have changed "more than" to "approximately."

8. Line 633–634: "In addition, non-3C data types, such as gamete sequencing data, may also be integrated in the future." This sentence is speculative without a concrete path forward. Consider removing it or elaborating on how this integration might be achieved.

Response:

As suggested, we have removed this sentence in the revised manuscript.

GPhase: Assembly Graph-Assisted Haplotype-Resolved Scaffolding of Polyploid Genome

Dear Editor,

We sincerely thank you and the reviewers for their careful evaluation of our manuscript, “GPhase: Assembly Graph-Assisted Haplotype-Resolved Scaffolding of Polyploid Genome”(NPLANTS-250920876B). We greatly appreciate the reviewers’ positive feedback and constructive suggestions, which have helped us further improve the usability and presentation of GPhase.

In this final revision, we have further improved the GPhase workflow based on the reviewers’ suggestions. Specifically, we have streamlined the organization of the final output results, improved the documentation for downstream assembly visualization and manual curation, and enhanced the overall usability and accessibility of the software.

We have carefully addressed the remaining comments and made corresponding revisions as described below.

We look forward to your feedback.

Weihua Pan

Professor

Agricultural Genomics Institute at Shenzhen

Chinese Academy of Agricultural Sciences

Reviewer #1

Comment:

The authors have addressed the issue reported in my previous review, and I was able to successfully run the workflow on my pentaploid dataset.

I did not have sufficient time to perform an in-depth assessment of the resulting assembly and phasing quality. However, the workflow now runs smoothly, and the results appear promising based on a first inspection.

Overall, I believe the authors have satisfactorily addressed the reviewers' comments, and I support publication of the manuscript. I would again like to congratulate the authors for developing and maintaining a valuable tool that will help the community generate high-quality phased assemblies for polyploid species.

Response:

We sincerely thank Reviewer #1 for testing GPhase on a pentaploid dataset and for the positive assessment of the revised workflow. We appreciate the reviewer's support and recognition of the value of GPhase for polyploid genome assembly.

Reviewer #2

Comment:

Comments for the Author:
In the revision, the authors have addressed all my previous remarks. I have also tested the tool with two polyploidy species sequenced in house and it can run smoothly in the end. Based on the results, I again think this tool is a valuable contribution to the community.

Remarks on code availability:
I did not review the code line by line but installed the tool and ran it on a dataset in house. In general, it was straightforward to install in the current round of revision, and after fixing some issues, we could run the code and get the results.

Response:

We sincerely thank Reviewer #2 for testing GPhase on independent polyploid datasets and for the positive feedback. We appreciate the reviewer's recognition of the utility of GPhase. We have further improved the software package and documentation to enhance the accessibility and reproducibility of GPhase.

Reviewer #3

Comment:

Although I could run GPhase successfully, I encountered an important usability gap when attempting to manually refine the assembly using Juicebox. The rename_collapse_agp_pairs_fasta.py and juicebox.sh scripts in GPhase generate three key files: final_hic.assembly (with renamed ctg-style identifiers, e.g., ctg00000001.1), rename.fa (containing original unitig names like utg000404l and utg000404l_dup1), and final_hic.liftover.agp (which maps each ctg name to its corresponding unitig). However, the current documentation does not clearly explain the relationships among these files. As a result, when users wish to manually export the modified genome using juicebox_assembly_converter.py -a final.review.assembly -f $genome, they are left unsure which FASTA file should be supplied as -f (the converter expects ctg-compatible headers).

Therefore, I kindly suggest that the authors improve the "Generate a Hi-C heatmap" section in the README by:

1. Clearly documenting the roles of final_hic.assembly, rename.fa, and final_hic.liftover.agp, and explaining that the latter provides the mapping from the ctg names to the raw unitig names.
2. Providing a short example or helper script that reconstructs a ctg-named FASTA from rename.fa using the liftover AGP, enabling seamless use of juicebox_assembly_converter.py.

These documentation can would greatly improve the post-Juicebox curation workflow in GPhase, making the tool even more user-friendly.

Response:

We sincerely thank Reviewer #3 for this valuable suggestion. Following the reviewer's comments, we have further improved the usability of GPhase by refining the organization of the final outputs and enhancing the documentation for downstream Hi-C visualization and manual assembly curation. The updated README provides clearer guidance on the workflow and facilitates a smoother transition from GPhase results to subsequent Juicebox-based assembly refinement. We appreciate the reviewer's suggestion, which has helped us improve the accessibility and user experience of GPhase.
